# Cancer-associated fibroblasts are the main contributors to epithelial-to-mesenchymal signatures in the tumor microenvironment

**DOI:** 10.1038/s41598-023-28480-9

**Published:** 2023-02-21

**Authors:** Peter M. Szabo, Amir Vajdi, Namit Kumar, Michael Y. Tolstorukov, Benjamin J. Chen, Robin Edwards, Keith L. Ligon, Scott D. Chasalow, Kin-Hoe Chow, Aniket Shetty, Mohan Bolisetty, James L. Holloway, Ryan Golhar, Brian A. Kidd, Philip Ansumana Hull, Jeff Houser, Logan Vlach, Nathan O. Siemers, Saurabh Saha

**Affiliations:** 1 Bristol Myers Squibb, Princeton, NJ USA; 2 Present Address: Fate Therapeutics, San Diego, CA USA; 3 Dana-Farber Cancer Institute, Boston, MA USA; 4 Present Address: Merck & Co., Inc., Kenilworth, NJ USA; 5 Bristol Myers Squibb, San Diego, CA USA; 6 Bristol Myers Squibb, Cambridge, MA USA; 7 Present Address: Daiichi Sankyo, Inc., Princeton, NJ USA; 8 Bristol Myers Squibb, Seattle, WA USA; 9 Bristol Myers Squibb, Redwood City, CA USA; 10 Present Address: Vanderbilt University, Nashville, TN USA; 11 Present Address: Fiveprime Group, Monterey, CA USA; 12 Present Address: Centessa Pharmaceuticals, Cambridge, MA USA

**Keywords:** Gene expression profiling, Immunohistochemistry, Cancer microenvironment, Tumour biomarkers

## Abstract

Epithelial-to-mesenchymal transition (EMT) is associated with tumor initiation, metastasis, and drug resistance. However, the mechanisms underlying these associations are largely unknown. We studied several tumor types to identify the source of EMT gene expression signals and a potential mechanism of resistance to immuno-oncology treatment. Across tumor types, EMT-related gene expression was strongly associated with expression of stroma-related genes. Based on RNA sequencing of multiple patient-derived xenograft models, EMT-related gene expression was enriched in the stroma versus parenchyma. EMT-related markers were predominantly expressed by cancer-associated fibroblasts (CAFs), cells of mesenchymal origin which produce a variety of matrix proteins and growth factors. Scores derived from a 3-gene CAF transcriptional signature (*COL1A1*, *COL1A2*, *COL3A1*) were sufficient to reproduce association between EMT-related markers and disease prognosis. Our results suggest that CAFs are the primary source of EMT signaling and have potential roles as biomarkers and targets for immuno-oncology therapies.

## Introduction

Epithelial-to-mesenchymal transition (EMT) is a process of tissue dedifferentiation occurring during development and tissue repair that results in epithelial cells transitioning into cells with a more mesenchymal phenotype^1–4^. Depending on the contextual signals received by the cell within a tissue, a series of intermediate phenotypic states may be generated along the epithelial-mesenchymal spectrum up to a fully mesenchymal cell state^4^. Tumor cells may induce this transition to drive tumor growth and initiate metastasis^5^. Given the important role of this process in cancer progression, a variety of gene expression signatures that characterize EMT have been identified^6–11^.

EMT-related gene expression has been shown to impact T-cell function and infiltration into the tumor parenchyma^10^. The extent of lymphocyte infiltration is influenced by stromal factors within the tumor microenvironment (TME), such as transforming growth factor beta (TGFβ). TGFβ signaling has been shown to inhibit T-cell proliferation and regulate T-cell effector functions, and it has been implicated as a contributor to the acquisition of a cancer-associated fibroblast (CAF) phenotype in stromal cells^12–15^. CAFs have been suggested to originate from mesenchymal stromal cells, among others^16^, and shown to exert diverse tumor-promoting functions in the context of the TME, notably tumorigenesis, cancer cell proliferation and metastasis, extracellular matrix remodeling, drug resistance, immune evasion, and immunosuppression^14,17–21^. Additionally, TGFβ signaling and collagen production by CAFs^22^ may contribute to the exclusion of T cells from the tumor parenchyma^23^.

Previous studies have found that tumors with high baseline expression levels of immune-related genes in the TME were more likely to respond favorably to immuno-oncology (I-O) therapy^24–26^. In tumors with high levels of inflammation, gene expression signature scores indicative of lower EMT and stromal signaling have been shown to be associated with increased response rates and prolonged survival in patients with urothelial carcinoma (UC) treated with the programmed death 1 (PD-1) inhibitor nivolumab^10,27^. In patient-derived xenograft (PDX) models of UC, EMT-related gene signatures were shown to be strongly correlated with stroma-related gene signatures, implying that stromal cells may be a key source of EMT-related gene expression^10^. Of note, these models have been shown to have many advantages compared with other models, such as retaining the characteristics of the donor tumor, including tissue structure and gene expression profiles, and therefore are potentially predictive of human tumor biology and patient response to treatment^28–33^. Separate work done in colorectal cancer (CRC) using single-cell RNA sequencing (scRNA-seq) demonstrated that tumors with a strong epithelial signature and weak fibroblast and myeloid signatures had the best survival rate^34^. Notably, in this study, expression of EMT-related genes was significantly increased in CAFs^34^.

The role of EMT signaling has been extensively studied using preclinical models of individual tumor types. While previous evidence suggests that EMT signaling is emanating from cells within the stromal compartment^10^, there is ongoing research into the role of specific cell types, such as CAFs, in the EMT process and their associations with factors that can influence response to I-O therapy. In the current study, bulk and single-cell RNA sequencing analysis and immunohistochemistry (IHC)-based methods were used to investigate the source of EMT- and stroma-related gene expression across multiple tumor types and to determine factors in the stromal compartment of the TME that may associate with prognosis or response to treatment.

## Results

### Correlation between EMT- and TME-related gene expression signatures

**Figure 1:**
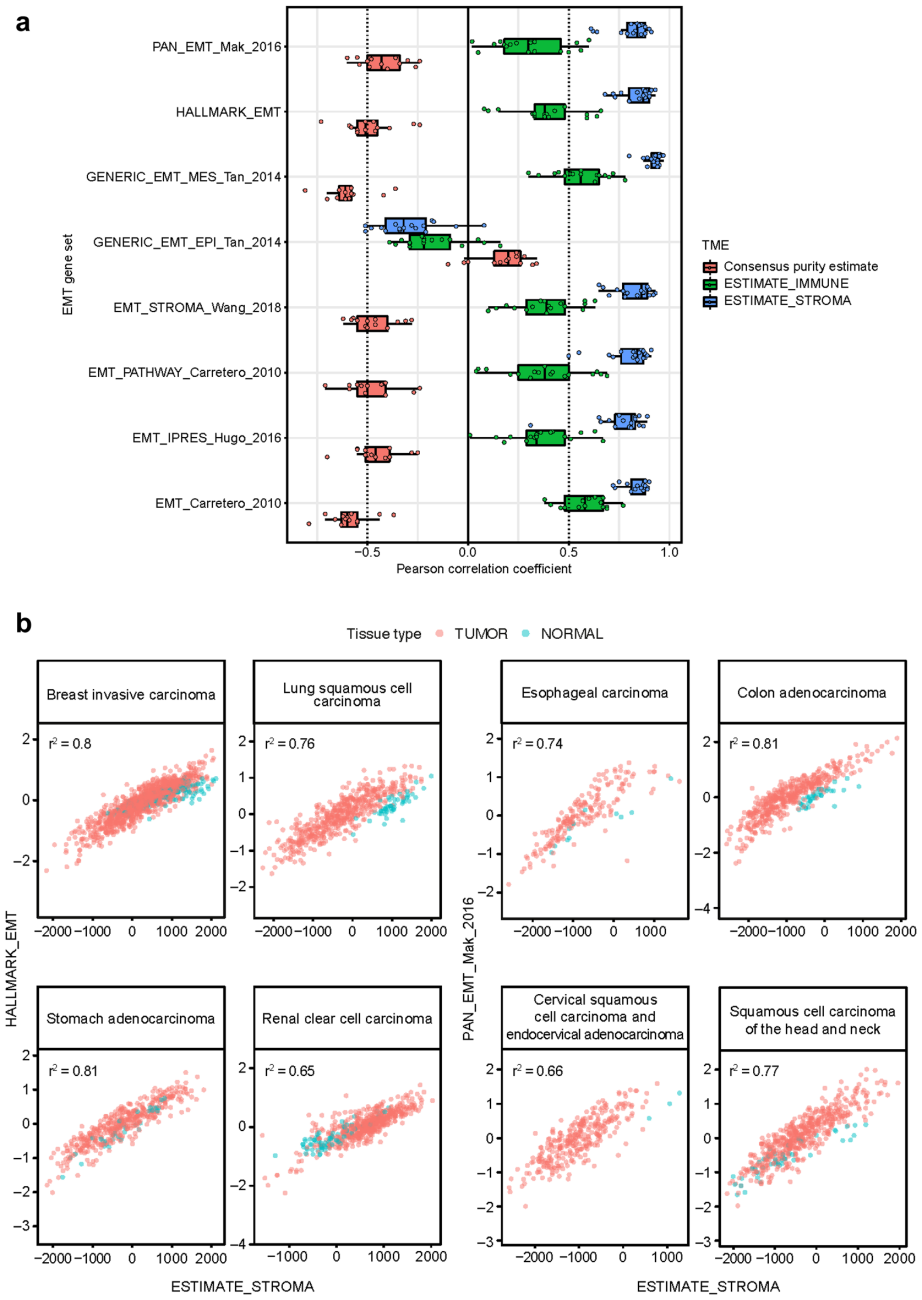
EMT signatures and the TME. (**a**) Distribution over tumor types in TCGA of estimated correlation (Pearson coefficients) between scores for EMT-related gene sets and tumor purity, an immune gene expression signature, and a stroma signature. Boxes extend from the first to third quartiles, the middle line shows the median, and the whiskers extend to the most extreme data point that is no more than 1.5 times the IQR from the box. Consensus purity estimates were not available for esophageal carcinoma, pancreatic and stomach adenocarcinoma, and sarcoma. (**b**) Scatterplots of scores for two EMT-related gene signatures versus a stroma signature, for selected tumor types in TCGA. Pink plotting symbols represent tumor tissue. Aqua plotting symbols represent samples collected from non-involved healthy tissue of patients with cancer in TCGA.

In RNA-seq data for 17 tumor types in The Cancer Genome Atlas (TCGA), scores for 7 of 8 previously published EMT gene signatures (EMT_Carretero_2010, IPRES_Hugo_2016, PATHWAY_Carretero_2010, EMT_STROMA_Wang_2018, GENERIC_EMT_MES_Tan_2014, HALLMARK_EMT, and PAN_EMT_Mak_2016)^6–11^ were negatively correlated with scores for a tumor purity signature and positively correlated with scores for immune and stroma signatures (Fig. 1a, Supplementary Fig. 1, and Supplementary Table 1). Correlations tended to be smaller and in the opposite direction for the GENERIC_EMT_EPI_Tan_2014^7^ gene set, which is characteristic of epithelial features rather than mesenchymal features (Fig. 1a). A strong positive correlation was observed between EMT gene set signature scores and estimates of stromal content in samples from TCGA across all tumor types with epithelial origin, including colon, stomach, urothelial, and breast (Fig. 1b, Supplementary Fig. 1, and Supplementary Table 1). In tumors with a mesenchymal origin, such as sarcoma, these correlations were weaker (Supplementary Fig. 1n).

### EMT-related genes enriched in the stromal compartment of PDX samples

**Figure 2:**
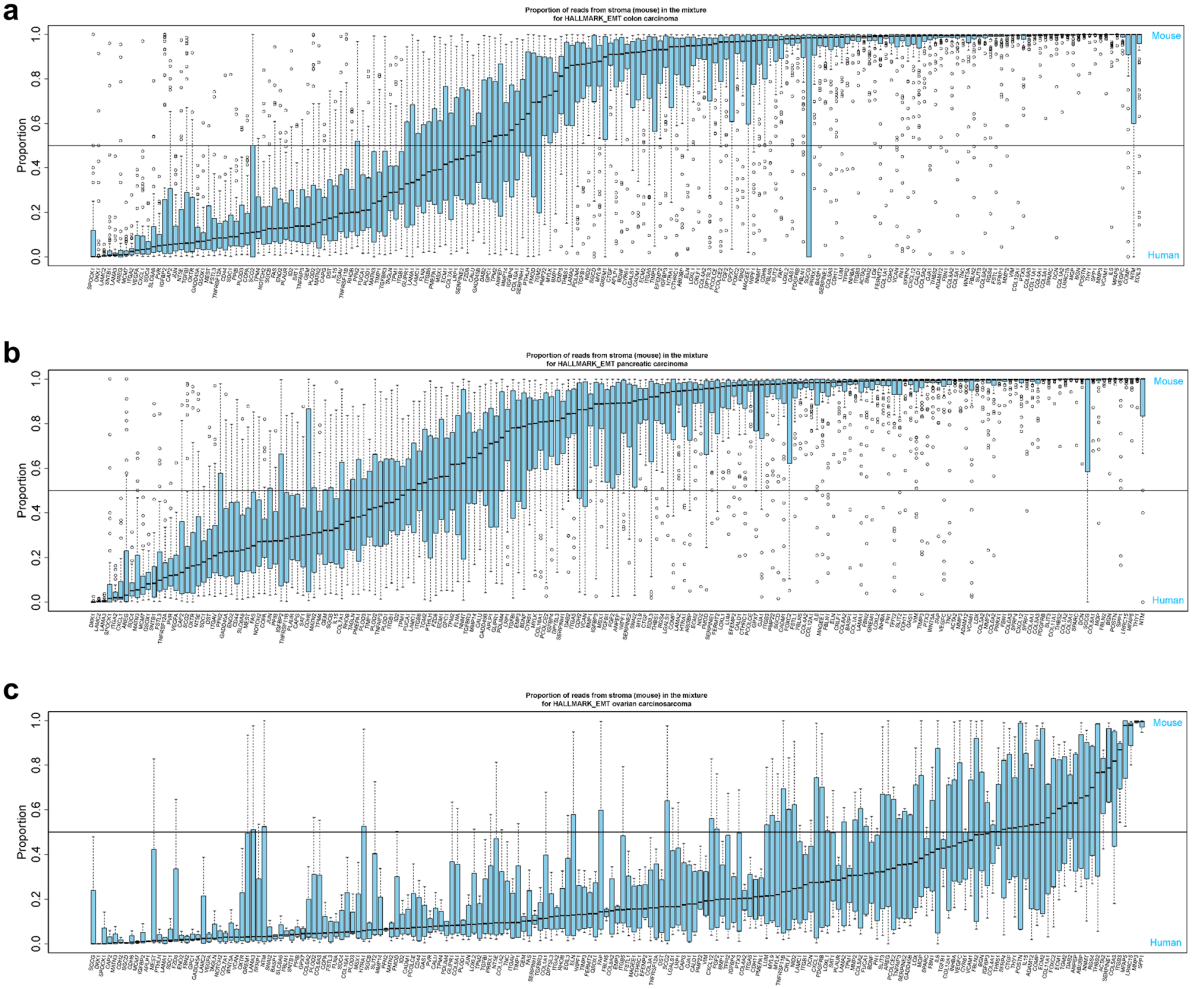
Expression of HALLMARK_EMT-related genes in mouse (stromal) and human (tumor parenchymal) tissues using PDX samples. Horizontal reference line indicates proportion of reads from stroma = 0.5 (signifying equal contribution from stromal and cancer cells to the estimated gene expression). Boxes extend from the first to third quartiles, the middle line shows the median, and the whiskers extend to the most extreme data point that is no more than 1.5 times the interquartile range from the box, open circles show individual values that are more than 1.5 times the interquartile range from the box. (**a**) Colon (*n* = 70). (**b**) Pancreatic (*n* = 57). (**c**) Ovarian carcinosarcoma (*n* = 4). Additional tumor types and gene sets are shown in Supplementary Fig. 2. These figures demonstrate that, for multiple tumor types, a greater proportion of transcripts of EMT-related genes were derived from stromal tissue than from tumor tissue, with the exception being the GENERIC_EMT_EPI_Tan_2014 gene set (shown in Supplementary Fig. 2f.).

Given the above findings, we sought to elucidate the source of EMT-related gene expression in the tumor parenchyma or stromal cells. Expression of genes in each of the 8 selected EMT gene sets and the ESTIMATE_STROMA gene set^6–11^ were analyzed in RNA-seq data from PDX samples representing individual tumor types and containing tissue derived from the stromal (mouse) and tumor (human) compartments of the TME. While variability was observed across tumor types in the proportion of EMT-related gene expression derived from the stroma, in many tumor types, a substantial proportion of transcripts of EMT-related genes were derived from stromal tissue (Fig. 2, Supplementary Fig. 2, and Supplementary Table 2). The exception was the GENERIC_EMT_EPI_Tan_2014 gene set (Supplementary Fig. 2f.), which represents epithelial cells before transition to mesenchymal cells; it is expected that these genes would originate from the tumor parenchymal tissue. Evidence of stromal origin for most of the transcripts in the EMT gene sets was not observed for ovarian carcinosarcoma or uterine carcinoma (Fig. 2c and Supplementary Fig. 2), likely due to their mesenchymal origin or presence of intrinsic mesenchymal components. Despite this, we detected significant enrichment of stromal transcripts in the majority of EMT gene sets (Supplementary Table 2), suggesting measurable contributions from stromal cells in gene expression estimates in these cancer types. As a positive control, a large majority of transcripts of genes in the ESTIMATE_STROMA gene set were derived from stromal tissue for all tumor types, including ovarian carcinosarcoma (Supplementary Fig. 2e).

### EMT-related genes expressed by CAFs, endothelial cells, and myeloid progenitor cells

**Figure 3:**
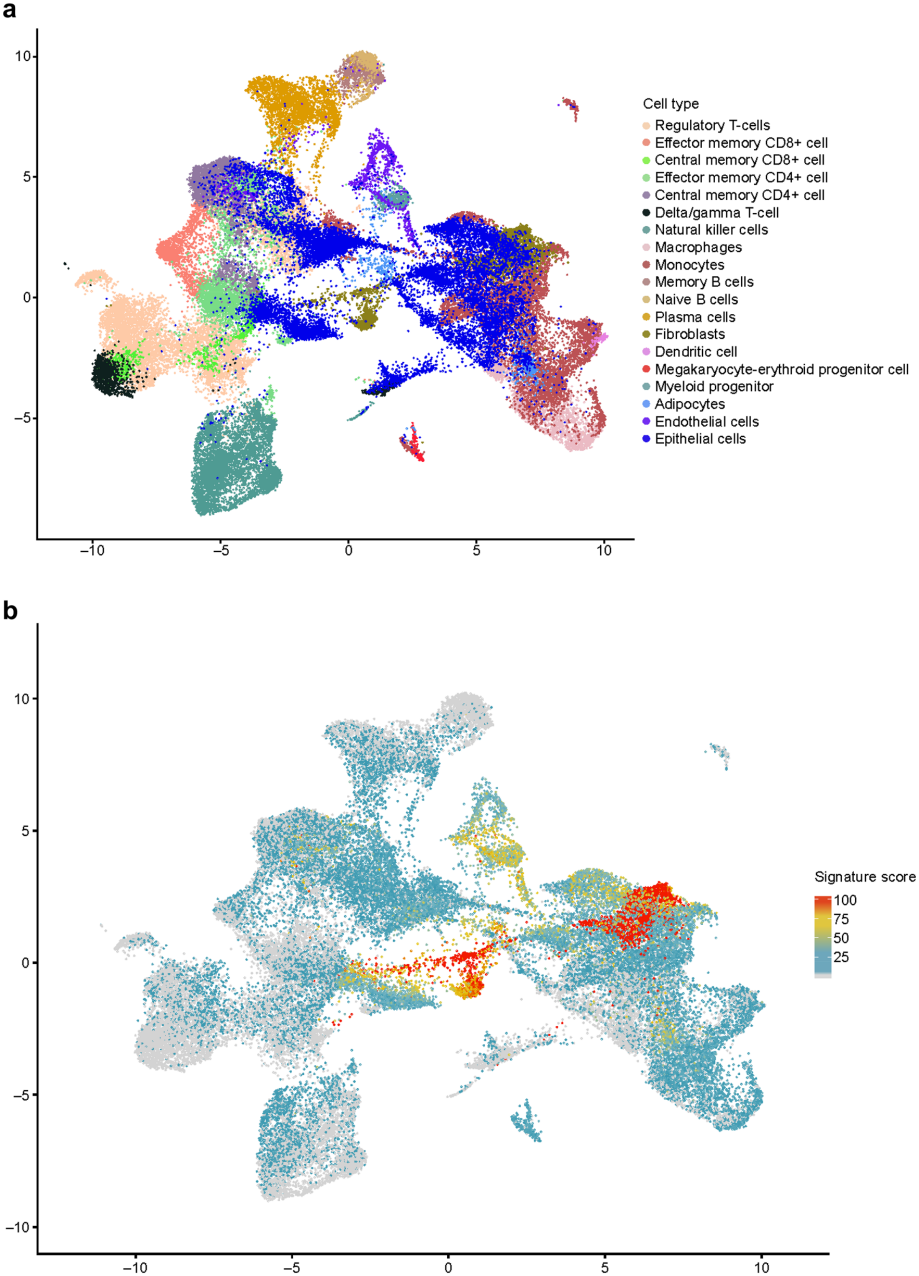

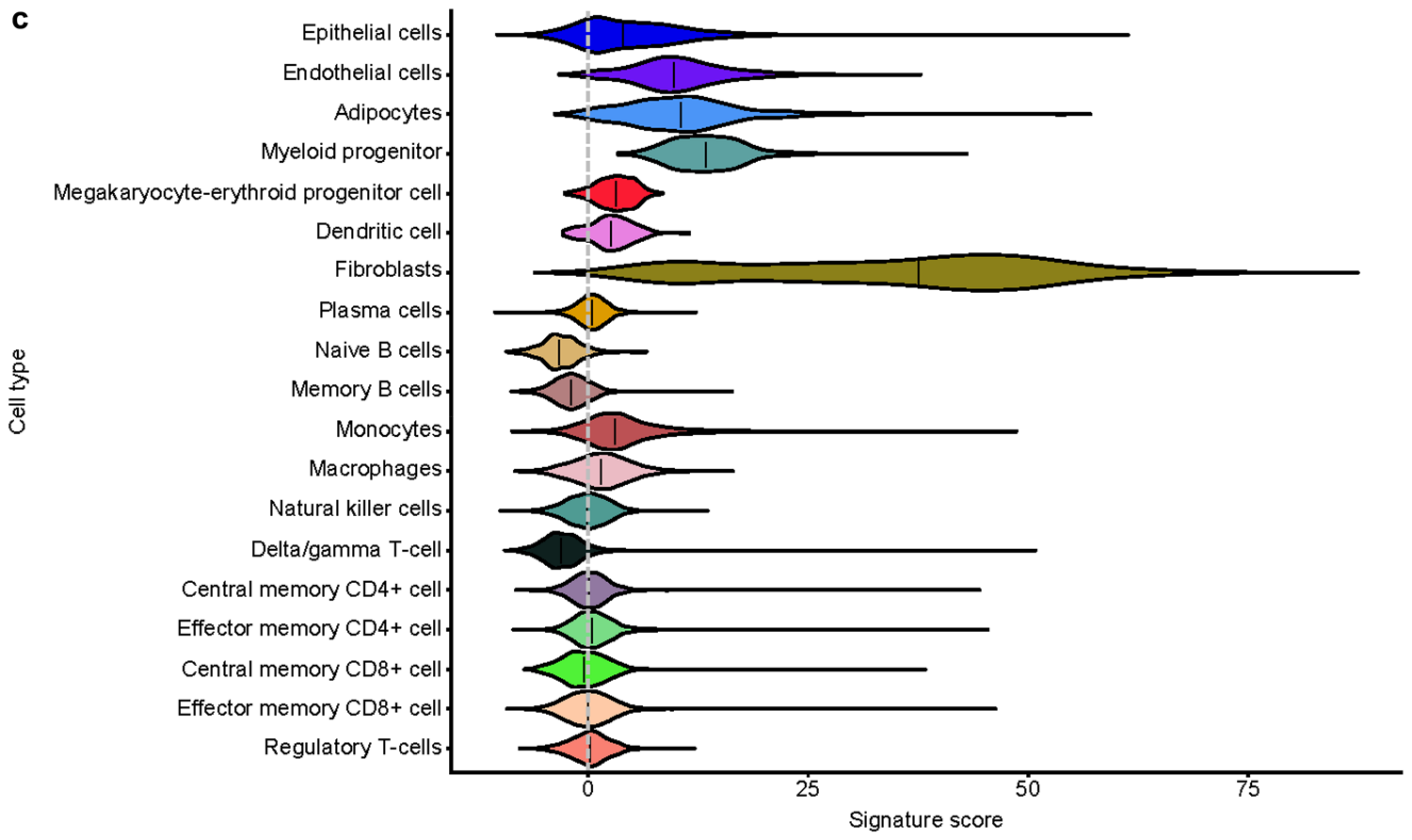
scRNA-seq of tumor samples. (**a**) Identification of cell type in individual scRNA-seq datasets displayed as a 2-dimensional projection. (**b**) Location and expression level of HALLMARK_EMT signature genes in scRNA-seq datasets displayed as a 2-dimensional projection. (**c**) HALLMARK_EMT-related gene expression by cell type. In (**b**) and (**c**), the gene signature scores (0–100) represent the gene expression level for the EMT signatures in the scRNA-seq data. Analysis of additional EMT gene set signatures showed greater fold enrichment in fibroblasts and other non-malignant stromal cells than from tumor tissue, with the exception being the GENERIC_EMT_EPI_Tan_2014 gene set (data not shown).

The types of cells in samples from 14 treatment-naive patients with colorectal or renal carcinoma were identified using canonical correlation analysis of scRNA-seq profiles, shown in Fig. 3a as a two-dimensional projection. After filtering for poor quality, a total of 56,667 cells across 32 resected tumor and adjacent normal tissue samples were selected for downstream analysis. EMT signature gene expression was scored for each scRNA-seq dataset and applied to the projection to identify cell types with enriched expression of EMT signatures. The greatest fold enrichment was observed in fibroblasts; other enrichments were observed to a lesser extent in adipocytes, epithelial, endothelial, and myeloid progenitor cells (example shown in Fig. 3b,c for the HALLMARK_EMT gene set).

Median scores for the EMT-related gene sets used in this study were determined in the scRNA-seq datasets identified as fibroblasts or as epithelial tumor parenchymal cells. Across a select range of EMT-related gene sets, the median scores for the EMT signatures (with the exception of those in the GENERIC_EMT_EPI_Tan_2014 gene set) were greater (*p* < 0.01) in fibroblasts found in the stroma than in tumor parenchymal cells (Supplementary Fig. 3).

### Strong positive correlation between EMT and CAF gene expression signature scores and association with overall survival (OS)

**Figure 4:**
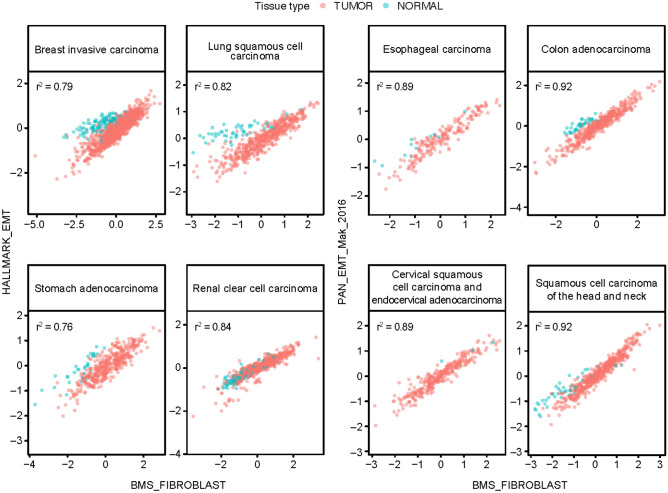
Correlation between EMT-related and CAF (BMS_FIBROBLAST) signature scores in tumor datasets from TCGA. Pink plotting symbols represent tumor tissue. Aqua plotting symbols represent samples collected from non-involved healthy tissue of patients with cancer from TCGA.

To further support the hypothesis that CAFs are a major source of EMT gene expression, we developed a “CAF signature” derived from the expression of 3 collagen genes (*COL1A1*, *COL1A2*, and *COL3A1*), as described in the Methods, and assessed the correlation between EMT-related and CAF gene expression in samples from TCGA. Across multiple tumor types, a strong positive correlation was observed between scores for all the EMT gene sets (except GENERIC_EMT_EPI_Tan_2014) and CAF signature scores (Fig. 4, Supplementary Fig. 4, Supplementary Table 3). As expected, the positive correlations were somewhat weaker for sarcoma (Supplementary Fig. 4n, Supplementary Table 3).

**Figure 5:**
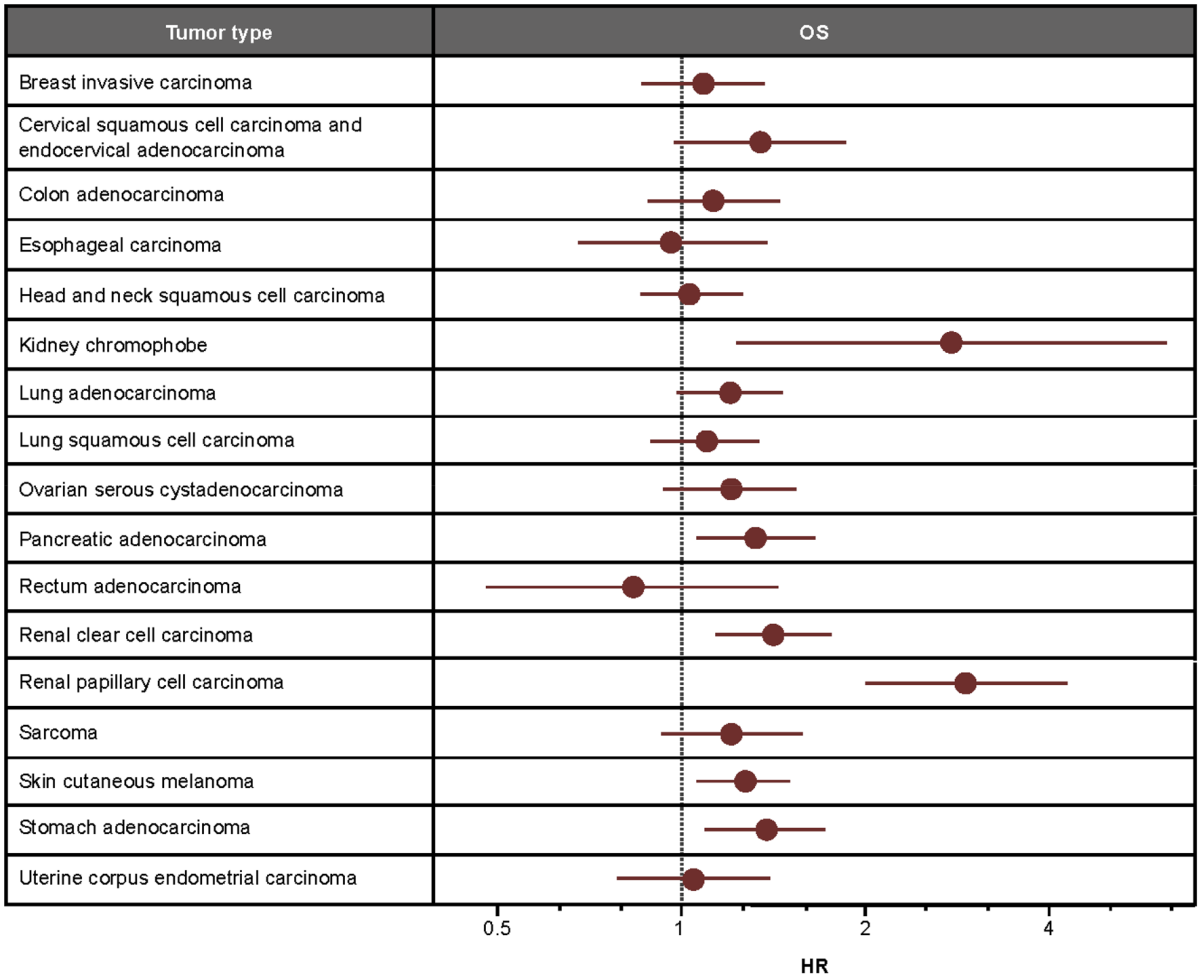
Association between CAF signature scores and OS. HRs were estimated from TCGA datasets using Cox proportional hazards models assuming a linear effect of signature scores. Estimated HRs (high signature score/low signature score) were scaled to compare hazards for scores differing by the IQR for each tumor type.

In these same tumor types, lower CAF signature scores tended to be associated with prolonged survival (Fig. 5). As expected, given the strong positive correlations observed, associations between OS and CAF signature scores were similar in magnitude and direction to associations between OS and EMT signature scores (Supplementary Table 4). This is consistent with previous literature regarding associations between OS and EMT gene expression^10^.

### Collagen scores inversely associated with parenchymal T-cell counts in squamous cell carcinoma of the head and neck (SCCHN)

**Figure 6:**
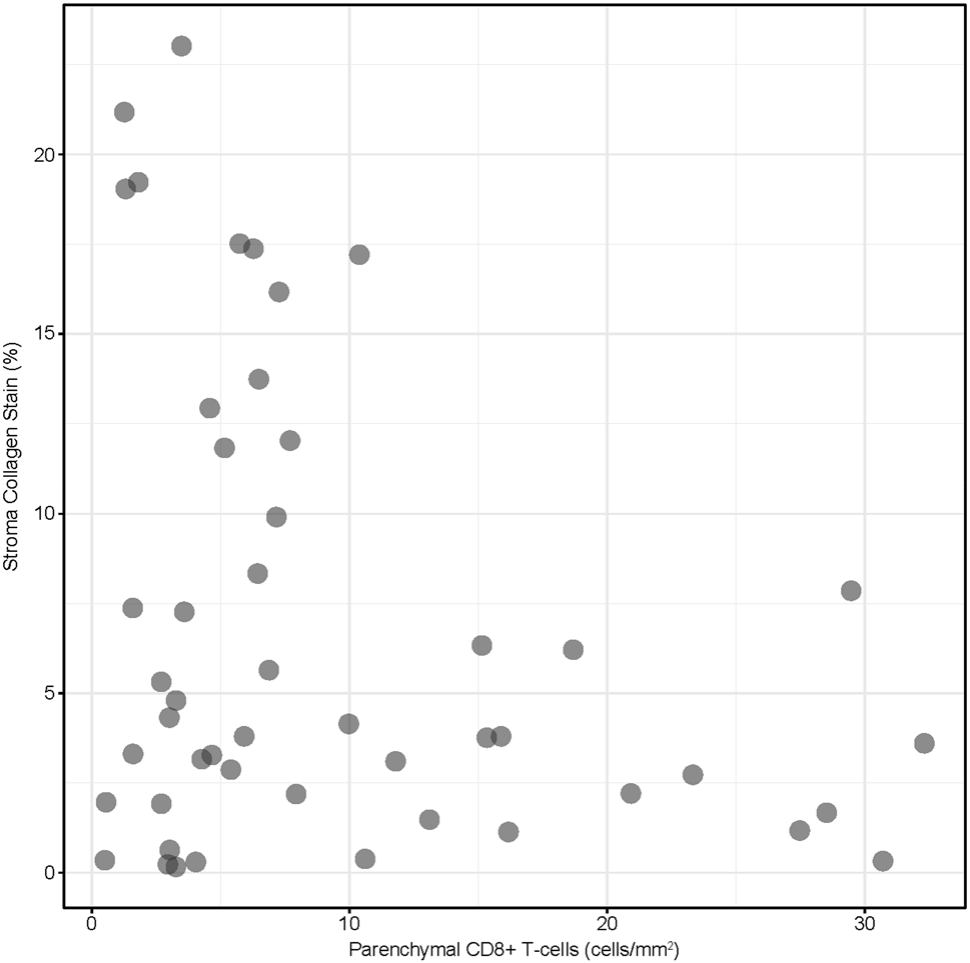
Association between collagen and parenchymal CD8+ T-cell abundance in SCCHN tumor samples. Collagen staining was assessed by image analysis to identify tumor parenchymal and stromal compartments and calculate the % area of collagen staining relative to the area of the total stromal compartment. An AI-powered image analysis algorithm was used to classify the tumor parenchymal and stromal regions and identify and quantify the density (cells/mm^2^) of CD8+ T cells in each compartment.

We then sought to investigate possible mechanisms behind the link between CAF scores and prognosis. Using IHC, we studied the relationship between collagen abundance in the tumor stroma and exclusion of CD8+ T cells from the tumor parenchyma in SCCHN tumor specimens. Low stromal collagen scores were observed in samples with high parenchymal CD8+ T-cell counts, while high collagen scores were observed in samples with low levels of parenchymal CD8+ T cells. Samples with low levels of both stroma collagen and parenchymal CD8+ T cells were also observed; however, no sample had high levels of both stroma collagen and parenchymal CD8+ T cells (Fig. 6). Representative IHC images are shown in Supplementary Fig. 5.

### Correlation between TGFβ- and TME-related gene expression signatures

Given the known role of TGFβ signaling in the acquisition of a CAF phenotype^14^, we analyzed various TGFβ-related signatures, including the Hallmark_TGF_BETA_SIGNALING and GO_TRANSFORMING_GROWTH_FACTOR_BETA_BINDING gene sets using RNA-seq data from TCGA. These signatures were obtained from previously published literature^11,35–38^ or retrieved from an online database as described in the methods. These signatures were negatively correlated with tumor purity and positively correlated with stromal signature scores (Supplementary Fig. 6), similar to the patterns observed for the EMT signature scores. A smaller positive correlation between TGβ-related signatures and immune signature scores was also observed (Supplementary Fig. 6).

To further investigate the cellular source of TGFβ signaling, expression scores were determined for the TGFβ-binding protein gene signatures and were applied to our scRNA-seq projections to identify which cell types were enriched for expression of TGFβ-related genes (Fig. 3a). Expression of TGFβ-related genes was strongly enriched in myeloid progenitor cells, endothelial cells, and CAFs, relative to other cell types (Supplementary Fig. 7a, b). Similar to our results regarding EMT-related gene expression, TGFβ-related gene expression was greater on average in stromal cells than in parenchymal cells.

## Discussion

**Figure 7:**
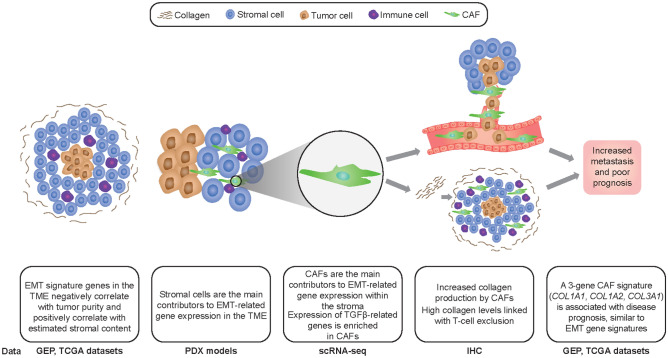
Overview of the main findings from this study. Top images provide graphical representation of the TME. Bottom text describes the main results and corresponding experimental datasets and methods. GEP, gene expression profiling.

EMT is a critical step for cancer cell metastasis, drug resistance, and immune evasion^2^. In this study, we sought to determine the source of EMT-related gene expression within the TME. A graphical representation of our overall findings is presented in Fig. 7. According to prior literature supporting tumor cells as the source of EMT-related gene expression^39^, one would assume tumors become more resistant to immune cell infiltration as they go through the EMT process and begin to express mesenchymal genes. One would therefore expect expression of EMT genes to be positively correlated with the number of cancer cells present in the tumor and negatively correlated with immune and other stromal cell content. Instead, using gene expression profiling of bulk tumor tissues, the opposite was observed across a variety of tumor types: a strong negative correlation between multiple EMT gene set signatures and proportion of cancer cells, and a strong positive correlation between these same gene set signatures and stromal content. This suggests that EMT gene signatures largely are not measuring tumor cell–related mechanisms, but rather the amount of stromal content in the TME. The exception was the GENERIC_EMT_EPI_Tan_2014 gene set, which did not show strong correlations with signatures of tumor purity, stroma, or immune components of the TME. This could be because, in our study, most of the analyzed EMT gene sets measured mesenchymal features of the tumor cells, whereas this signature was chosen by Tan et al. to represent polarized epithelial cells before the transition to mesenchymal cells^7^. Tumor cells with epithelial origins, such as carcinomas, already express epithelial markers and could therefore display positive correlations with the GENERIC_EMT_EPI_Tan_2014 gene set and tumor purity.

Based on these findings, we hypothesized that stromal elements are the key contributors to EMT expression in the TME. Using a species-specific approach to parse mouse and human reads in a pan-cancer collection of gene expression data from xenograft samples, we demonstrated that, in the majority of tumor types, a greater proportion of transcripts from EMT-related genes originated from mouse stromal cells than from human tumor cells. In cancers known to have mesenchymal lineage components, such as ovarian carcinosarcomas^40^, expression of EMT-related genes did not originate predominantly from mouse stromal regions. Analysis of individual cell transcriptomes from tumor samples by scRNA-seq demonstrated that these EMT-related genes were highly expressed by CAFs. Our results are consistent with other recent studies examining EMT and CAFs using similar methods. In a study by Puram et al., scRNA-seq was used to generate profiles for 18 SCCHN primary tumors^41^. Similar to our study, genes used to identify a mesenchymal phenotype were found to be expressed by CAFs. This was further supported by Zhang et al., who used two scRNA-seq platforms to classify immune and stromal cell populations and evaluate cell–cell interactions in CRC tumors and adjacent normal tissues. In addition to CAFs being enriched in the stromal tissue compared with normal tissue, certain subsets of tumor-associated macrophages were found to preferentially bind to CAFs and endothelial cells leading to activation of *MMP2*, a gene associated with tumor growth and metastasis^42^. These interactions highlight a potential mechanism by which CAFs can be regulated to drive tumor growth and metastasis.

To further validate CAFs as the source of EMT-related gene expression, we used fibroblast activation protein (previously established as a CAF-specific marker gene) as a sentinel marker and collagen genes, representing one of the fundamental functions of CAFs (extracellular matrix production), to generate a 3-gene CAF signature. Using collagen genes in preference to other genes associated with the CAF phenotype, such as α-smooth muscle actin, platelet-derived growth factor receptor β, and secreted protein acidic and rich in cysteine (SPARC)^15^, allowed us to use histological techniques (e.g. collagen quantitation) to investigate the biological mechanisms underlying the expression of EMT-related genes by CAFs. High correlation was observed between the EMT gene sets and our CAF signature when compared in bulk tumor tissue from TCGA, providing additional evidence that CAFs, rather than epithelial cells, are the primary source of EMT-related gene expression. Given the role of EMT in metastasis, drug resistance, and immune evasion, it is important to determine any clinical implications of EMT signature scores on patient survival and possible biological mechanisms behind these findings. Having observed a correlation between EMT and CAF signatures, we performed a retrospective analysis of patient data in TCGA. We found that CAF signature scores, similar to EMT signature scores, were negatively associated with overall survival, demonstrating the prognostic value of the CAF signature.

Preliminary exploration of the biological mechanisms involved in the association of EMT signature scores with immune cell infiltrates and signatures have shown that accumulation of CAFs in the tumor stroma is associated with decreased CD8+ T-cell infiltration, while the depletion of CAFs increases intratumoral T-cell concentrations^43–45^. Our results in SCCHN showed that collagen, produced by CAFs in the TME^22^, was also associated with T-cell exclusion and may thereby contribute to resistance to I-O therapy. Similar to these results, high expression of CAF and EMT-related genes was associated with T-cell exclusion in UC^10^, ovarian cancer^46^, and hepatocellular carcinoma^47^. These findings support the possibility of using trichrome staining for collagen quantitation as a surrogate for CAF content.

Given that TGFβ signaling is the most well-characterized pathway known to induce EMT, acts through various intracellular messengers to drive immunosuppression, and is one of the main regulators of collagen synthesis, fibrosis, and conversion of normal fibroblasts to a CAF phenotype^15,48–50^, we investigated TGFβ-related gene expression in the stromal and immune compartments of the TME. We demonstrated that genes coding for TGFβ-binding proteins are expressed by multiple cells within the stromal region, including endothelial cells, CAFs, and myeloid progenitors. These results are supported by previous studies examining TGFβ signaling in stromal cells. In a study using UC tumors from a large cohort of patients treated with the anti–PD-1 agent atezolizumab, response was associated with CD8+ T-cell prevalence, while lack of response was associated with TGFβ signaling in fibroblasts and T-cell exclusion^23^. Along with our results, this suggests a possible role for combining TGFβ inhibitors with immune checkpoint inhibitors to increase clinical benefit in patients with solid tumors.

There are some potential limitations to our study. Although our robust xenograft model provides further evidence of EMT signaling in the stromal region, interpretation is limited by the potential lack of a normal complement of immune cells in immunocompromised murine hosts. However, our scRNA-seq results demonstrated that EMT gene expression was greater in fibroblasts than in immune cells, suggesting that this is not a major issue. The lack of a comparator arm in our survival analysis using samples from TCGA limits the ability to determine a predictive versus prognostic nature of the CAF signature score, and prospective validation of these results will be required. It should also be noted that prior studies in solid tumors have shown that tumor cells at the invasive front also display mesenchymal features and may potentially be another source of EMT expression^41,51^. This type of signaling may be diminished when using bulk RNA-seq methods but may explain the observed moderate increase in EMT expression in a subset of epithelial cells in our scRNA-seq analysis.

The results of our study provide comprehensive evidence that CAFs and other stromal cells, rather than tumor parenchymal cells as previously thought, are the predominant sources of EMT-related gene expression in the TME and are major contributors to assessments of EMT gene signatures derived from bulk tumor tissue across a broad range of tumor types. These novel findings align with previous literature showing that upregulation of EMT-related transcription factors in CAFs leads to promotion of tumor growth and metastasis^52–54^. These results underscore the potential of studying CAFs as biomarkers for predicting response and resistance to immune checkpoint inhibitors. Given the important impact of these cells on tumor growth and immune cell infiltration, further study of these stromal cells may lead to the identification of novel targets for I-O therapies.

## Methods

### Analysis of gene expression data in TCGA

All tumor and normal tissue samples that were available from TCGA were used. To avoid bias no samples were excluded from the analysis, therefore the number of data points was not consistent between tumor types. RNA-seq data were analyzed using several previously published EMT- and TGFβ-related gene sets^6–11,35–38,55^. Additional TGFβ gene sets were retrieved from the KEGG PATHWAY (https://www.genome.jp/kegg/pathway/hsa/hsa04350.html), Mouse Genome Informatics (http://www.informatics.jax.org/go/term/GO:0005160), and Gene Set Enrichment Analysis (https://www.gsea-msigdb.org/gsea/msigdb/cards/BIOCARTA_TGFB_PATHWAY) databases. Signature scores were derived by z-scoring (within each tumor type) the expression values for each gene separately, and then for each sample taking the median of these z-scored expression values over the genes in a set.

The presence of stroma in tumor tissue and infiltration of T cells into the parenchyma were assessed using the ESTIMATE gene signatures. Consensus tumor purity estimates were used to assess tumor cell content in the TME. Details of both methods have been published previously^55^.

A 3-gene “CAF signature” was created as a transcriptional marker for fibroblasts. This set was derived by first evaluating co-expression of candidate sentinel markers that displayed selectivity of RNA expression for the target cell type with all transcripts across TCGA solid-tumor cohorts, details of which have been published previously^56^. Fibroblast activation protein is selectively expressed by CAFs in the TME and was therefore chosen as the sentinel marker^21,43,57^. We then used a stringent method of mutual rank distance to identify gene neighbors for the sentinel markers^58^. Based on these methods, *COL1A1*, *COL1A2*, and *COL3A1* were chosen as a gene set for the CAF signature.

### Gene expression profiling of PDX samples

Our study utilized previously published RNA-seq data on 333 PDX samples from 11 tumor types (colon, *n* = 70; pancreatic, *n* = 57; breast, *n* = 54; melanoma, *n* = 42; lung, *n* = 40; ovarian, *n* = 32; renal, *n* = 19; endometrial, *n* = 9; uterine, *n* = 4; sarcoma, *n* = 4)^59^. Bioinformatic analyses were then performed to distinguish transcripts from patient donor (tumor parenchyma) and mouse recipient (tumor stroma) as previously described^60^. For each tumor type, to assess whether expression of genes in a given EMT gene set^6–11^ was enriched in stroma-derived mouse transcripts, we first calculated the fraction of transcripts originating from mouse (the “mouse fraction”) for each gene in each sample. To account for potential differences in mouse content among individual samples, the mouse fraction for each gene was adjusted by subtracting the mean (over the genes) within each sample. Then, for each gene, we calculated the median (over the samples) of these adjusted mouse fractions. The genes were ranked by the medians, and we used these rankings as the input for gene set enrichment analysis^61^. Benjamini–Hochberg false discovery rates (FDRs) were used to adjust for multiple testing. The FDR adjustment was performed for each tumor type separately.

### scRNA-seq

#### Tissue procurement

Resected tumors and adjacent normal tissues were procured from BioIVT (BioIVT, Westbury, NY) from 14 treatment-naive patients with colorectal or renal cell carcinoma. After pathological analysis, all remaining tissue resections (> 0.6 g) were used to ensure tissue type representation.

#### Sorting

Tissue samples were enzymatically dissociated using a human Tumor Dissociation Kit (Miltenyi Biotec, Bergisch Gladbach, Germany) according to the manufacturer’s recommendations, and red blood cells were lysed using 1X RBC solution (eBioscience, Thermo Fisher Scientific, Waltham, MA, USA) for 5 min at room temperature before quenching with cold phosphate-buffered saline. Subsequently, single cell suspensions were Fc-blocked using TruStain FcX (1:125 dilution; BioLegend, San Diego, CA, USA) for 5 min on ice. Surface CD45 was stained with anti-CD45-FITC (1:50; #REA747, Miltenyi Biotech) for 20 min at 4 °C. Washed cells were stained with propidium iodide (PI; BD Biosciences, Franklin Lakes, NJ, cat: 556463) and with DRAQ5 (#62251, Thermo Fisher Scientific, Waltham, MA, USA) diluted 1:5000 in cold 1% bovine serum albumin in phosphate-buffered saline and stained for 10 min at room temperature. Single cells were sorted for live, nucleated CD45+ and CD45− cells to enrich for immune and non-immune cells.

#### Sequencing

Consecutive single cell libraries were prepared using the Chromium Single Cell Controller, version 3.16 (10 × Genomics, Pleasanton, CA) according to the respective protocol. Target cell number was 5000 cells per sample, and sequencing depth was 80,000 reads per cell. All data was merged and filtered for high-quality sequencing data using thresholds (nFeature_RNA > 200 & nFeature_RNA < 6000 & mito.read.fraction < 0.15 & nCount_RNA < 15,000). Expression counts were normalized using SCTransform^62^ and aligned using canonical correlation analysis, with uniform manifold approximation and projection (UMAP)^63^ used for visualization. After clustering, cell types were identified (SingleR)^64^, combined, batch-corrected, and aligned using canonical correlation analysis (Seurat v3, custom R script)^65^. UMAP was then used to project individual cells across a two-dimensional plane based on similarity of gene expression characteristics^63^.

### CD8 IHC and trichrome staining

CD8 IHC was performed on commercially procured SCCHN samples (*n* = 50) by Mosaic Laboratories (Lake Forest, CA) using a monoclonal CD8 (clone C8/144B) antibody (Dako, an Agilent Technologies Co, Santa Clara, CA). Sample details can be found in Supplementary Table 5. A PathAI-developed artificial intelligence (AI)-powered image analysis algorithm (PathAI research platform, Boston, MA) was used to classify the tumor parenchymal and stromal regions and identify and quantify the density (cells/mm^2^) of CD8+ T cells in each compartment^27^.

Collagen was visualized on adjacent sections using a trichrome staining method consisting of Weigert’s hematoxylin, Biebrich scarlet-acid fuchsin, phosphomolybdic-phosphotungstic acid, and aniline blue, as described previously^66^. Collagen staining was assessed by image analysis (Halo software, Indica Labs, Albuquerque, NM). Tumor parenchymal and stromal compartments were identified, and the percent area of collagen (blue) staining relative to the area of the total stromal compartment was calculated.

### Statistical analyses

Associations between EMT signature scores and other TME signature scores were summarized by Pearson’s correlation coefficient (r) and linear regression models for each TCGA tumor type. For the scRNA-seq data, the strength of evidence for differences in median EMT signature scores between stroma fibroblasts and tumor parenchymal cells was assessed by Wilcoxon signed-rank tests. Cox proportional hazards regression models were used to explore the association between EMT and CAF signature scores and OS. Estimated hazard ratios (HRs; high signature score/low signature score) were scaled to compare hazards for scores differing by the interquartile range for each tumor type.

## Supplementary Information

Supplementary Information 1.

Supplementary Information 2.

Supplementary Information 3.

Supplementary Information 4.

Supplementary Information 5.

Supplementary Information 6.

## Data Availability

The single cell RNA-seq data generated in this study have been deposited in NCBI’s Gene Expression Omnibus (GEO) and are accessible through GEO Series accession number GSE213912. The mouse PDX model RNA-seq count tables and metadata generated in this study have been deposited in GEO and are accessible through GEO Series accession number GSE215307. The raw FASTQ files may be obtained from a repository at the Wasabi cloud platform upon approval by the Dana Farber Cancer Institute Center for Derived Patient Models. More information on Bristol Myers Squibb’s data sharing policy can be found here: https://www.bms.com/researchers-and-partners/clinical-trials-and-research/disclosure-commitment.html. Any additional data not included in the manuscript or supplementary files that support the findings of this study are available from the corresponding author N.K.
