# Supplementary material for: Cancer-associated fibroblasts are the main contributors to epithelial-to-mesenchymal signatures in the tumor microenvironment
Source: Sci Rep. 2023 Feb 21;13:3051. doi: 10.1038/s41598-023-28480-9 (PMC9944255; doi:10.1038/s41598-023-28480-9)
Supplement: Supplementary file 1 — Supplementary Information 1. [file 41598_2023_28480_MOESM1_ESM.docx]

## **Supplementary Information**

## **Cancer-Associated Fibroblasts are the Main Contributors to Epithelial-to-Mesenchymal Signatures in the Tumor Microenvironment**

Peter M. Szabo, Amir Vajdi, Namit Kumar, Michael Y. Tolstorukov, Benjamin J. Chen, Robin Edwards, Keith L. Ligon, Scott D. Chasalow, Kin-Hoe Chow, Aniket Shetty, Mohan Bolisetty, James L. Holloway, Ryan Golhar, Brian A. Kidd, Philip Ansumana Hull, Jeff Houser, Logan Vlach, Nathan O. Siemers, Saurabh Saha

## **Figures**

### **Supplementary Fig. 1:** Correlation between EMT-related and stromal signature scores by tumor type in cancer datasets from TCGA. Pink plotting symbols represent tumor tissue. Aqua plotting symbols represent samples collected from non-involved healthy tissue of patients with cancer in TCGA.

a


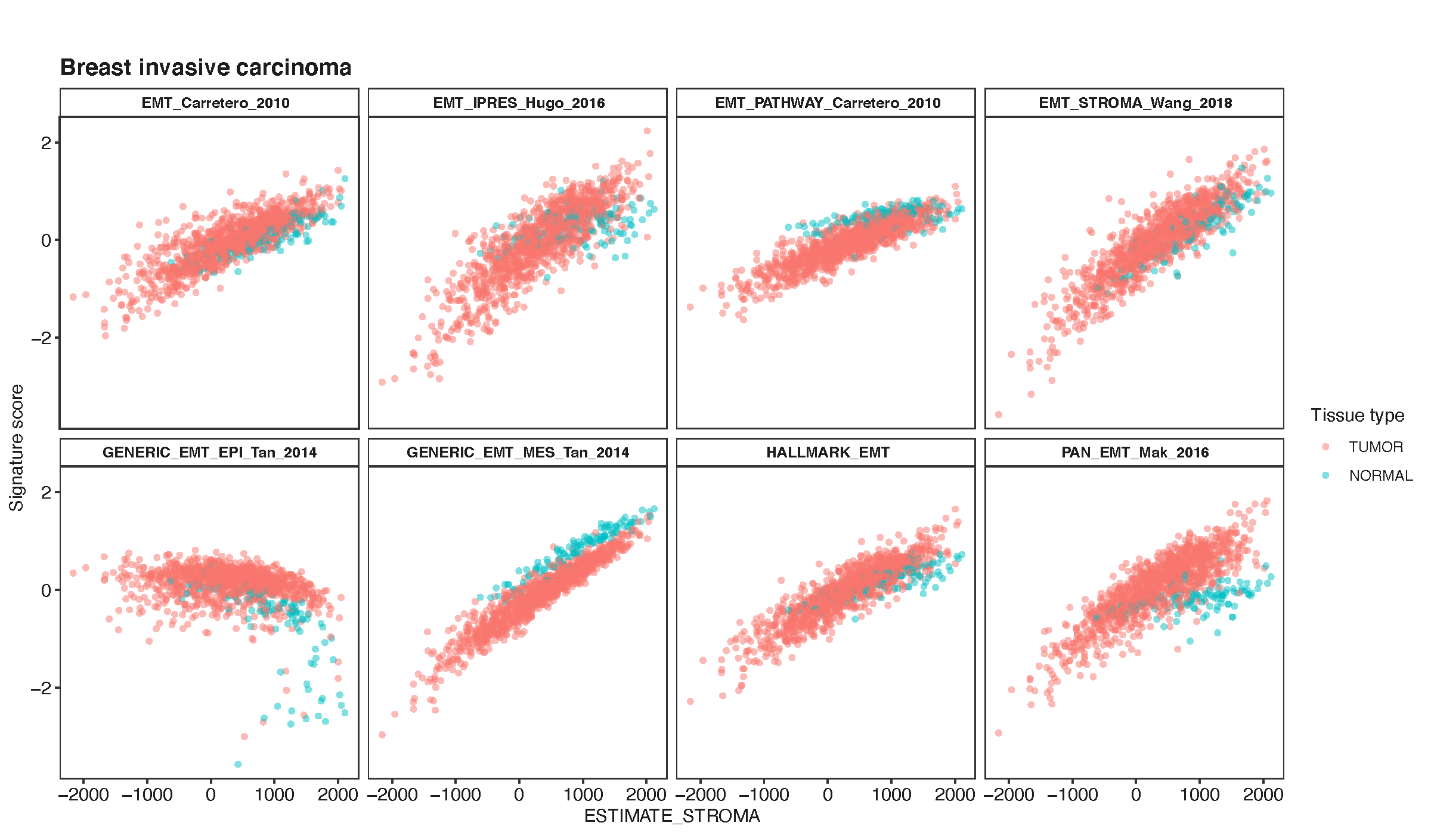


b


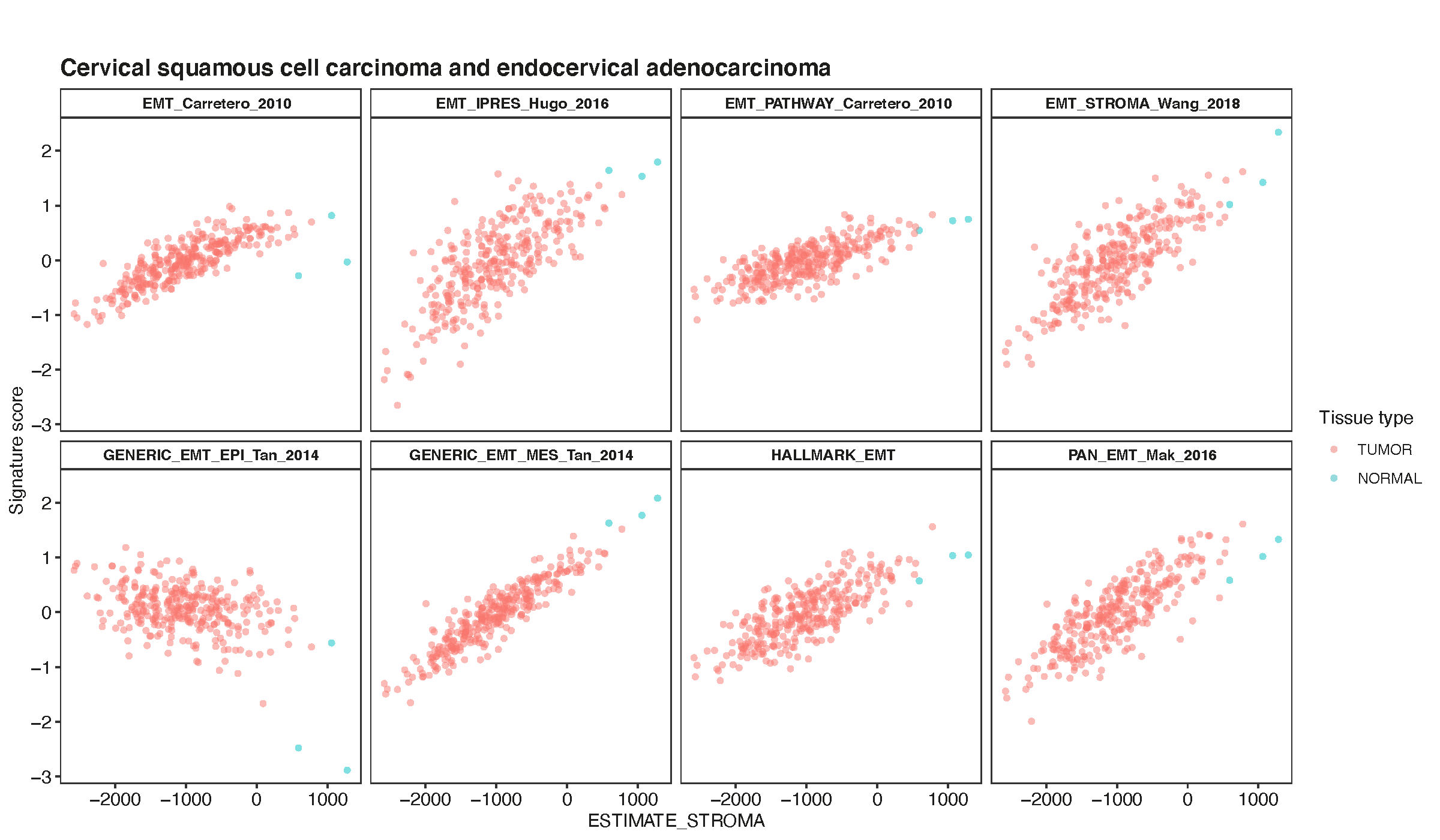


c


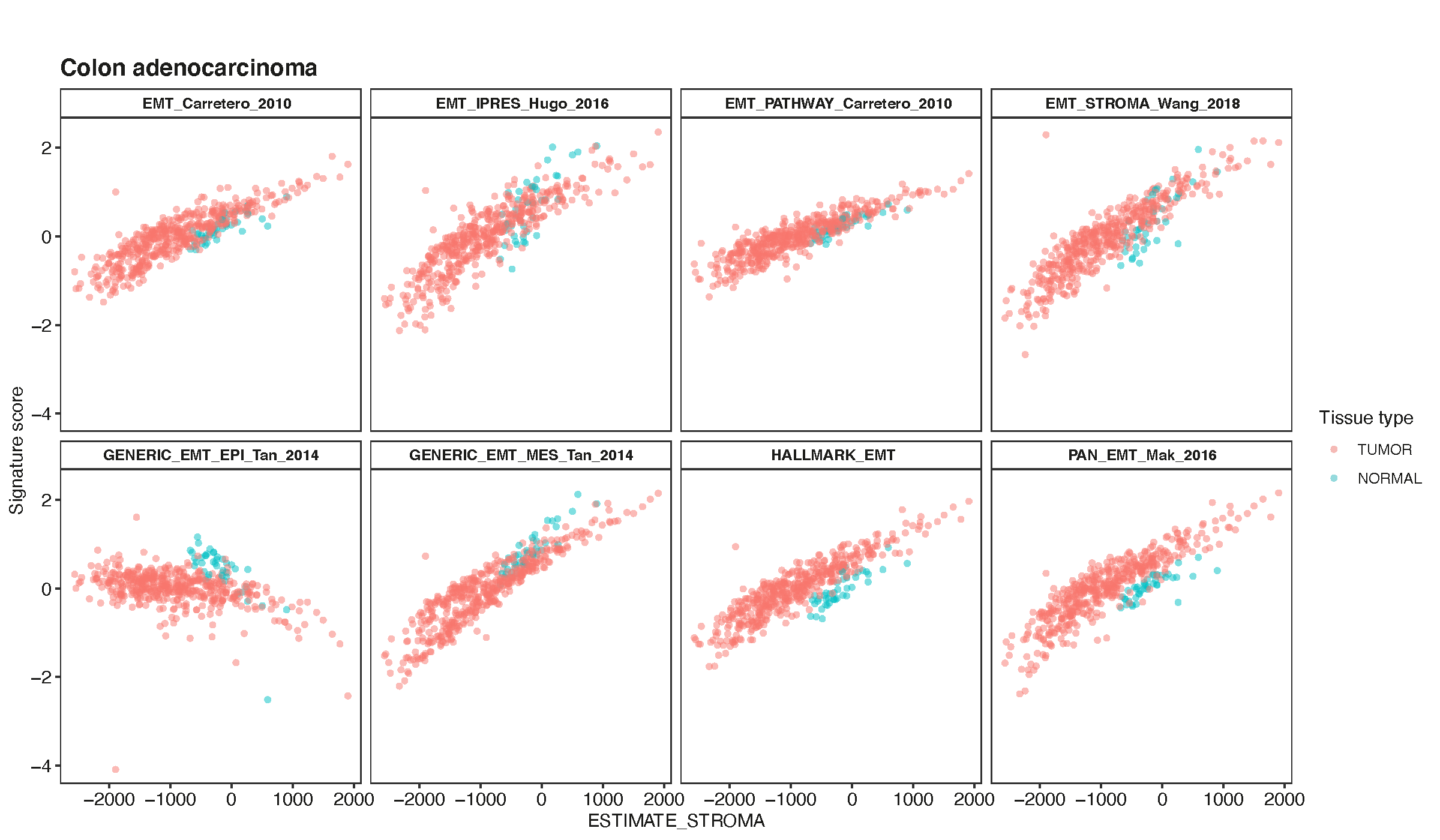


d


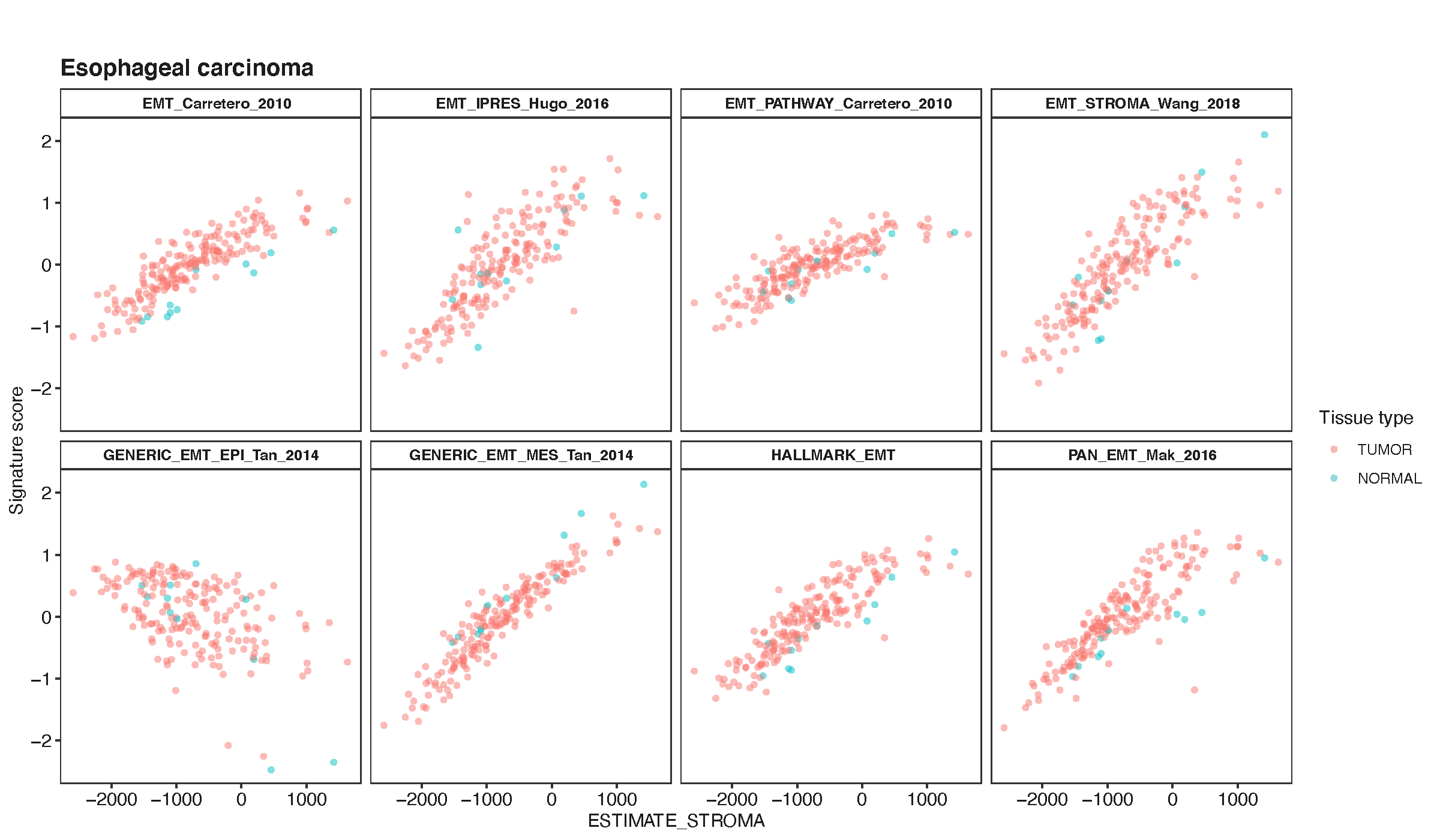


e


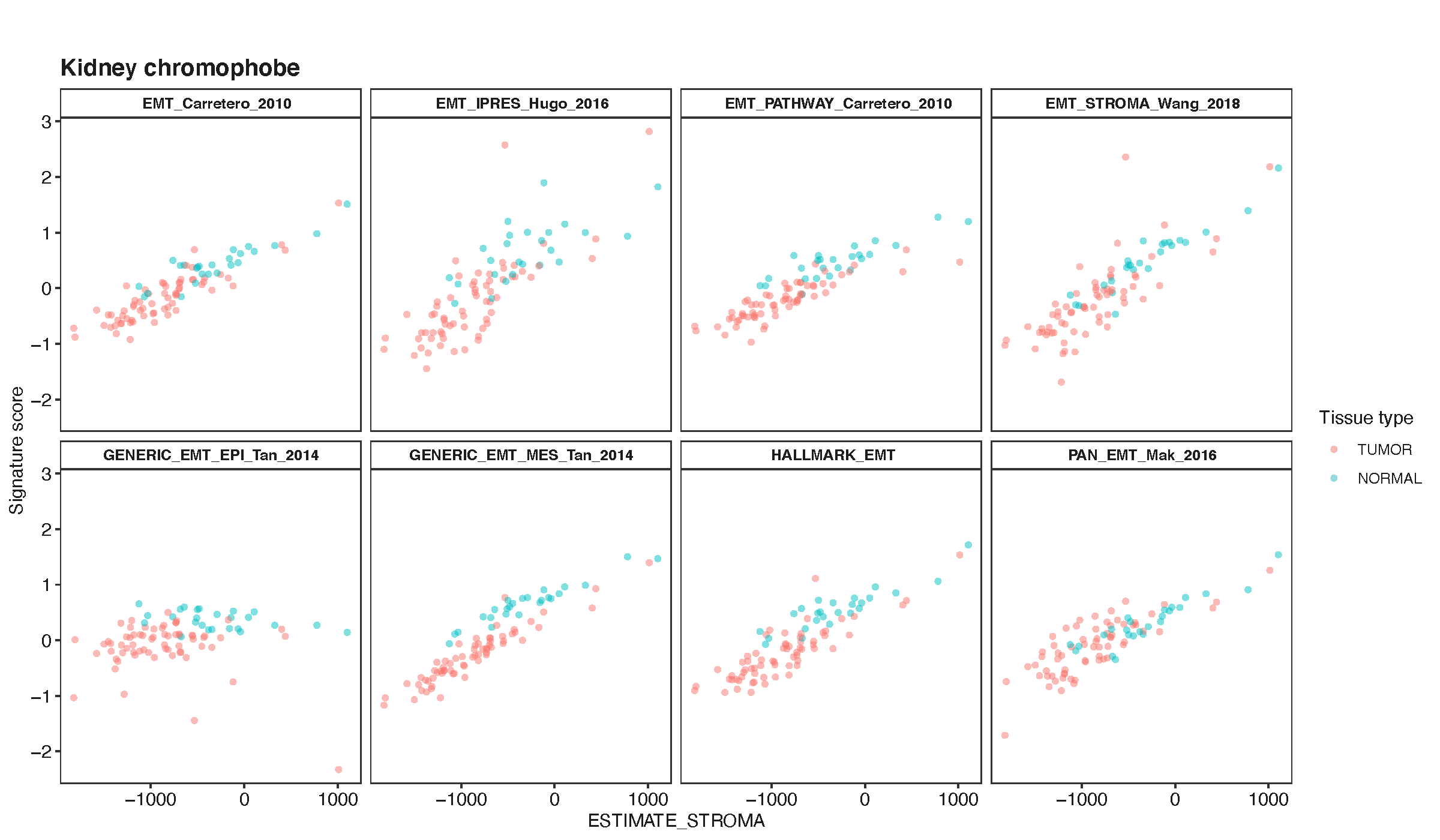


f


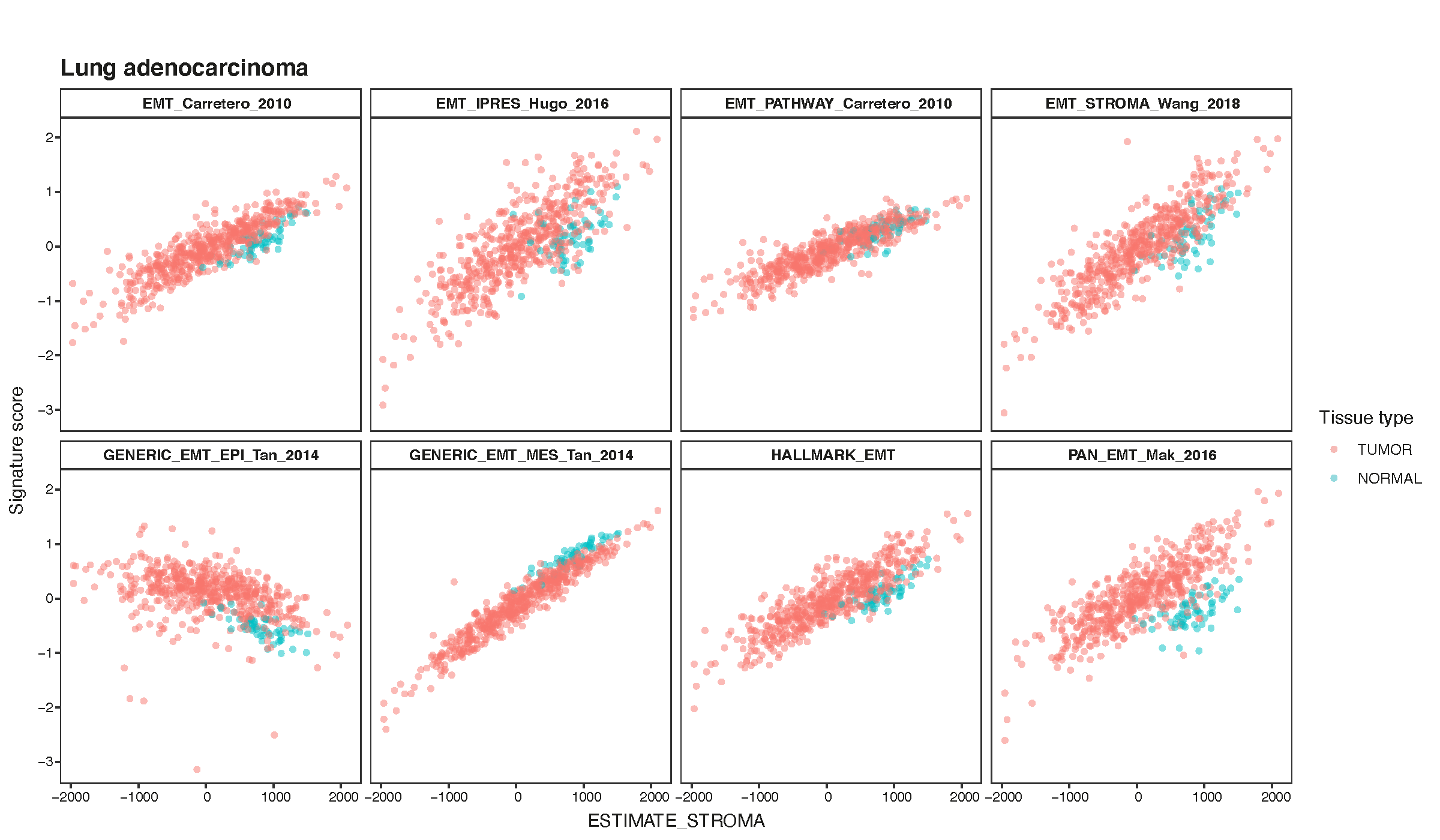


g


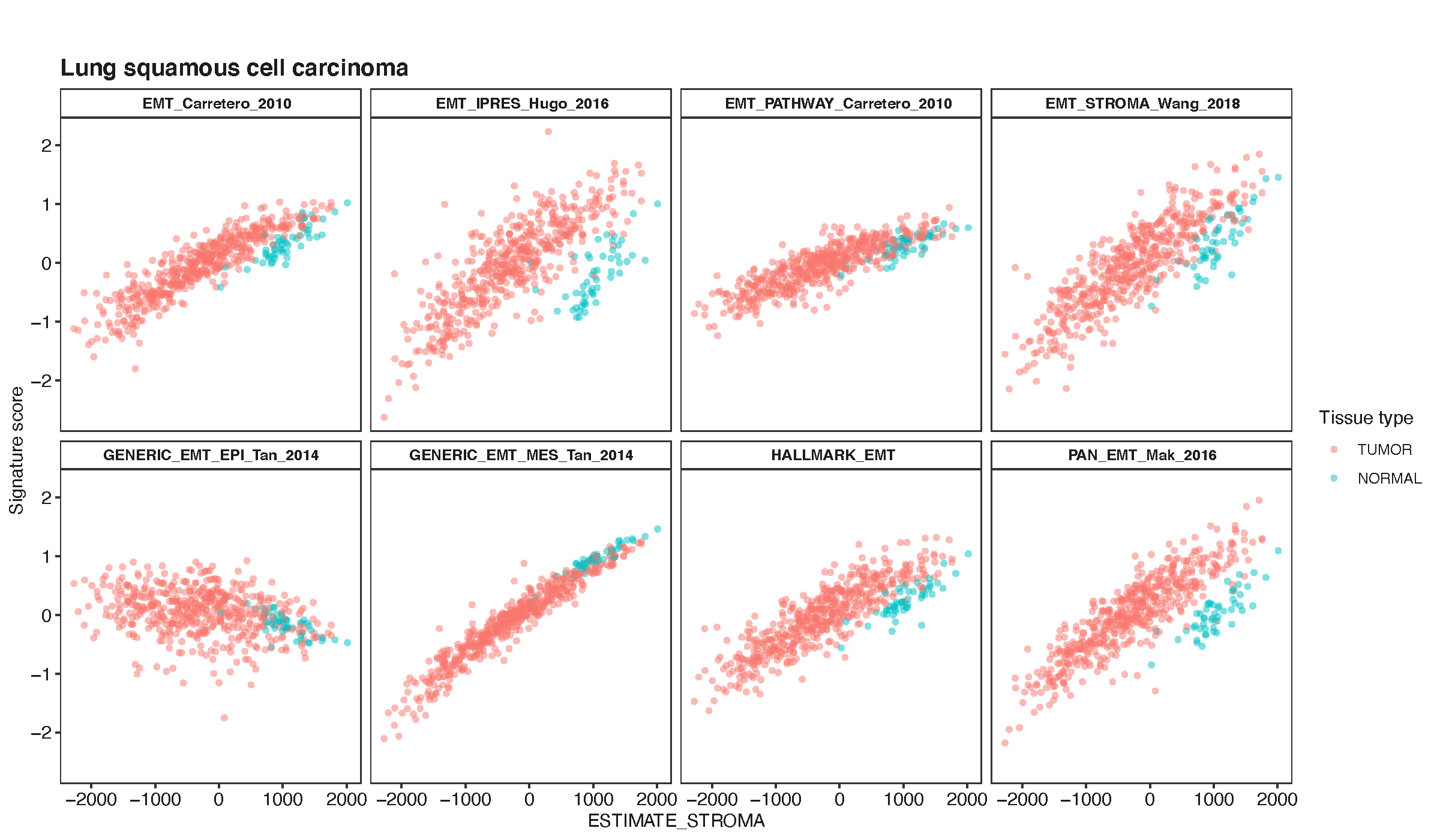


h


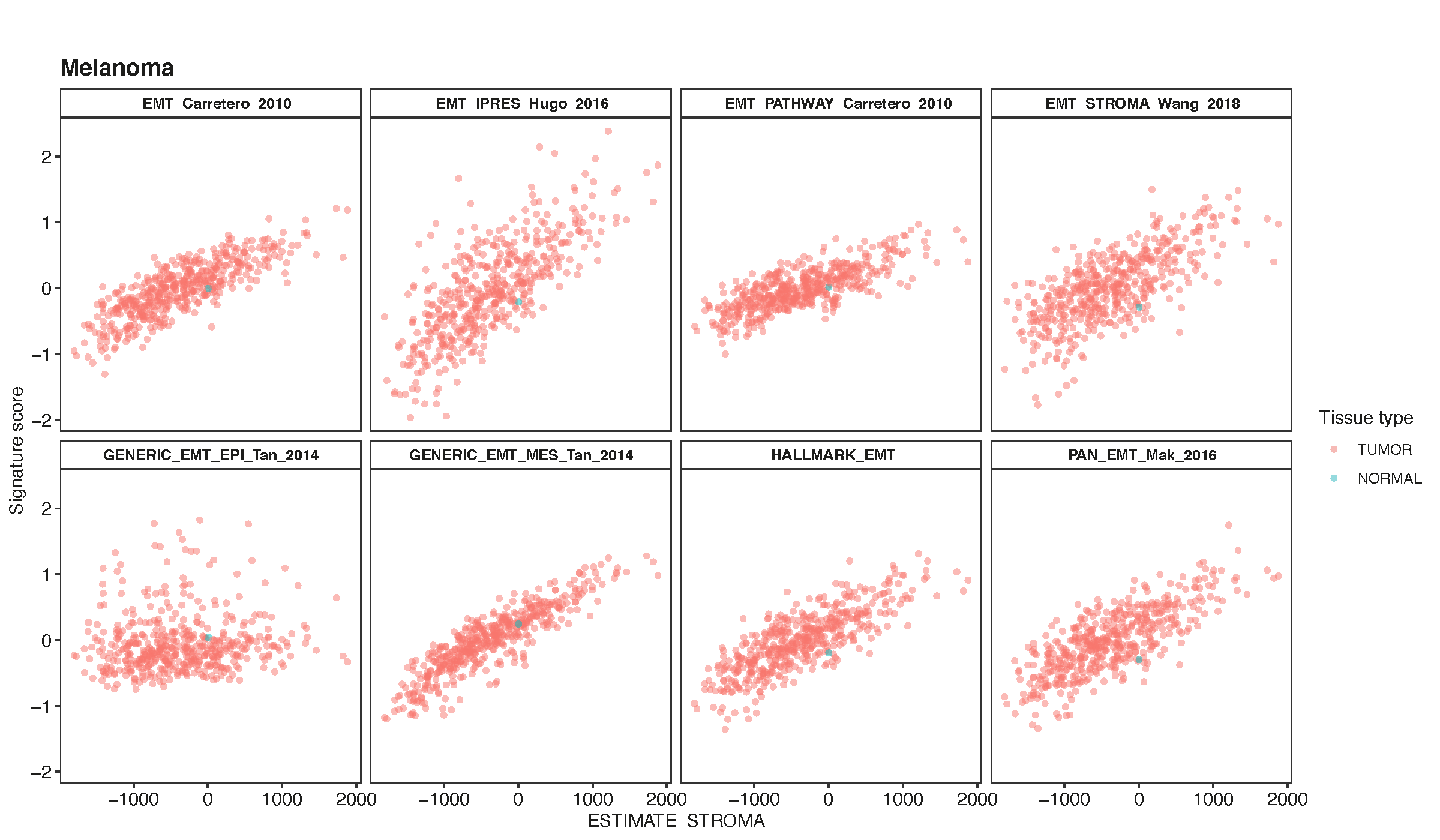


i


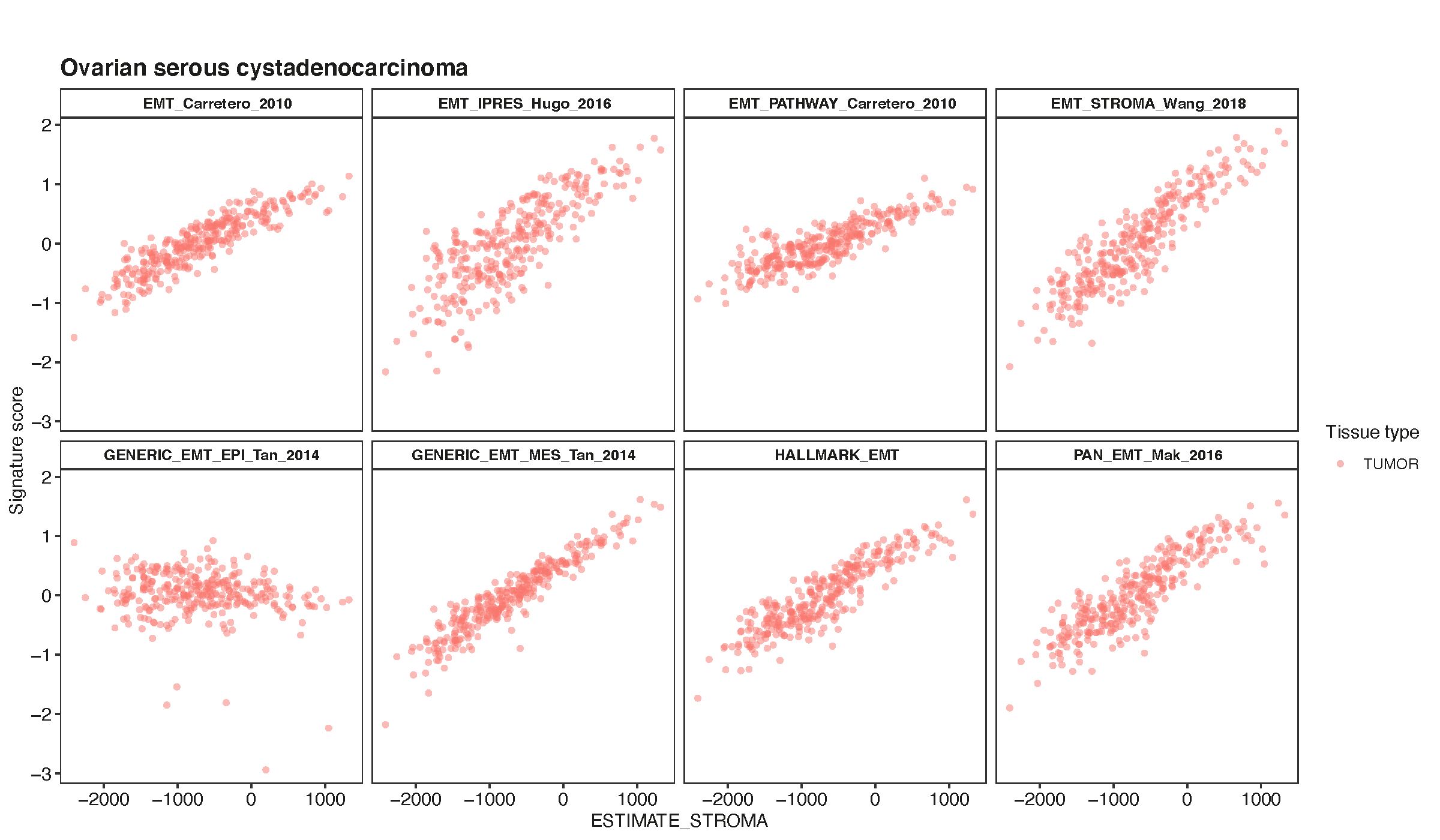


j


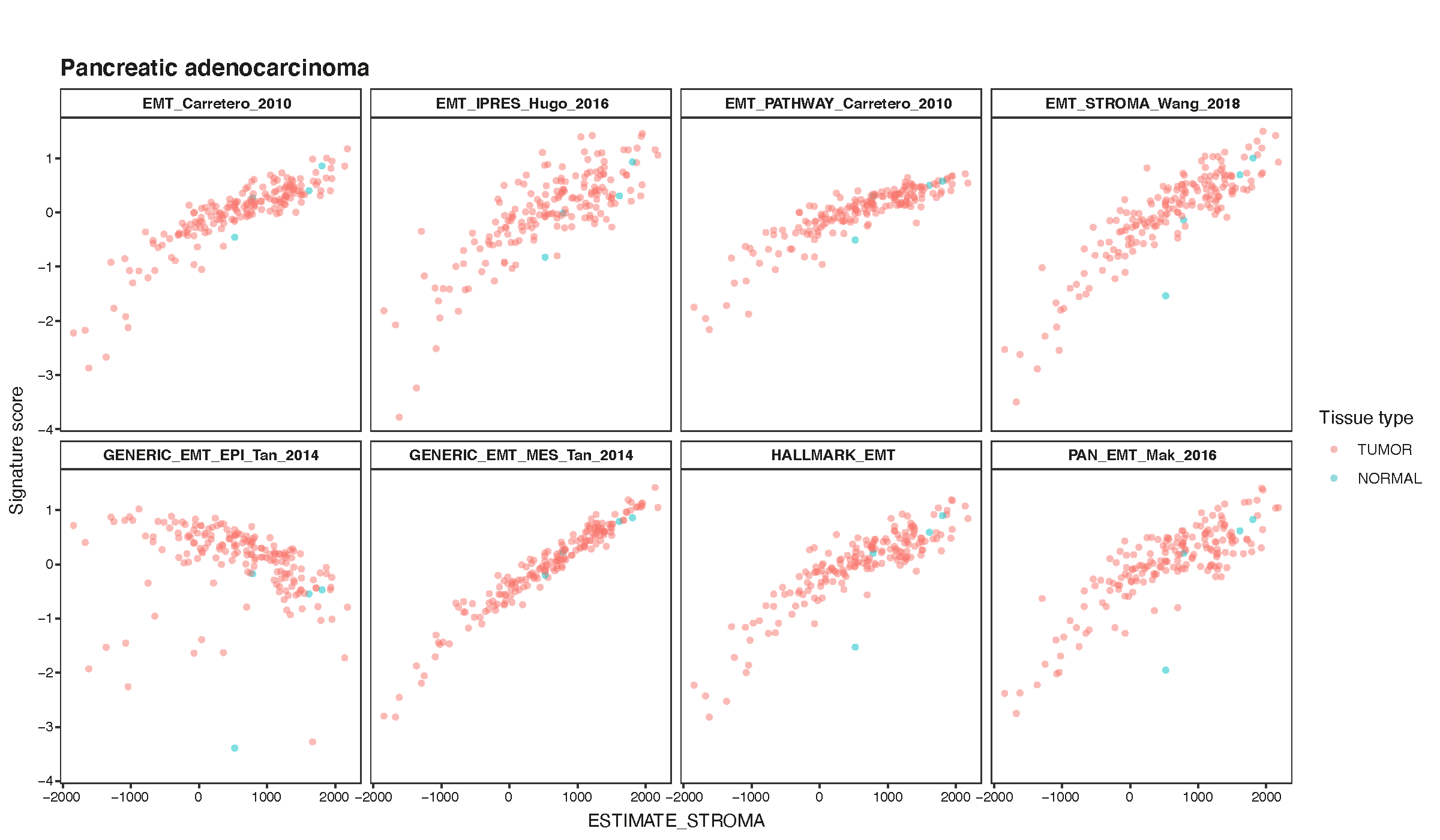


k


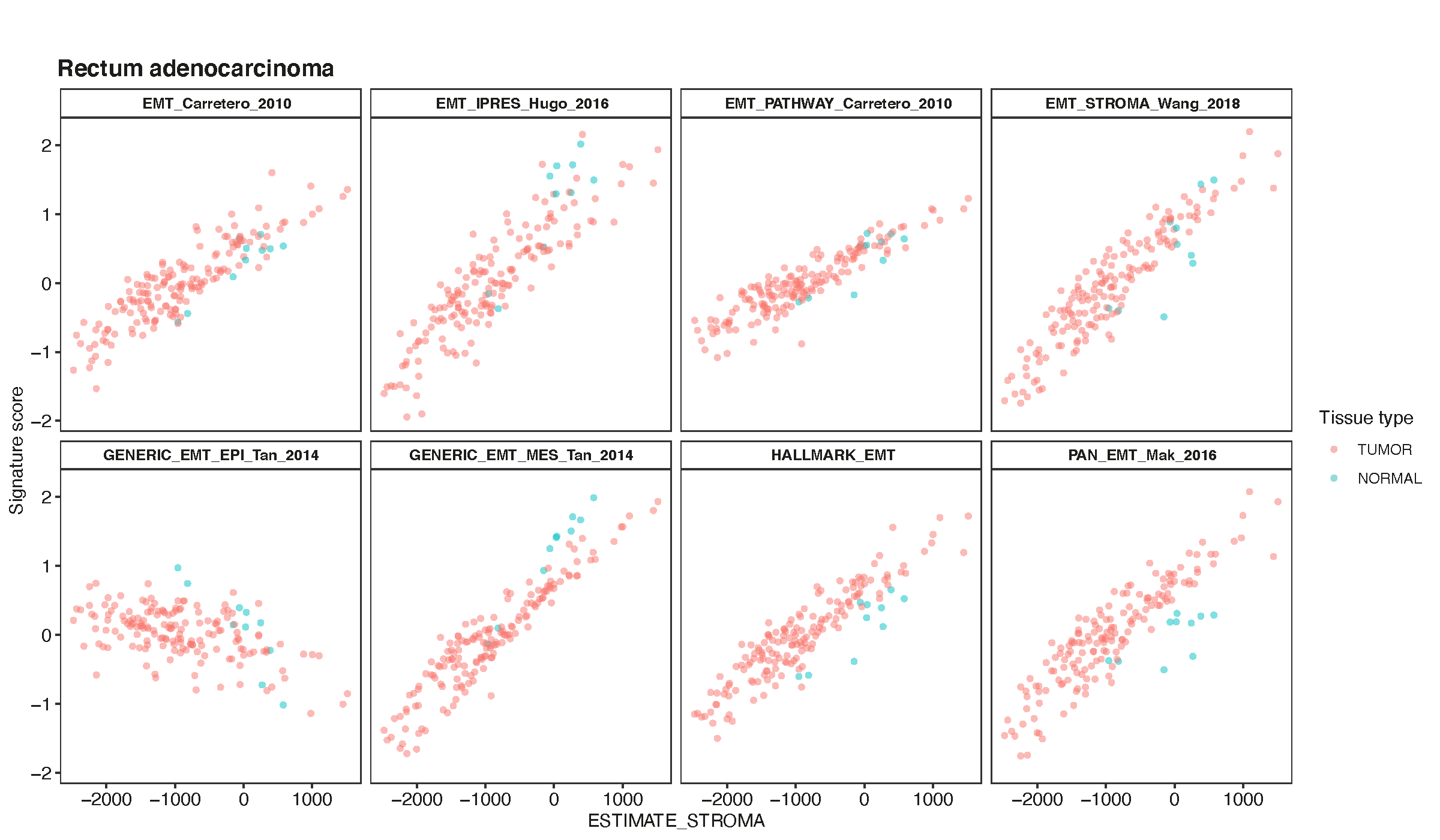


l


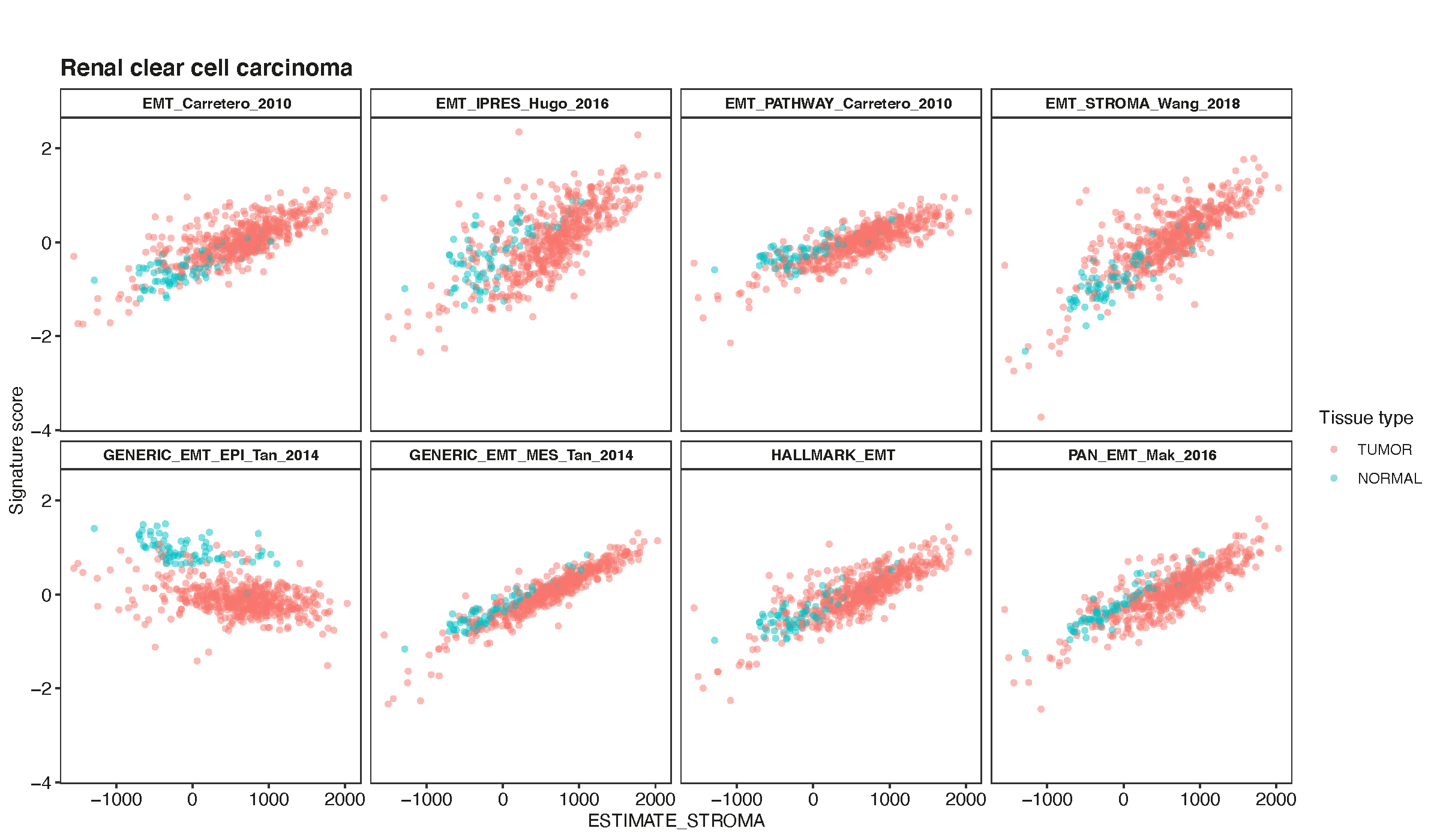


m


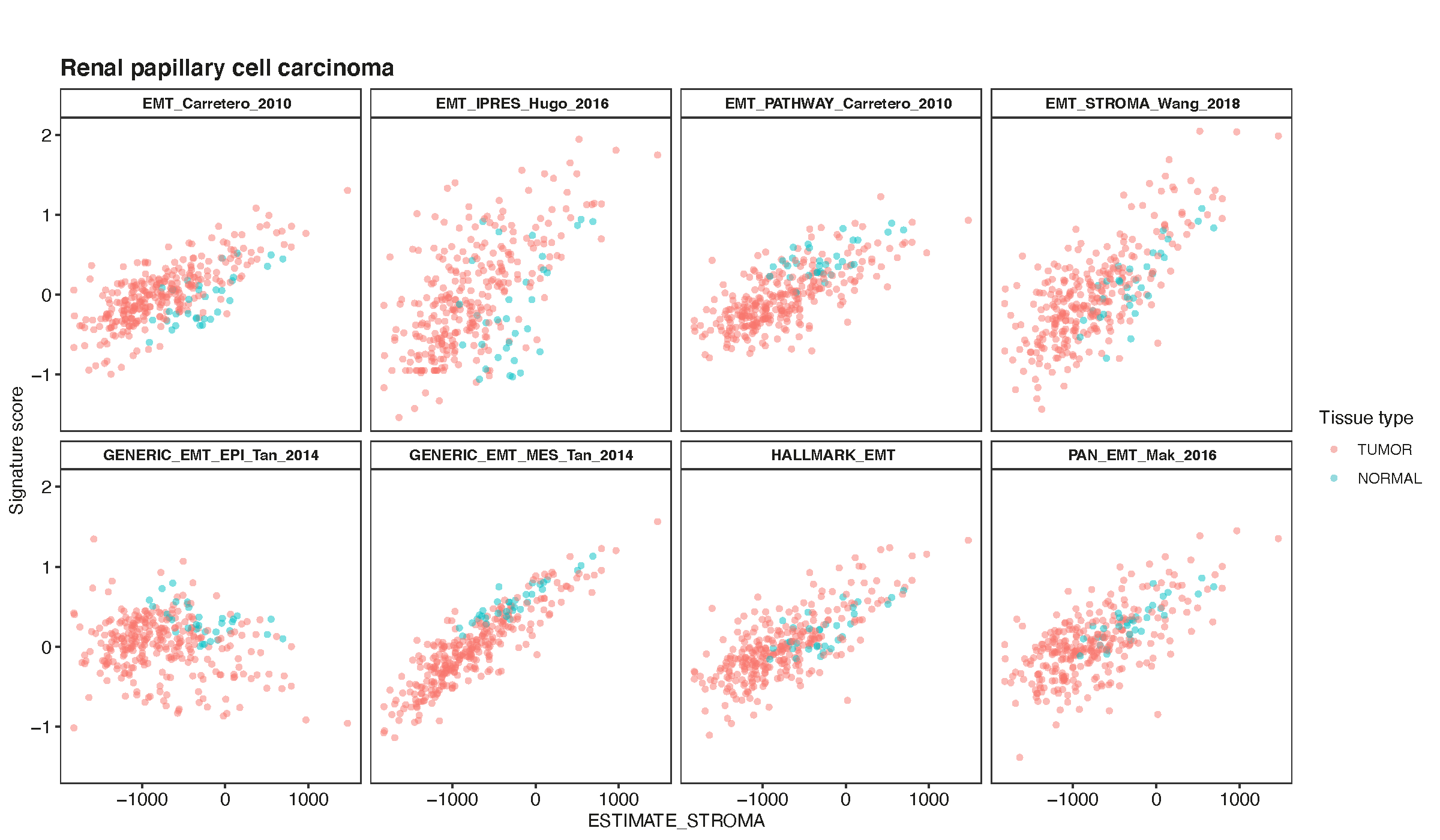


n


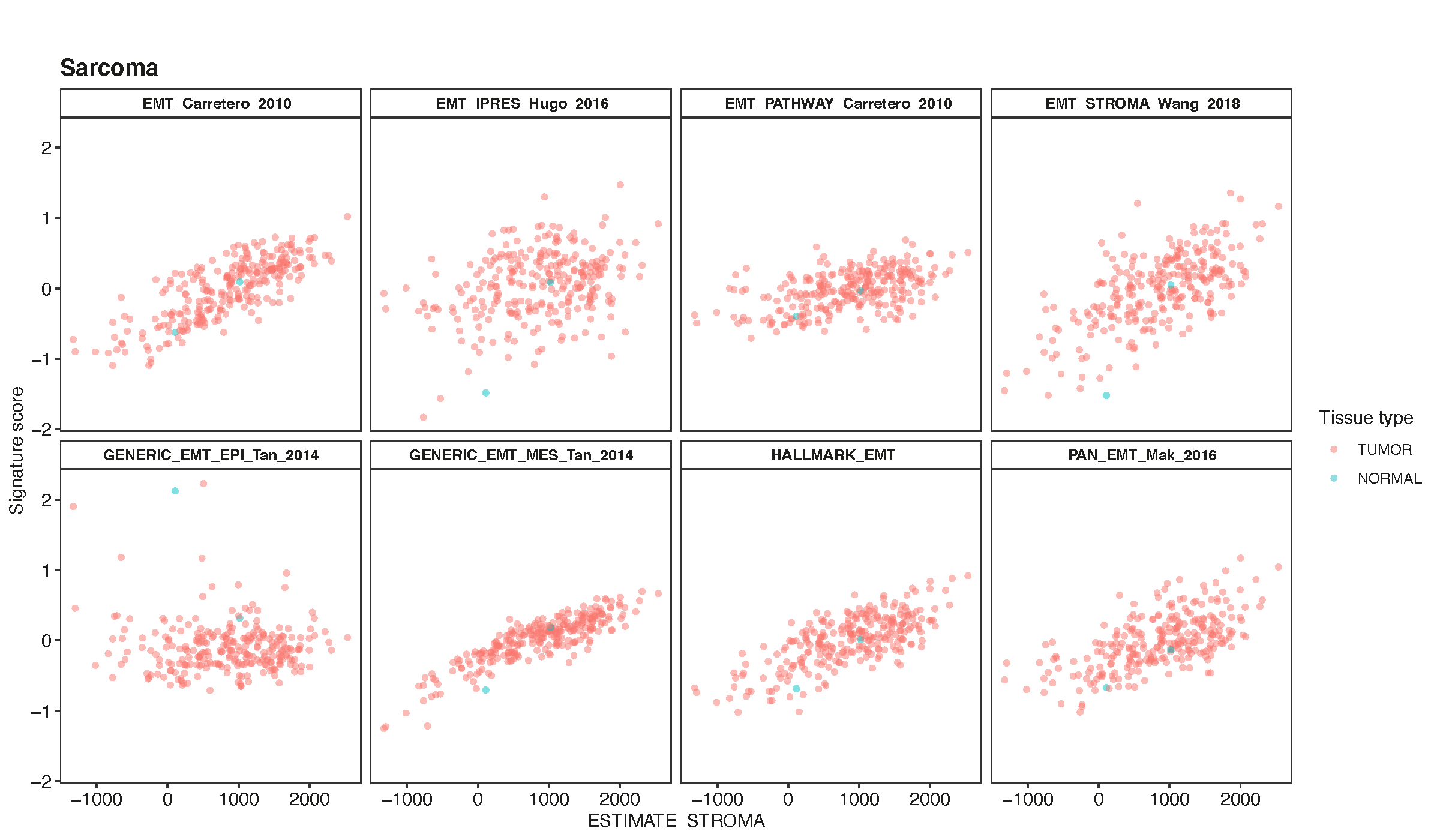


o


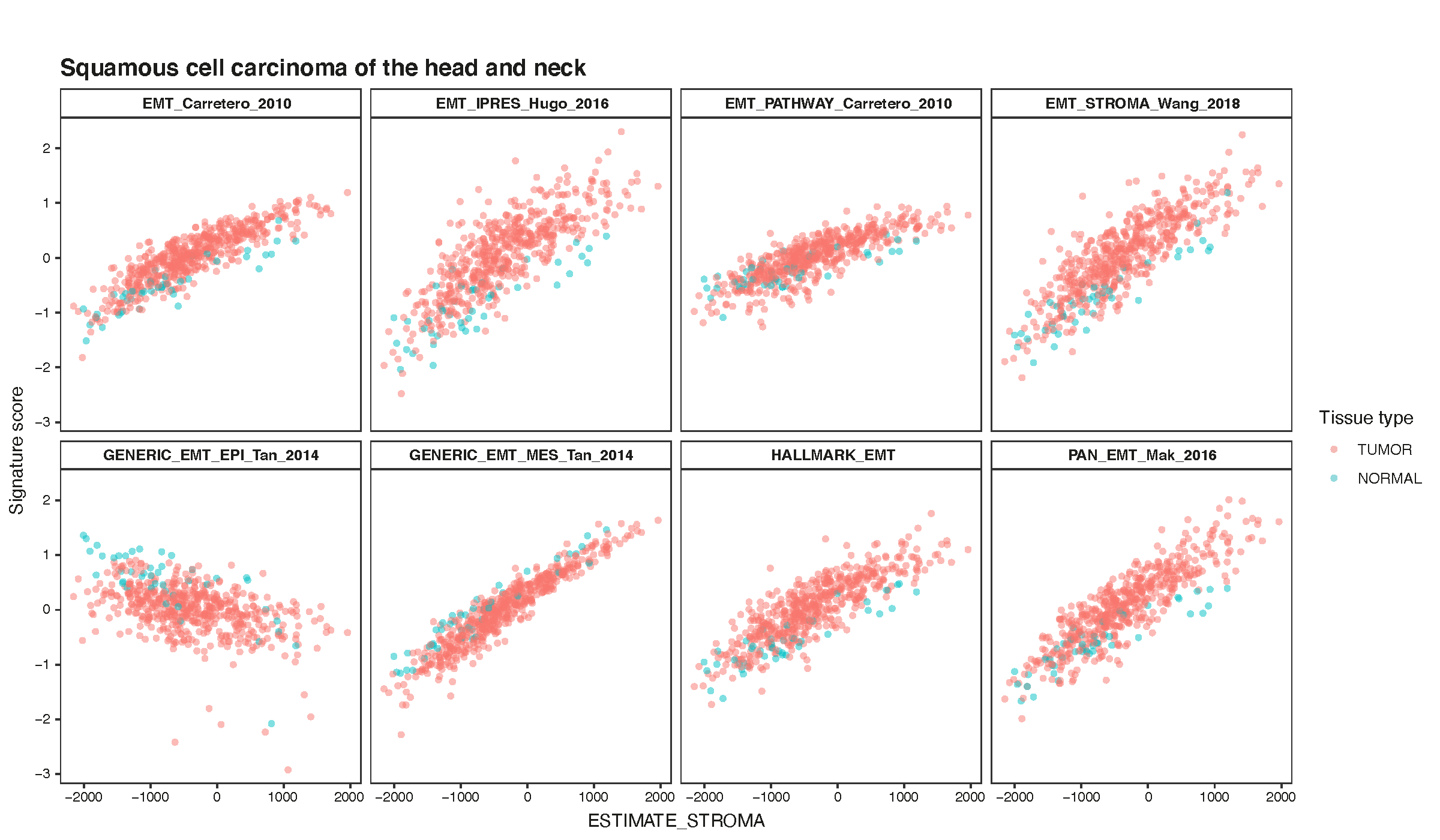


p


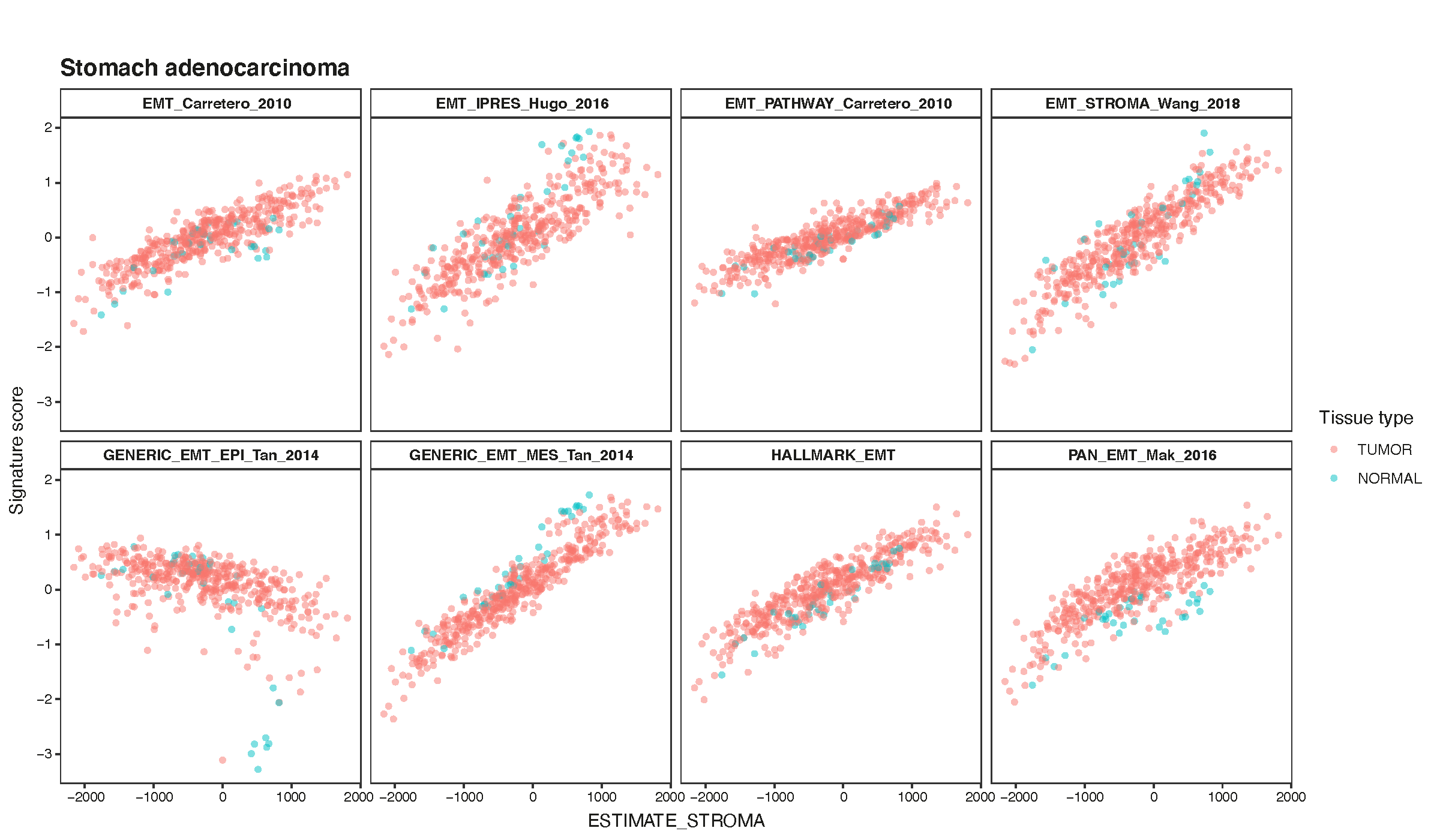


q


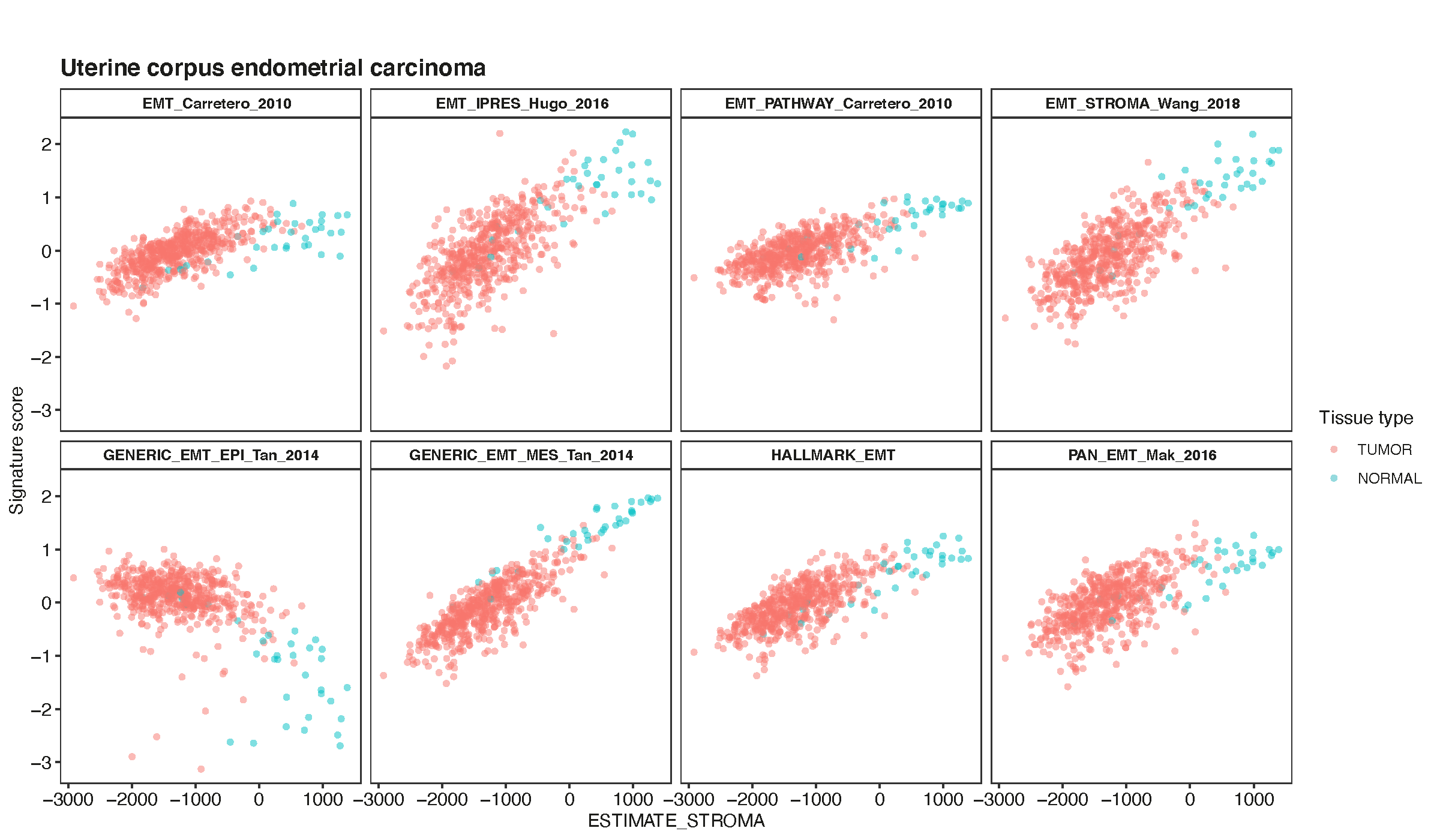


### **Supplementary Fig. 2:** Expression of EMT-related genes in mouse (stromal) and human (tumor parenchymal) tissues using PDX samples. Horizontal reference line indicates proportion of reads from stroma = 0.5. Boxes extend from the first to third quartiles, the middle line shows the median, and the whiskers extend to the most extreme data point that is no more than 1.5 times the interquartile range from the box, open circles show individual values that are more than 1.5 times the interquartile range from the box. **a** EMT_Carretero_2010. **b** IPRES_Hugo_2016. **c** PATHWAY_Carretero_2010. **d** EMT_STROMA_Wang_2018. **e** ESTIMATE_STROMA. **f** GENERIC_EMT_EPI_Tan_2014. **g** GENERIC_EMT_MES_Tan_2014. **h** HALLMARK_EMT. **i** PAN_EMT_Mak_2016.

a


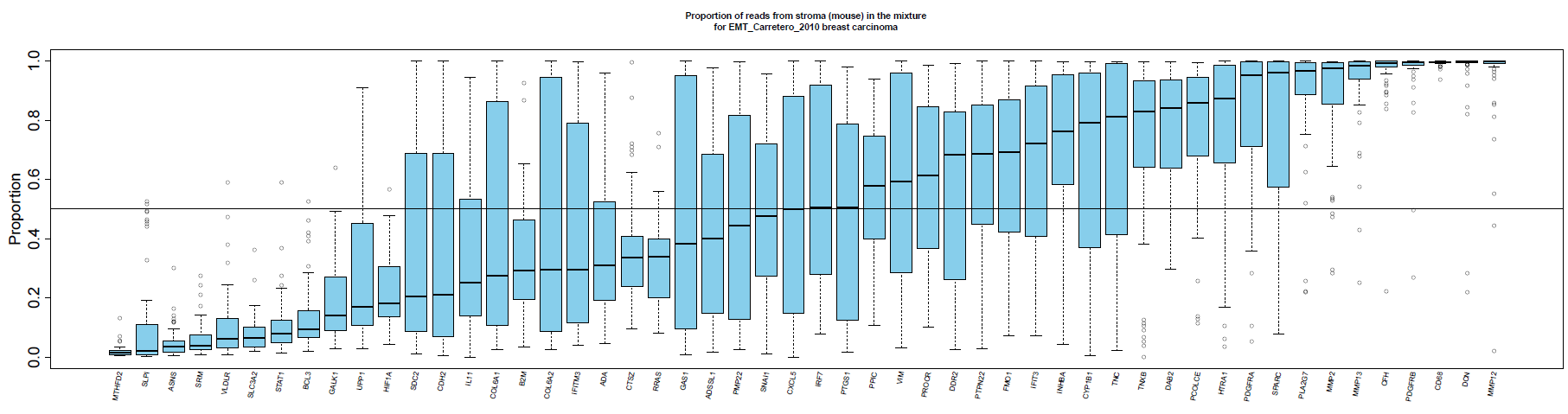


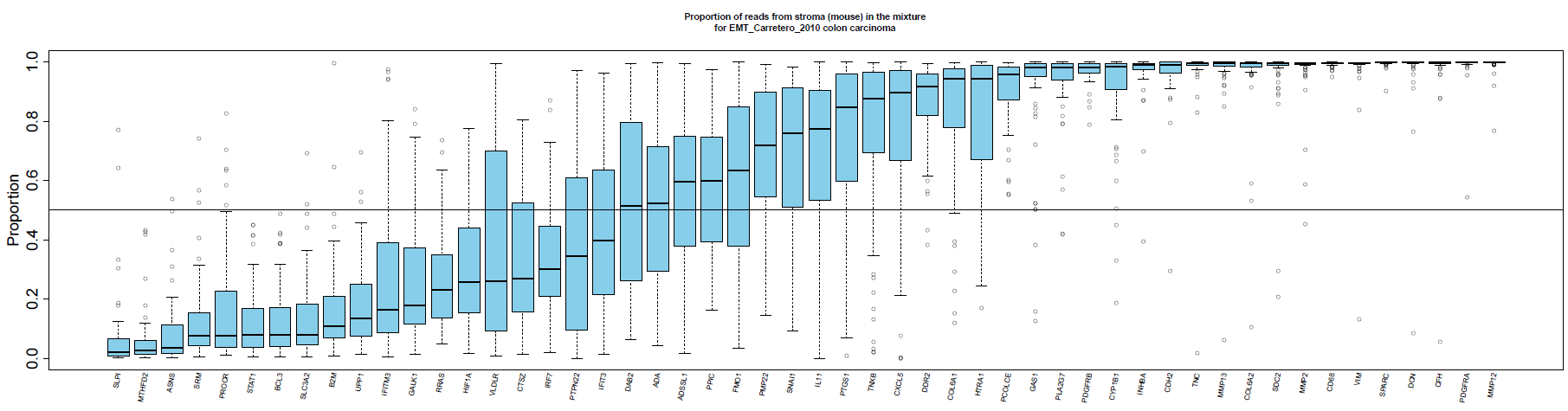

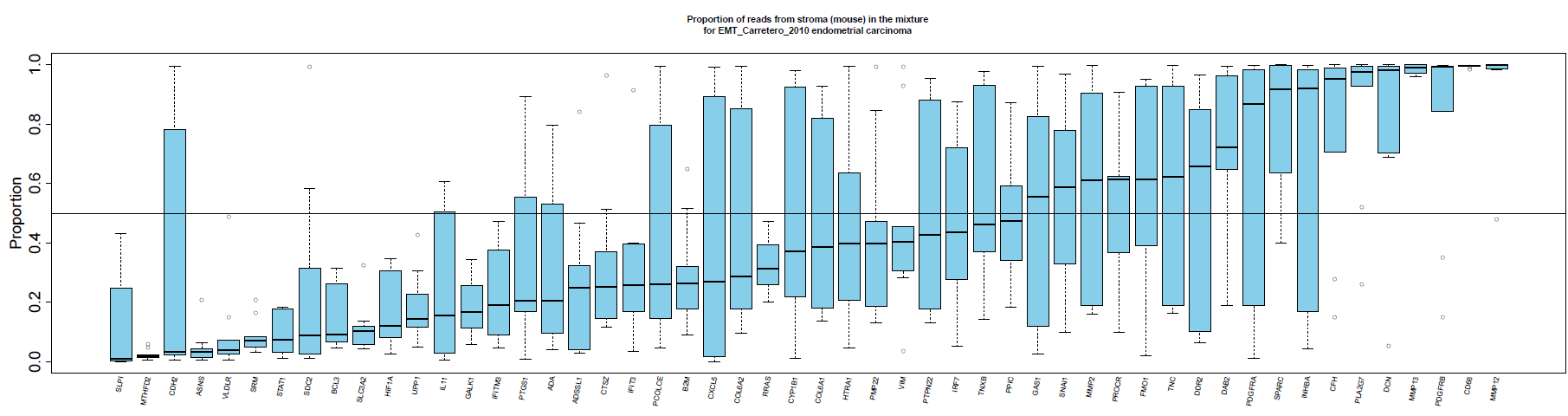

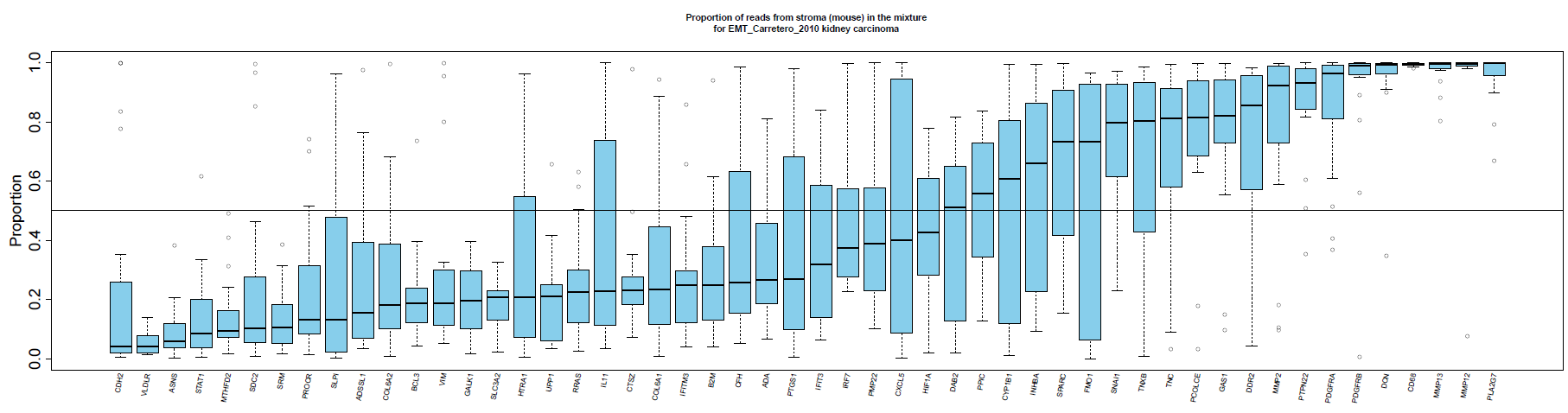

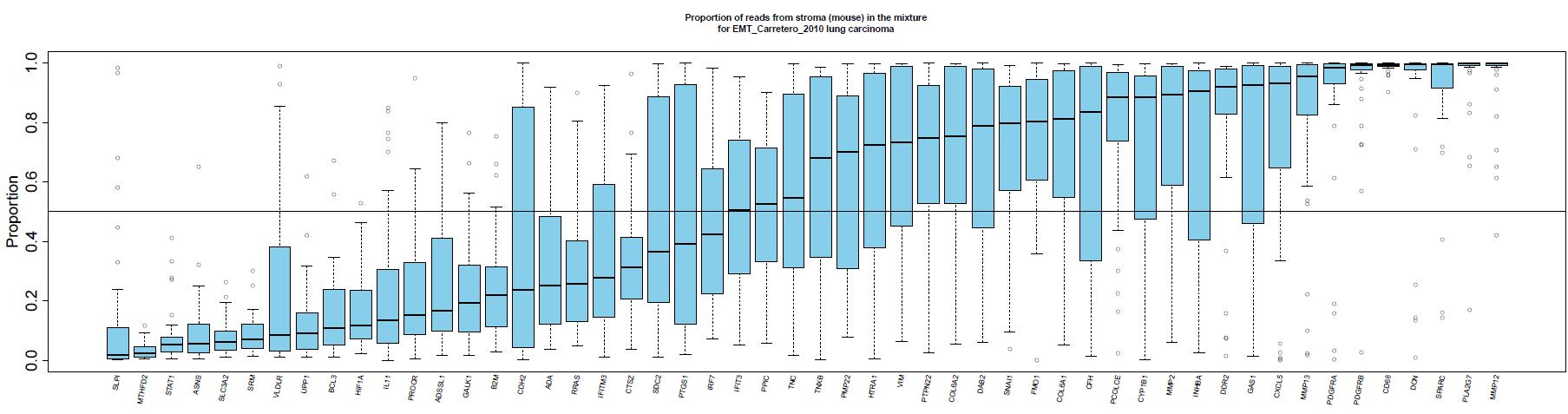

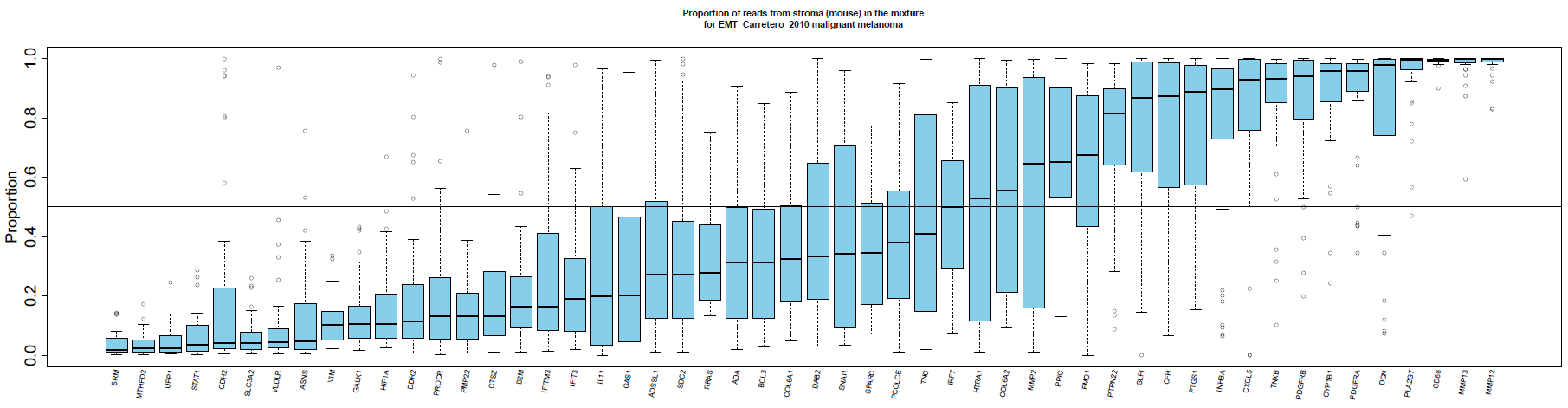

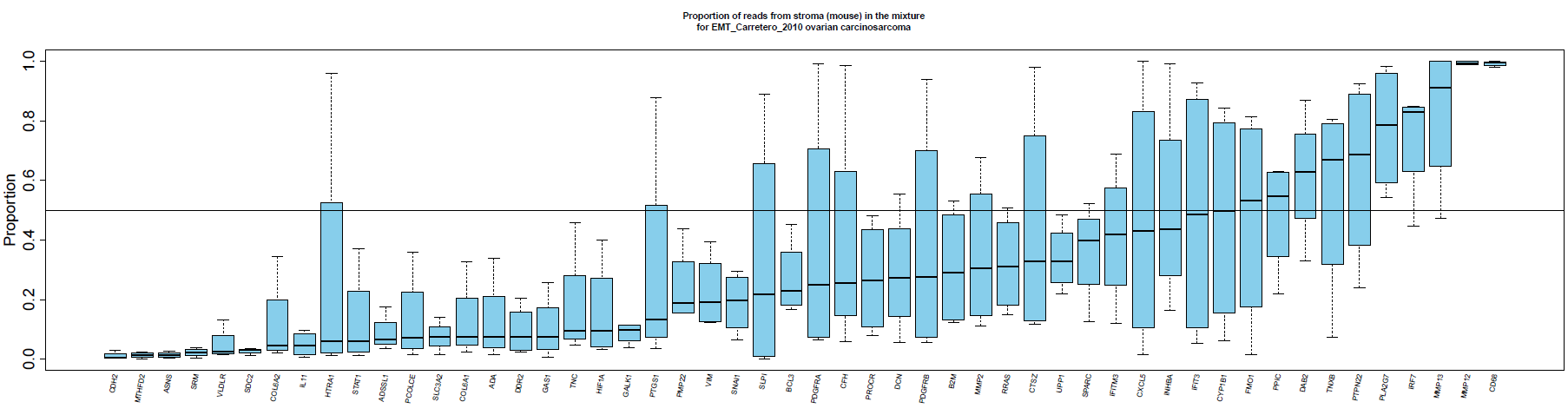

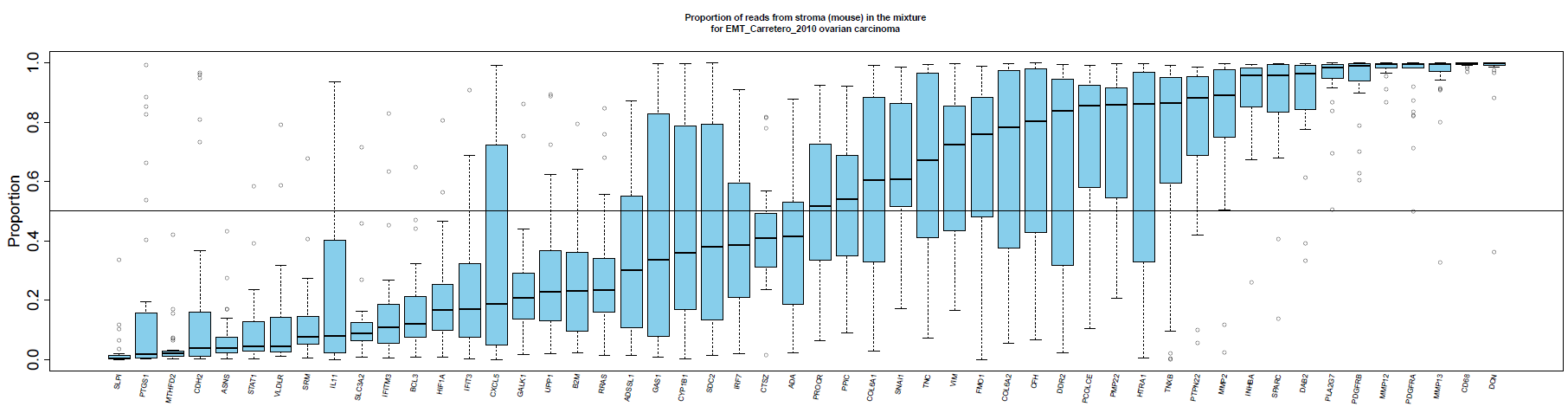

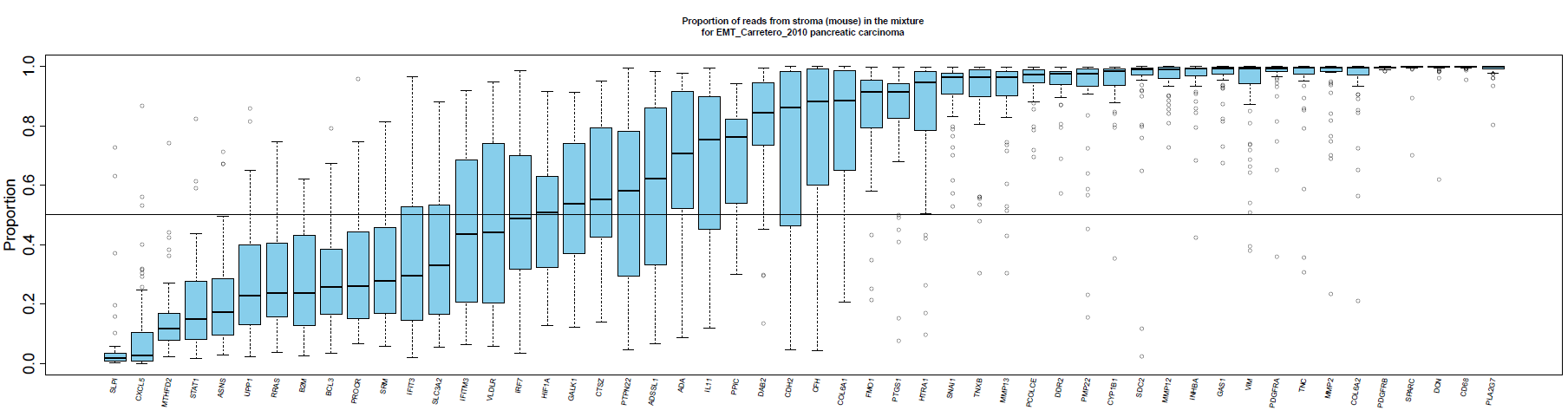

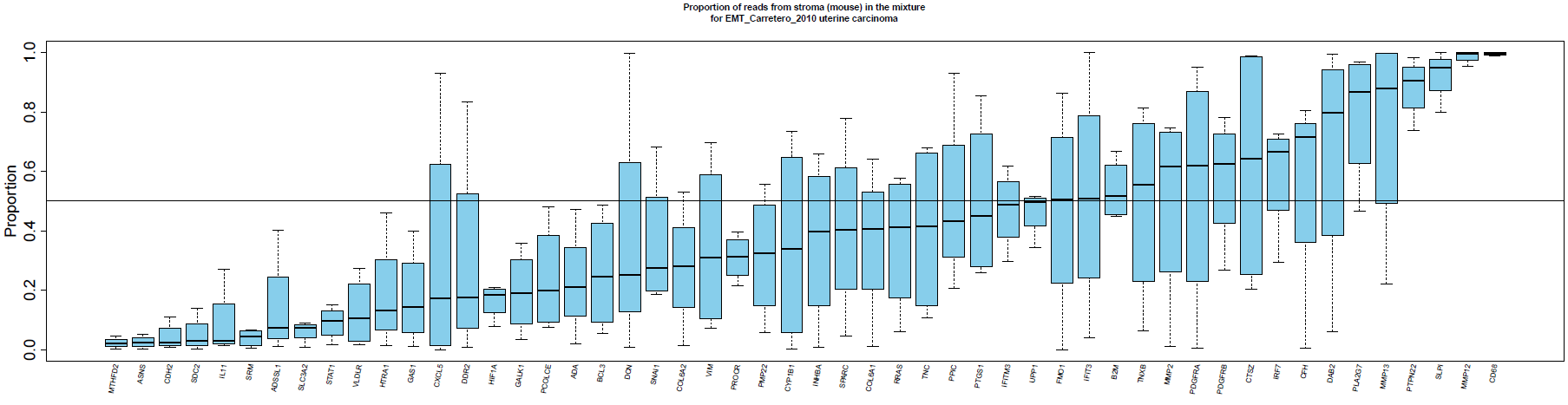


b


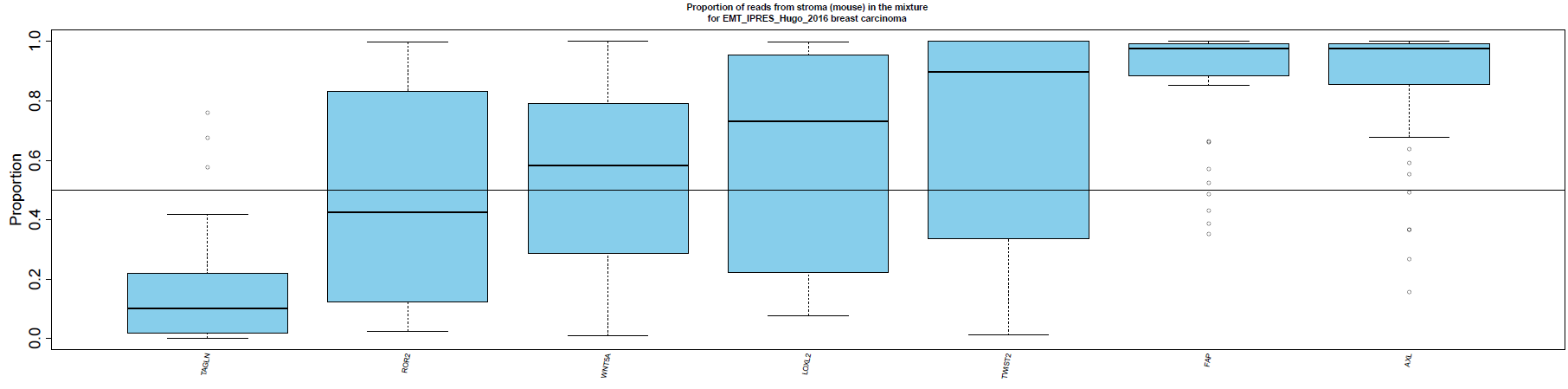

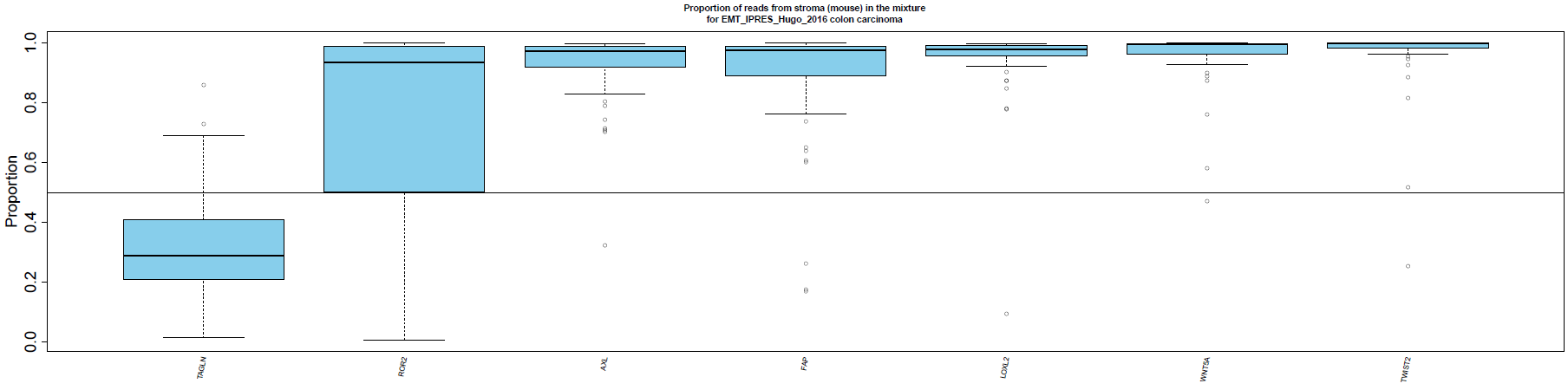

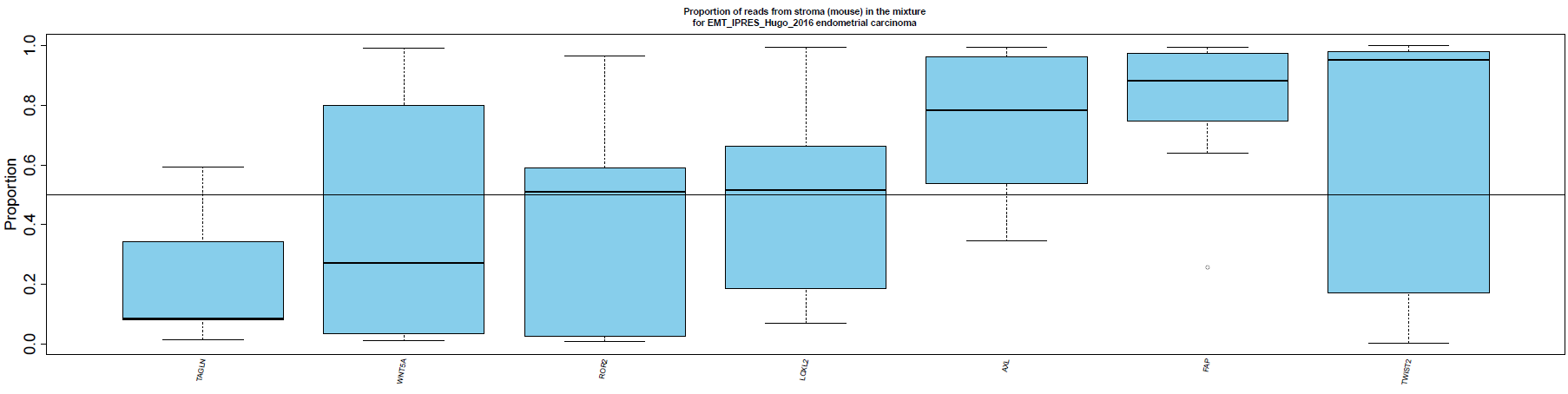

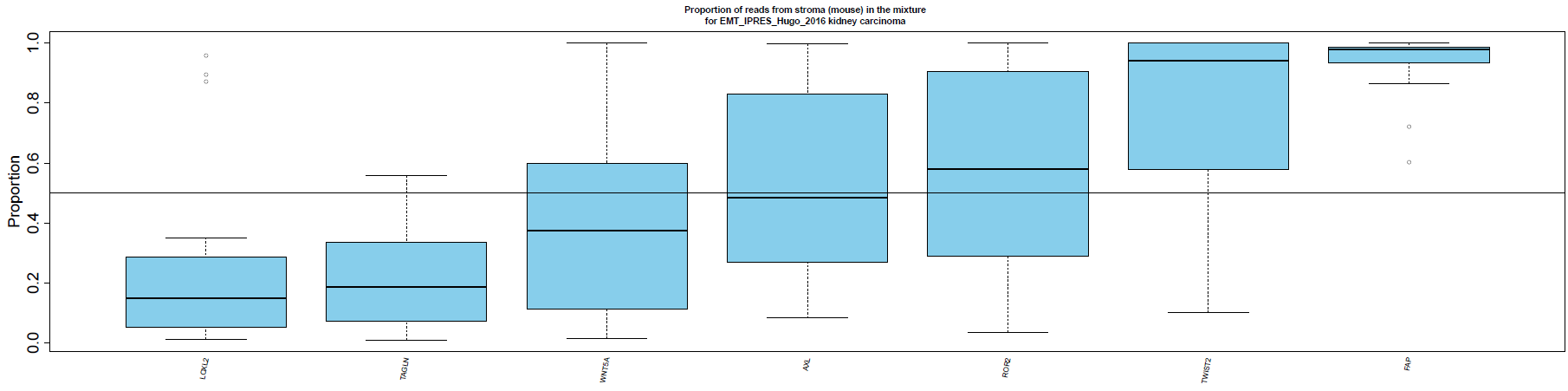

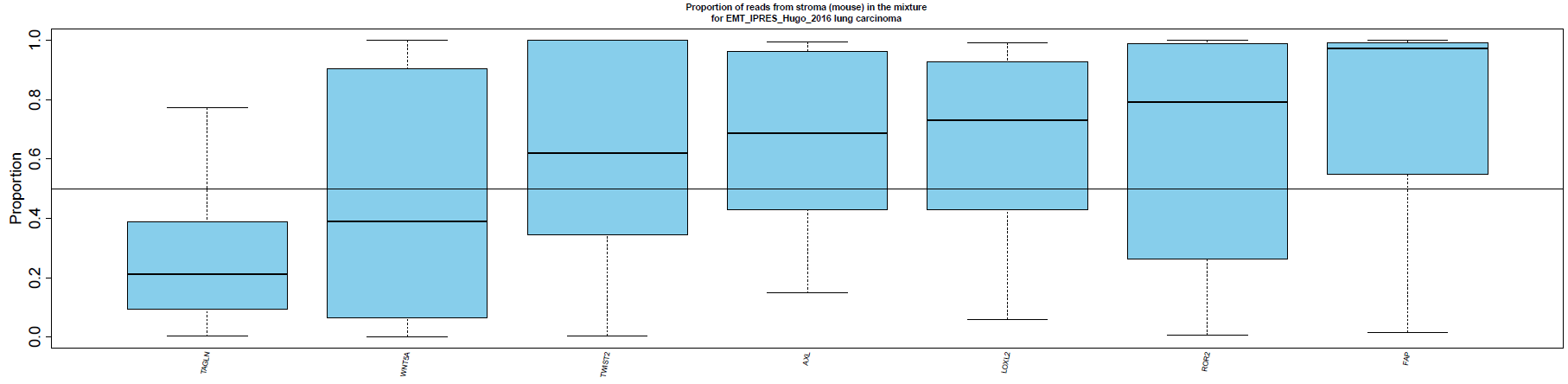

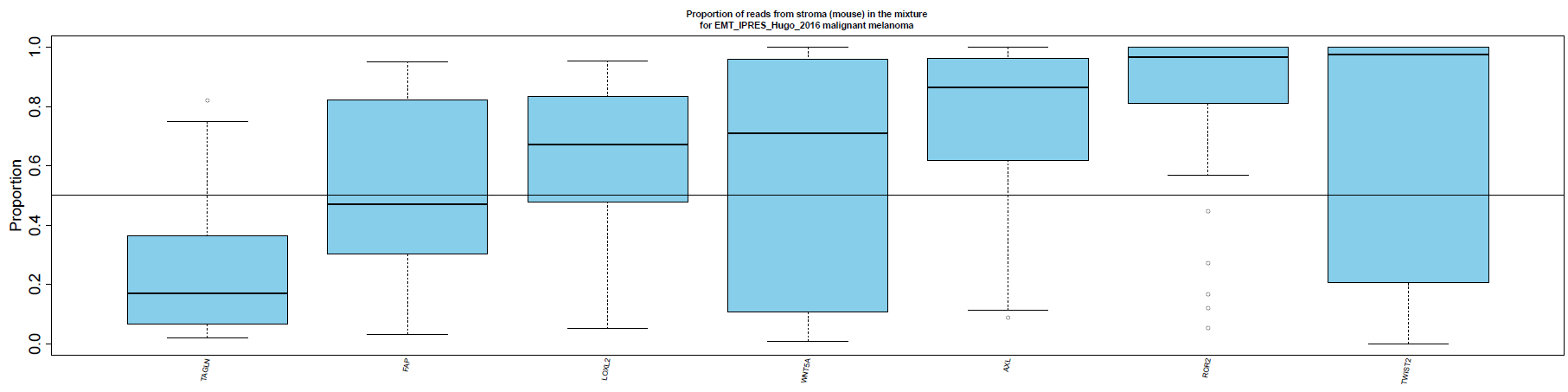

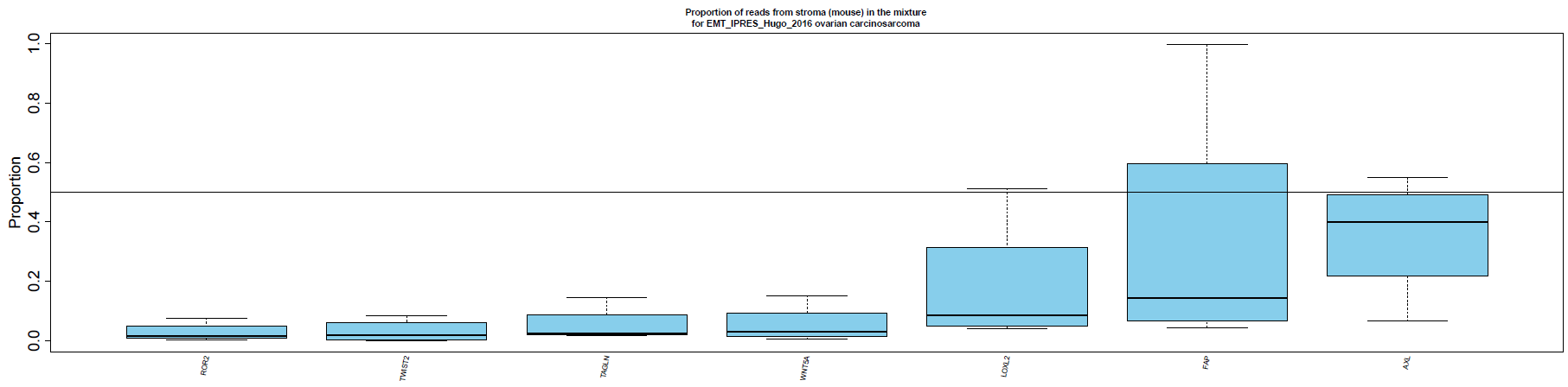

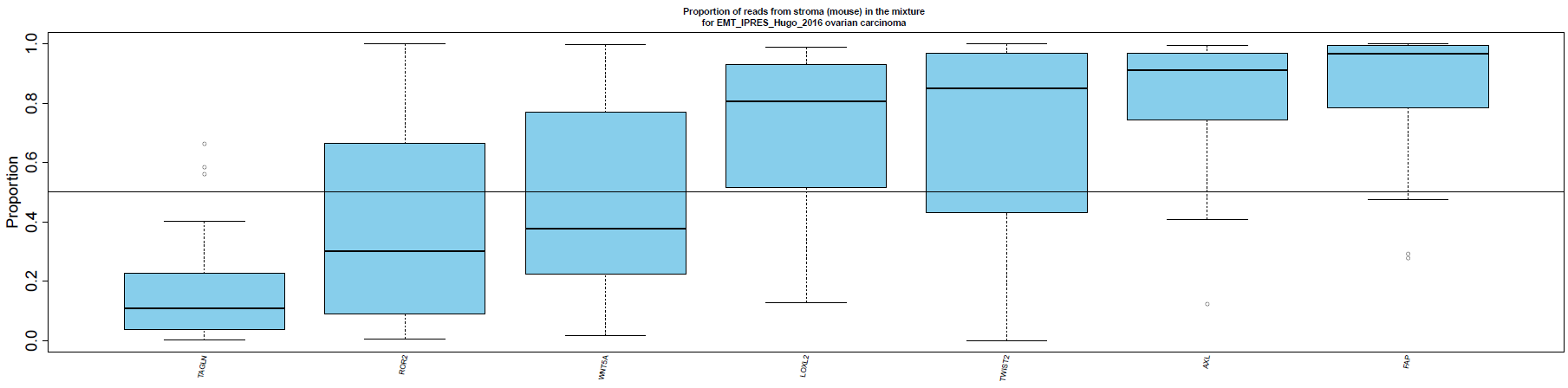

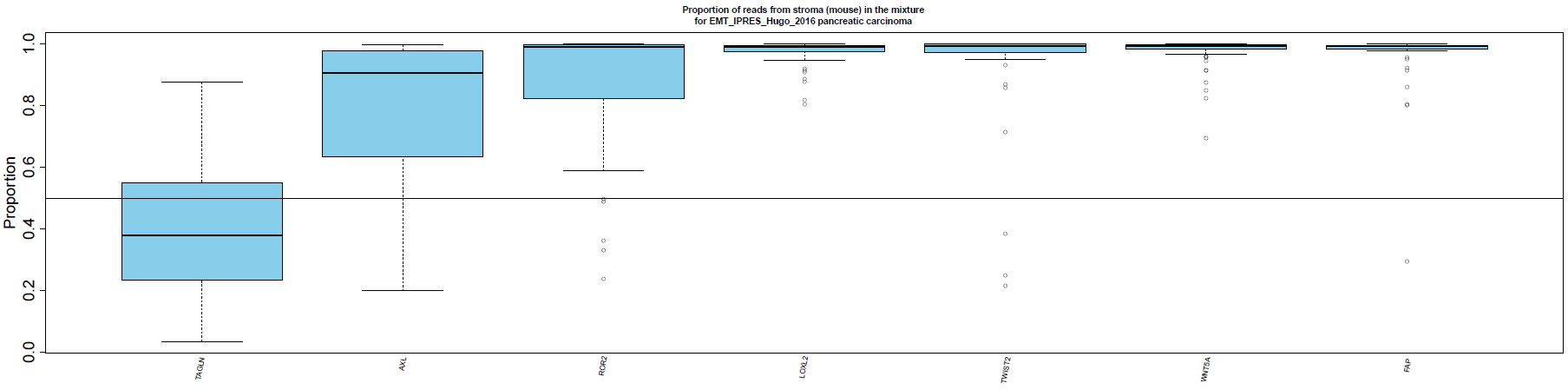

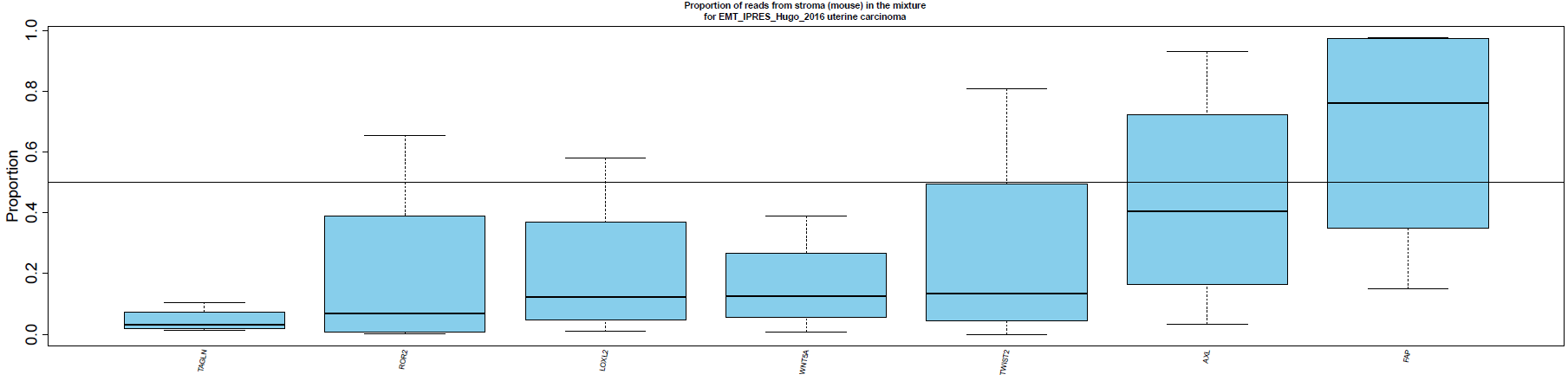


c


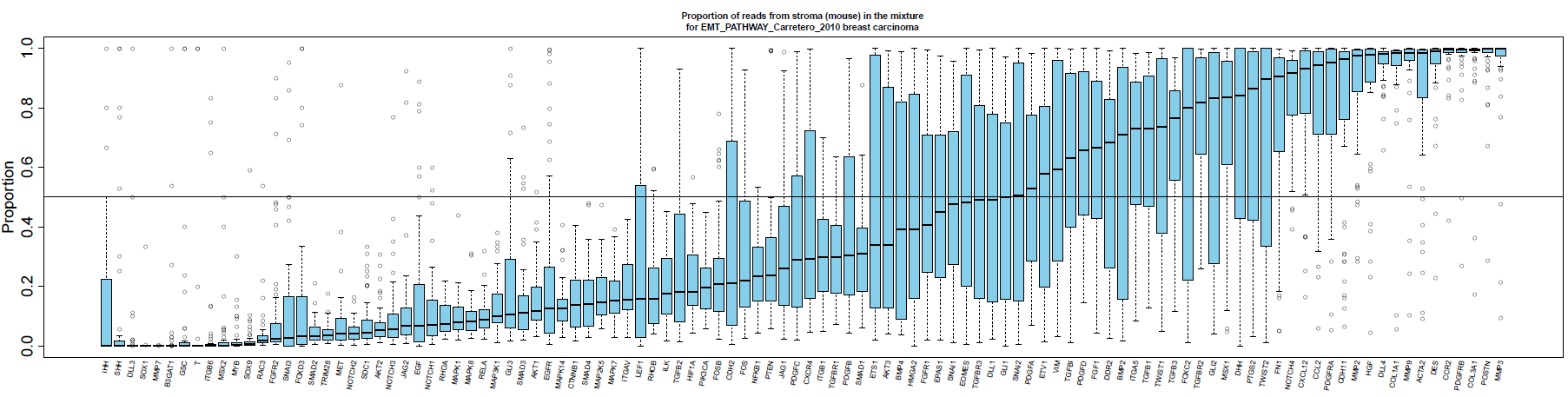

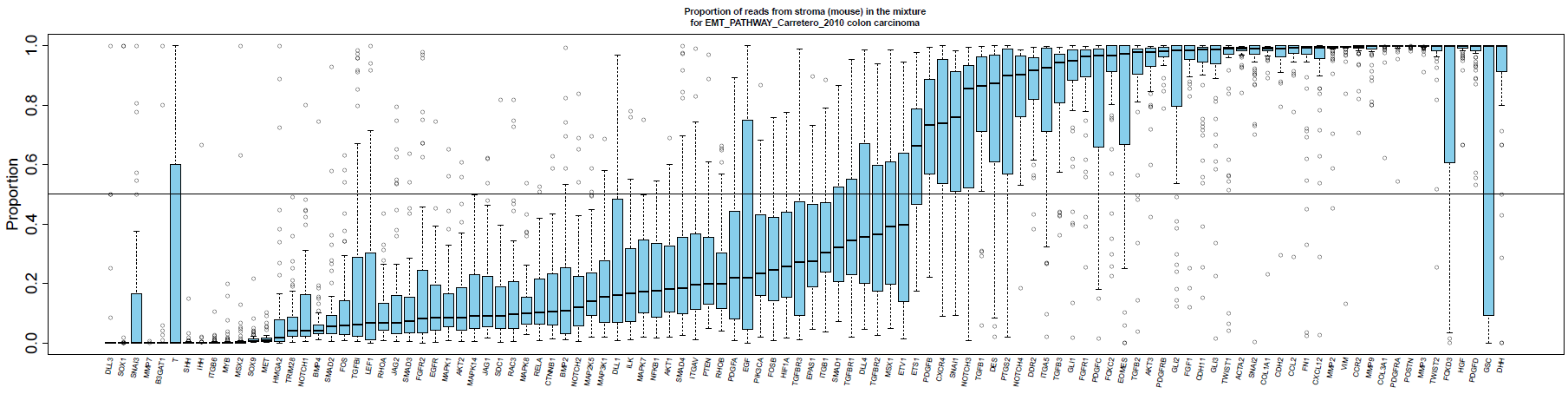

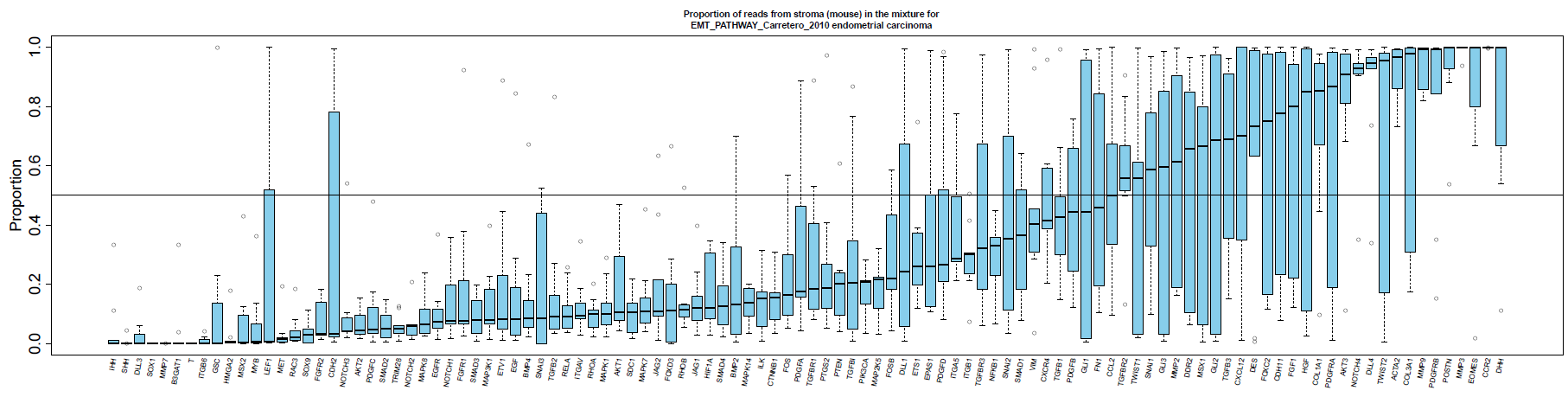

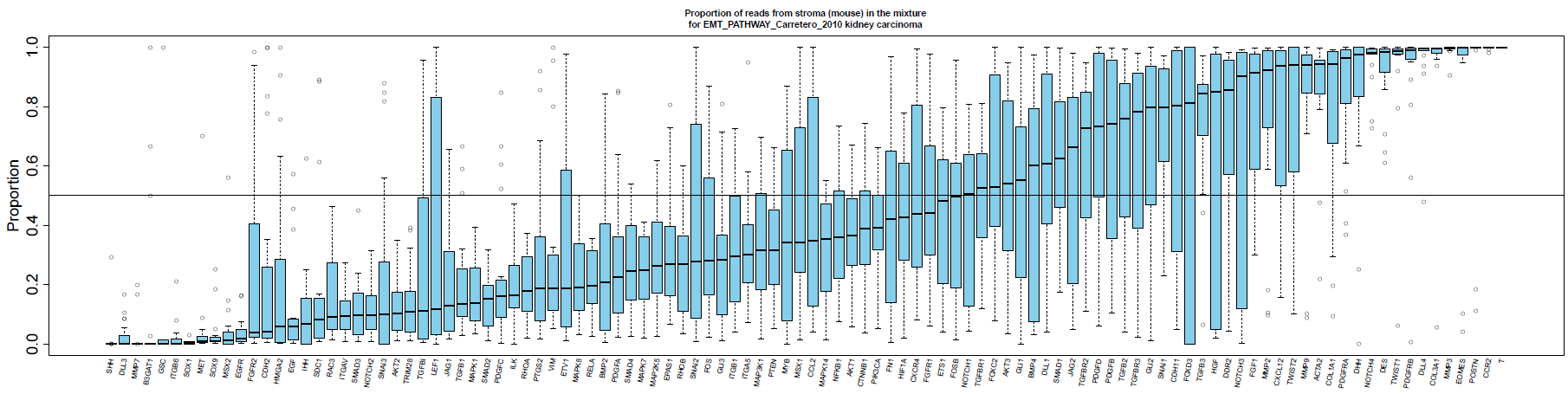

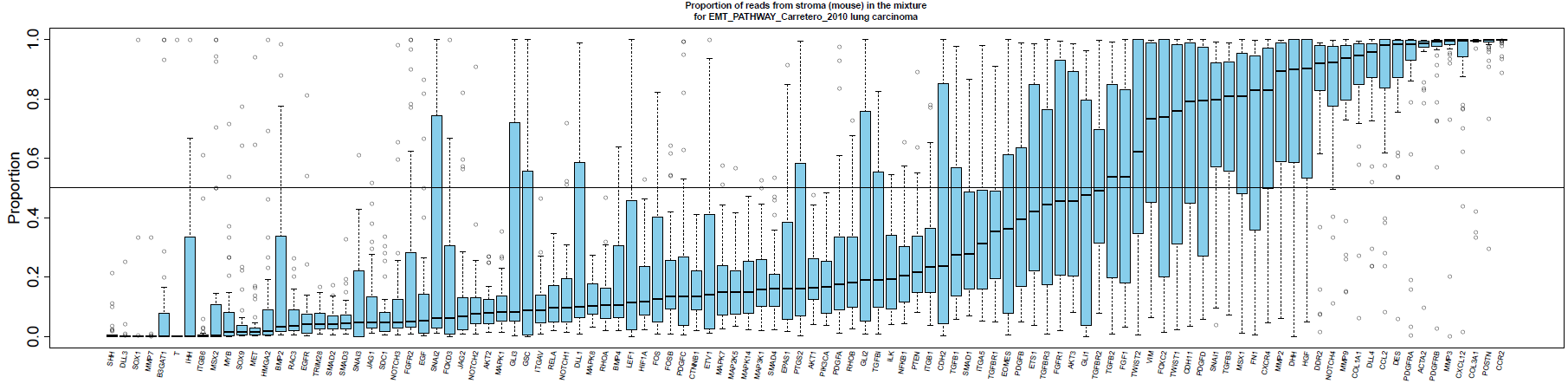

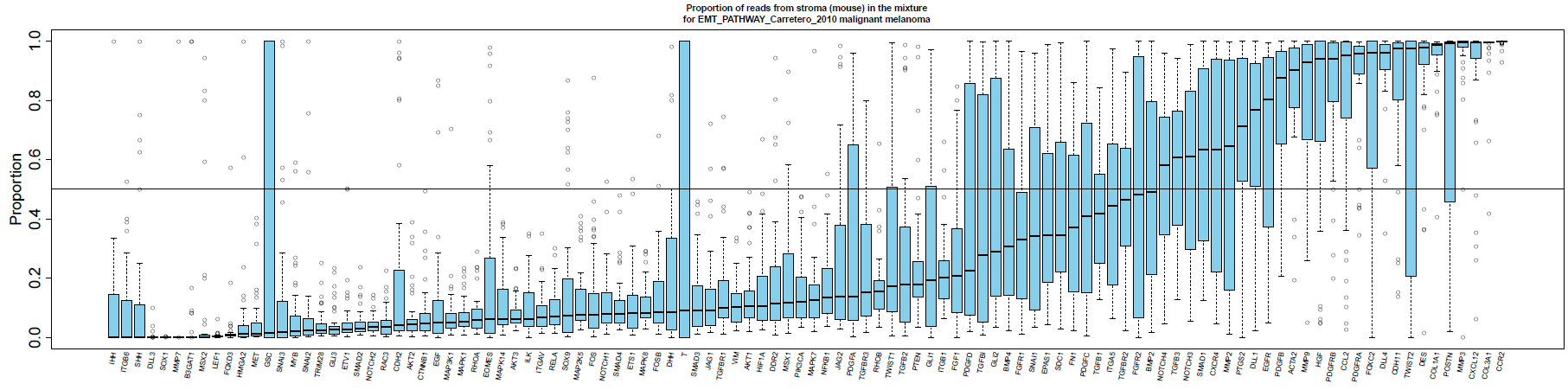

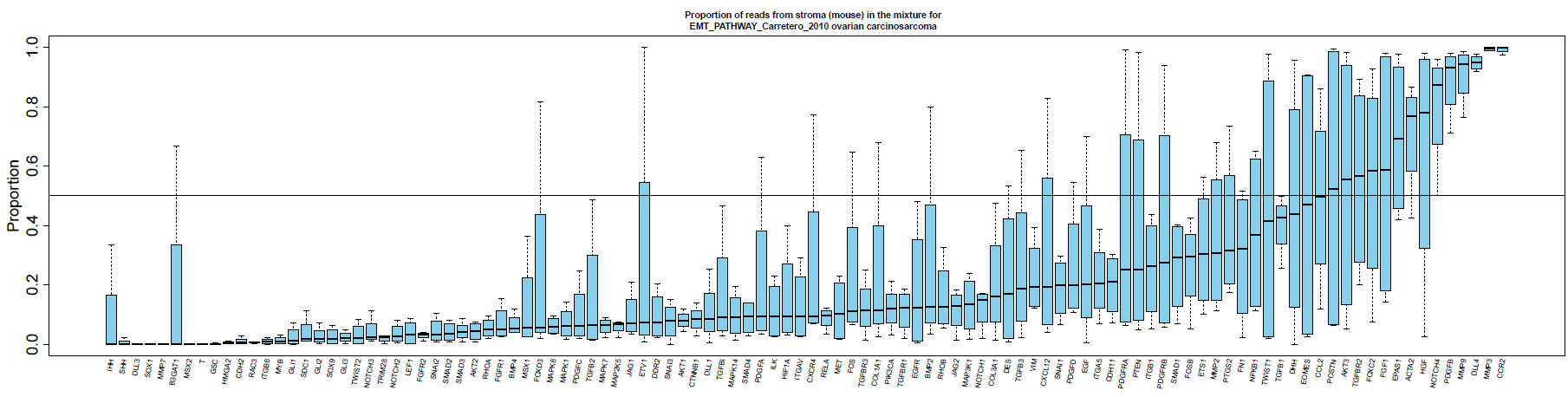

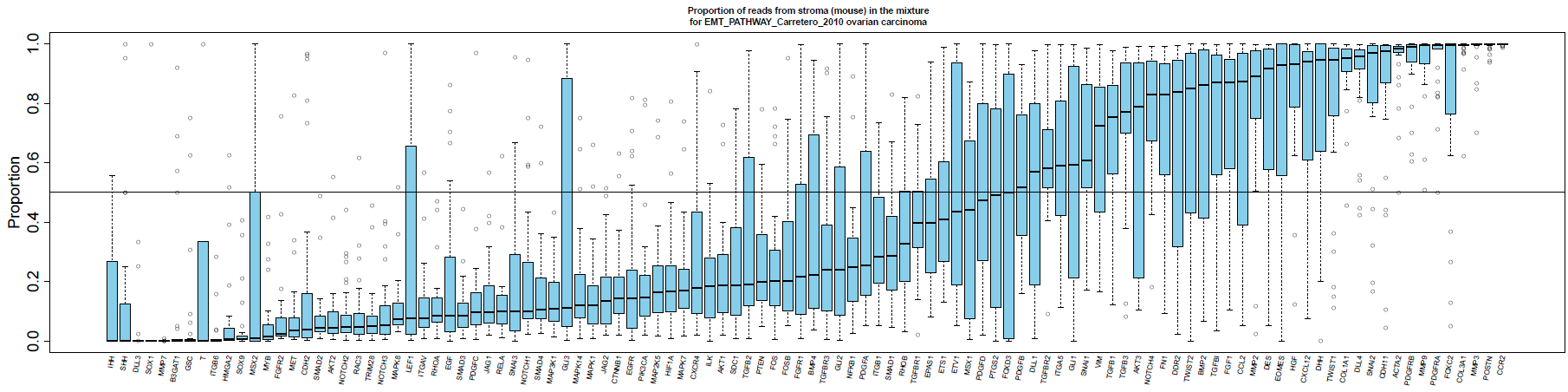

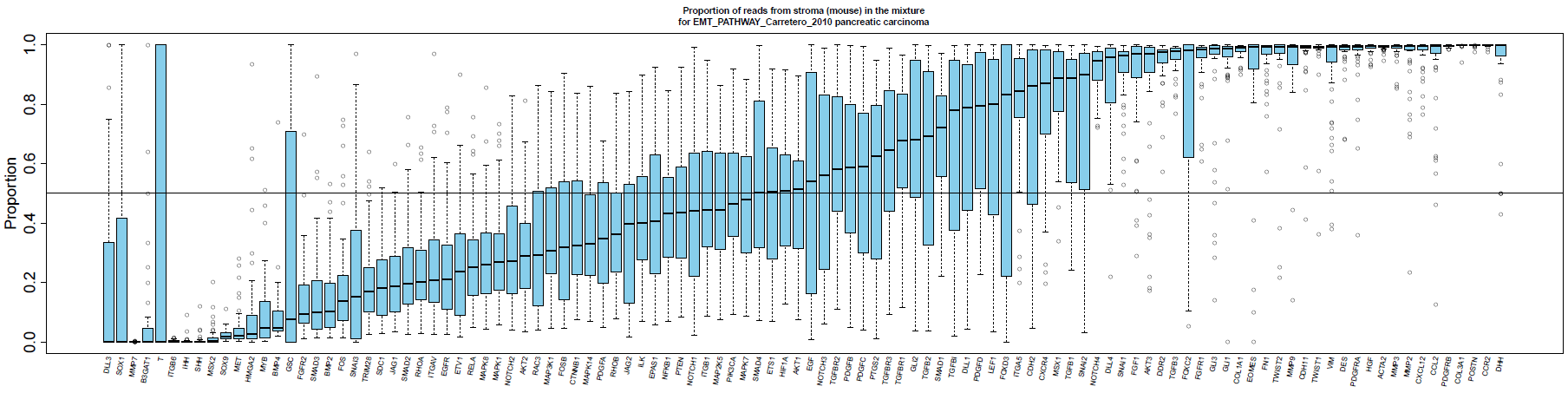

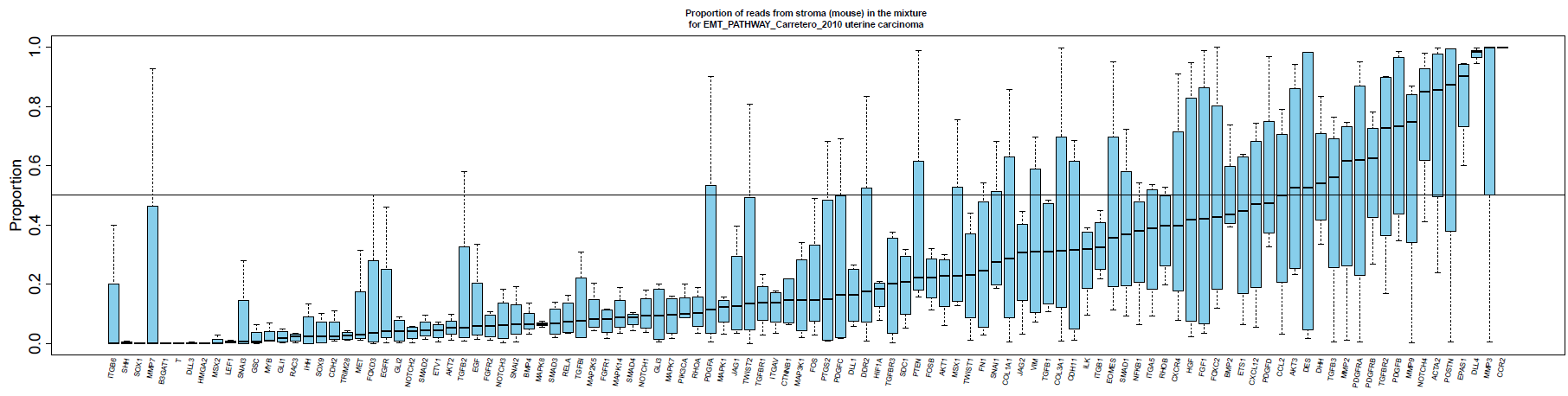


d


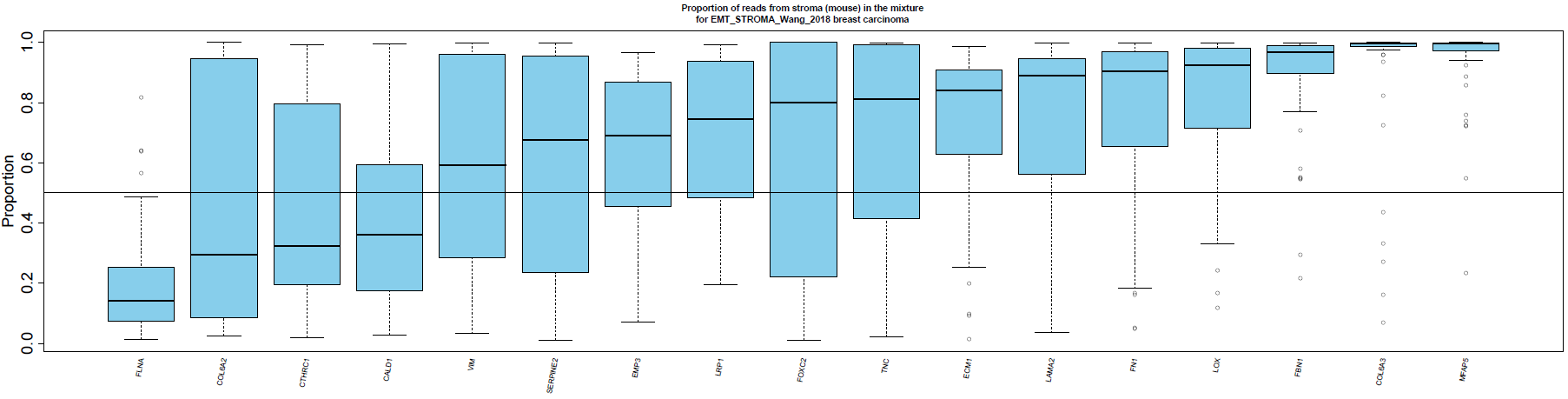

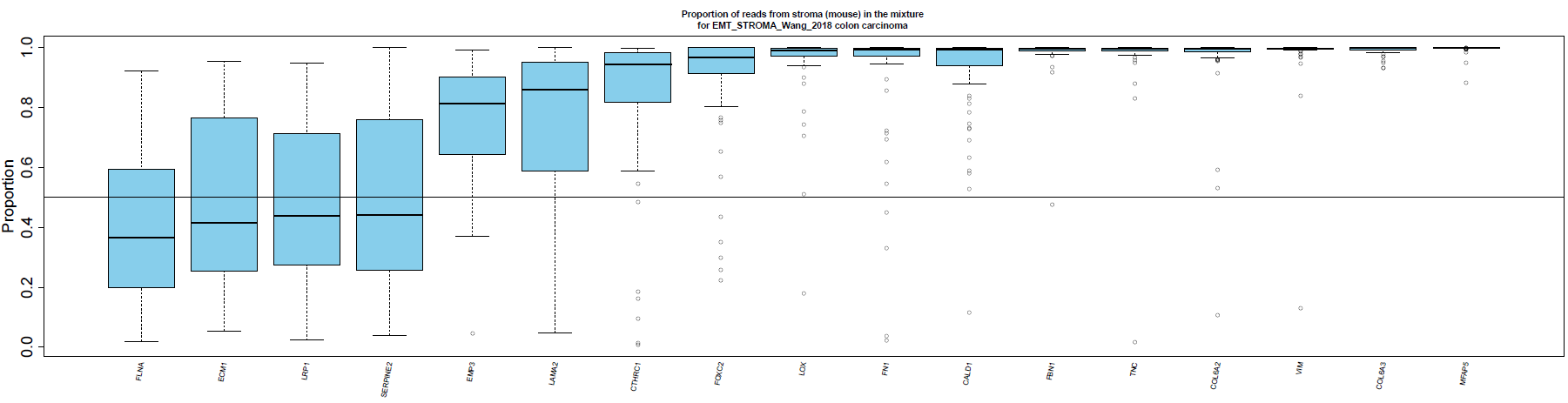

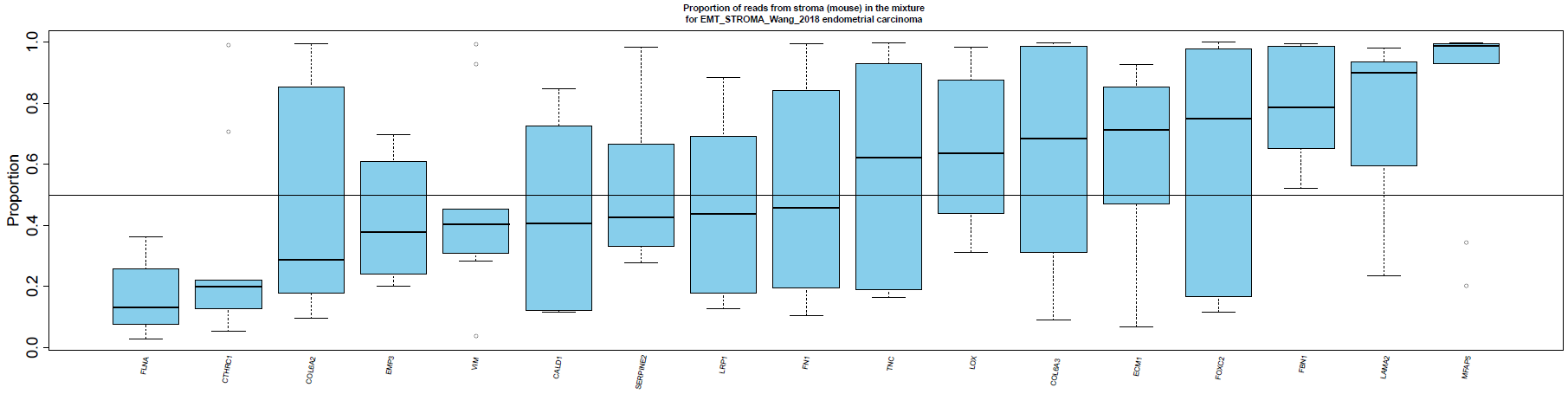

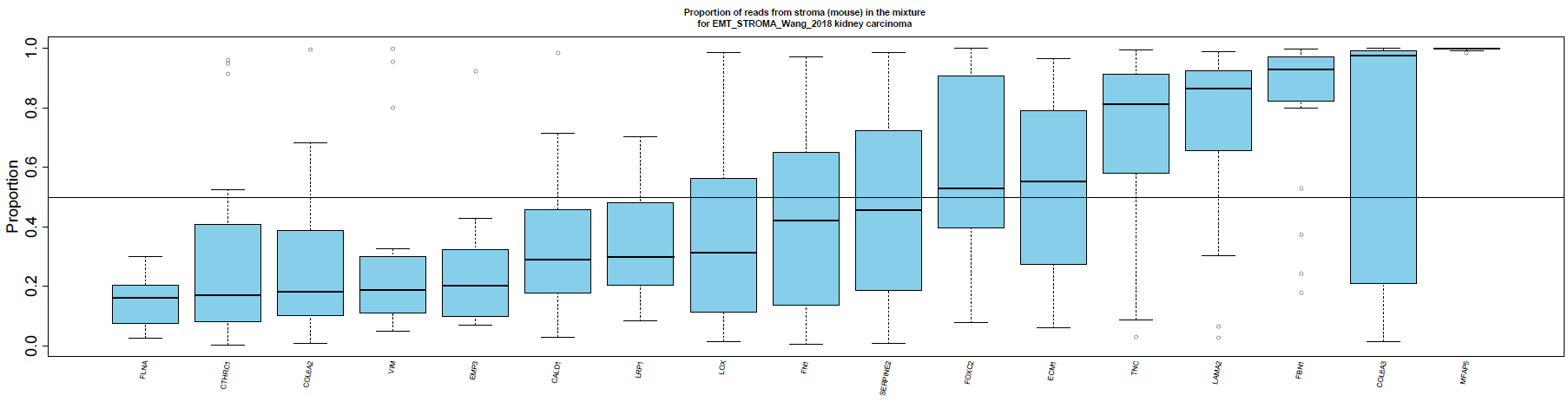

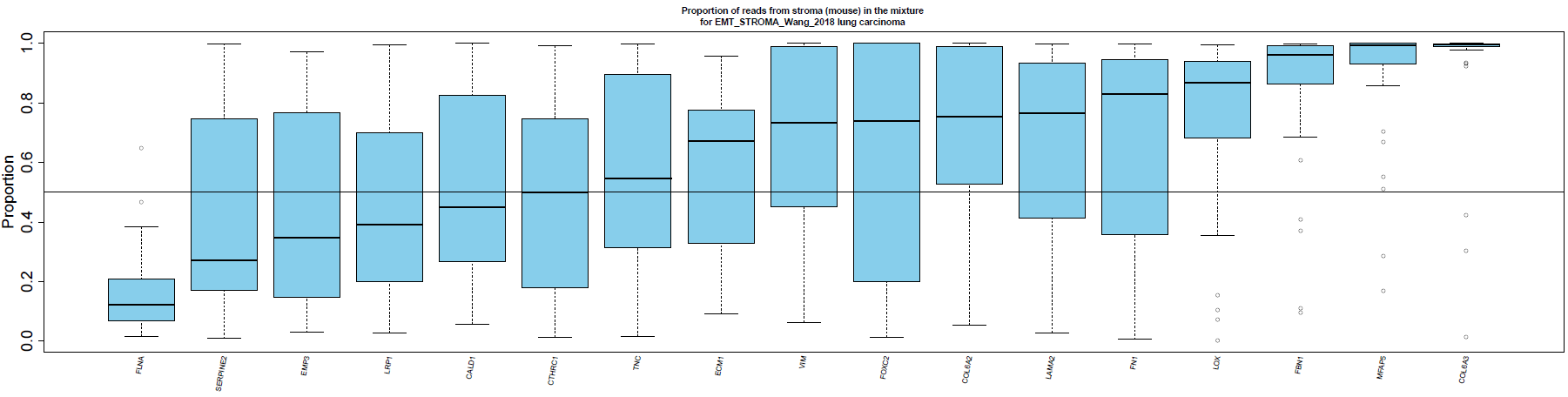

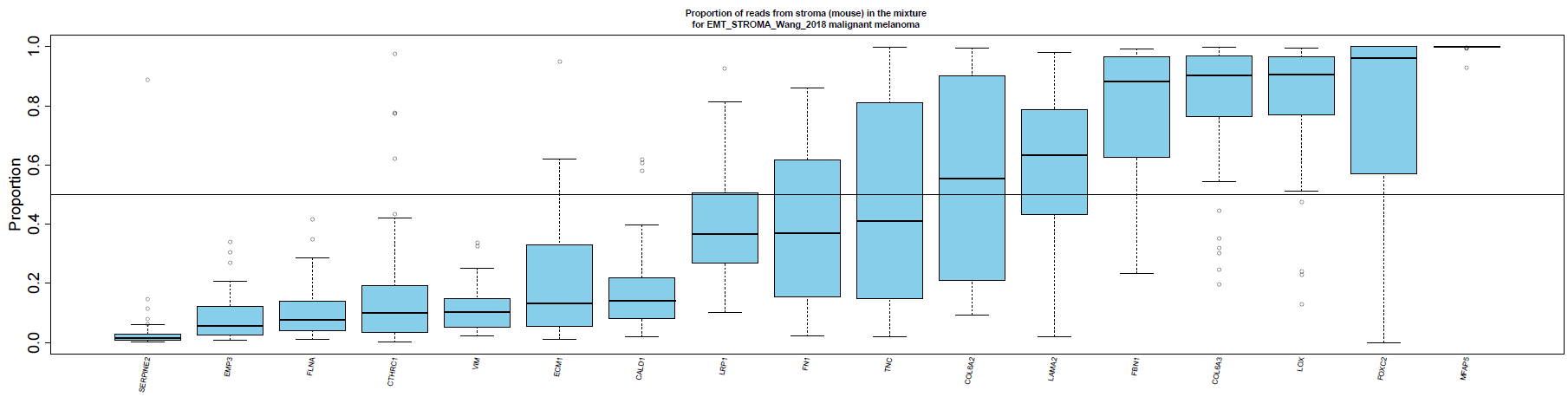

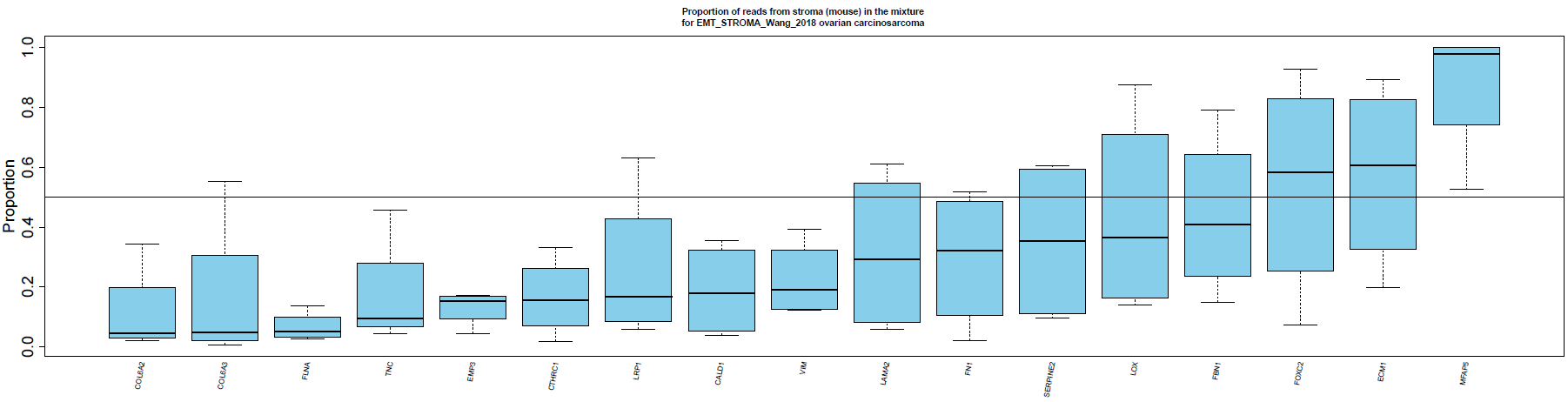

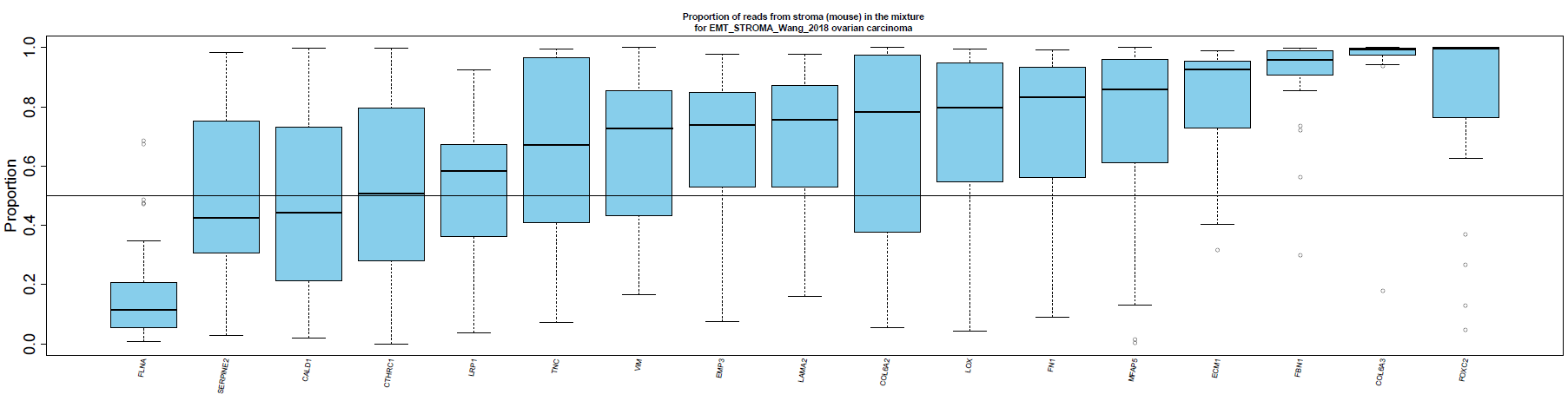

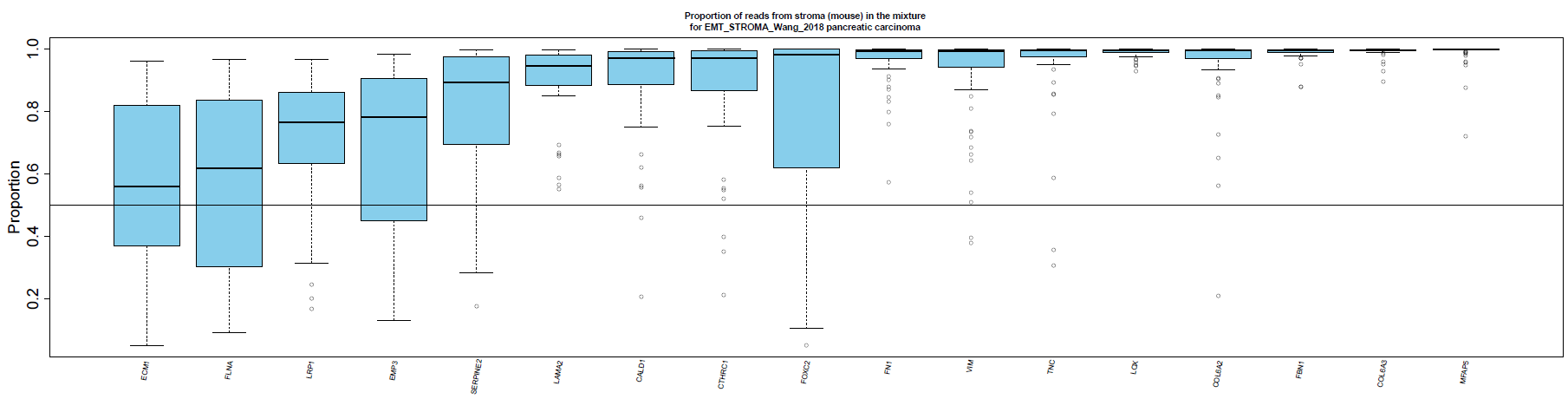

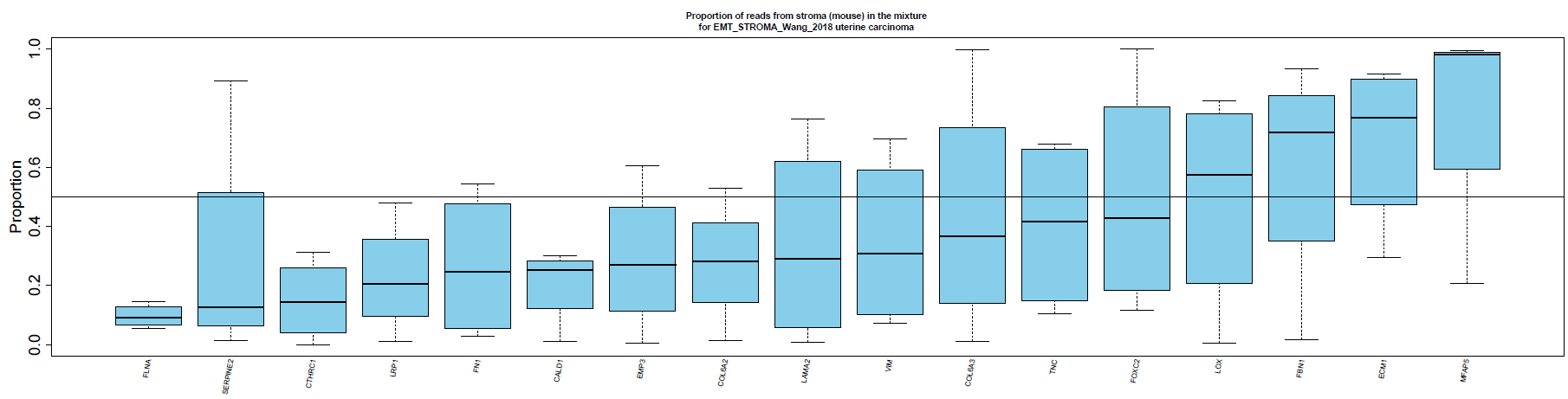


e


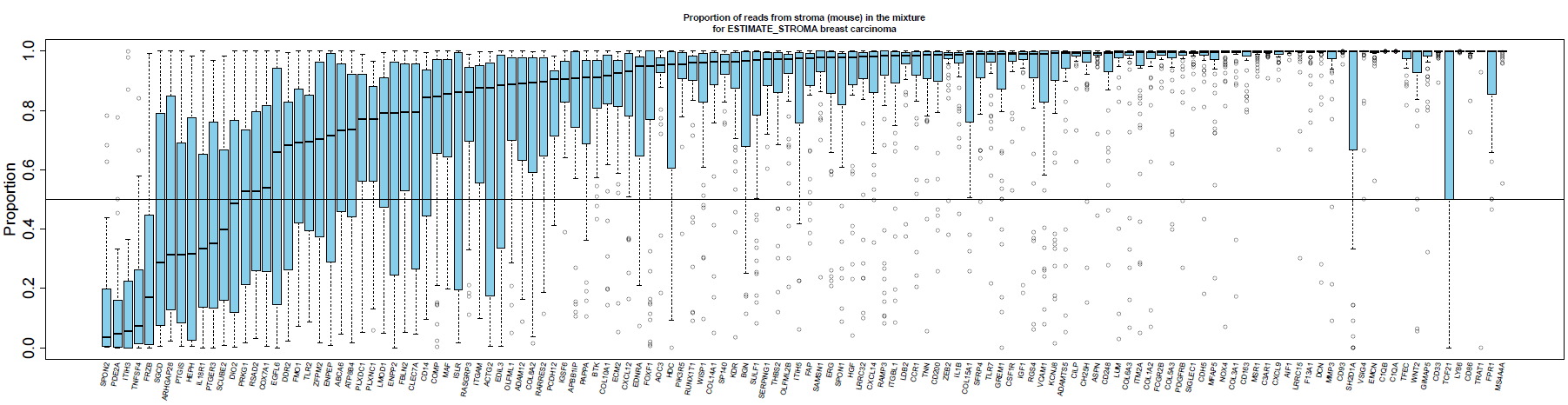

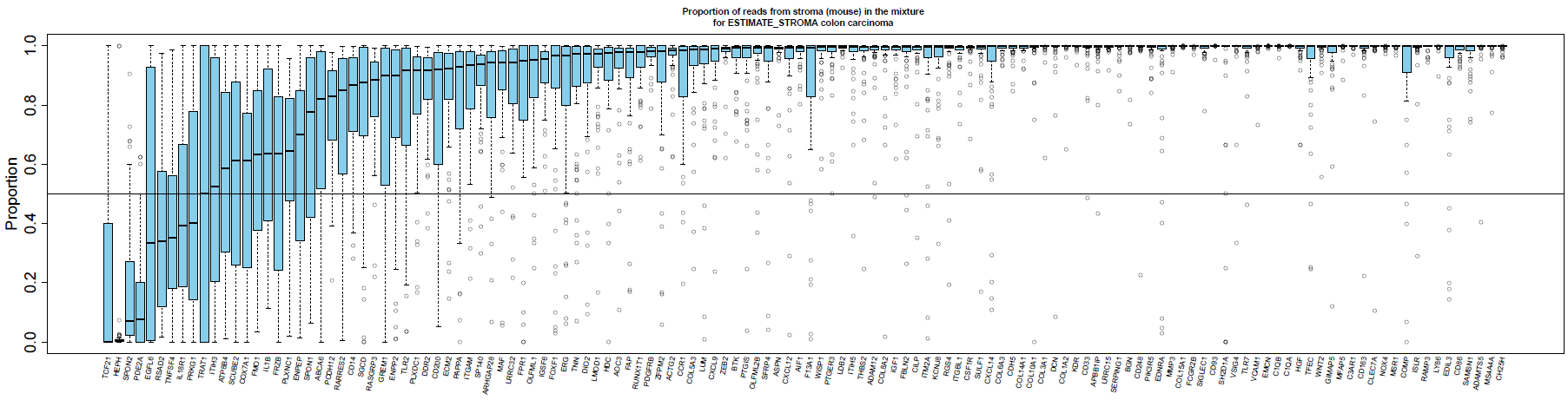

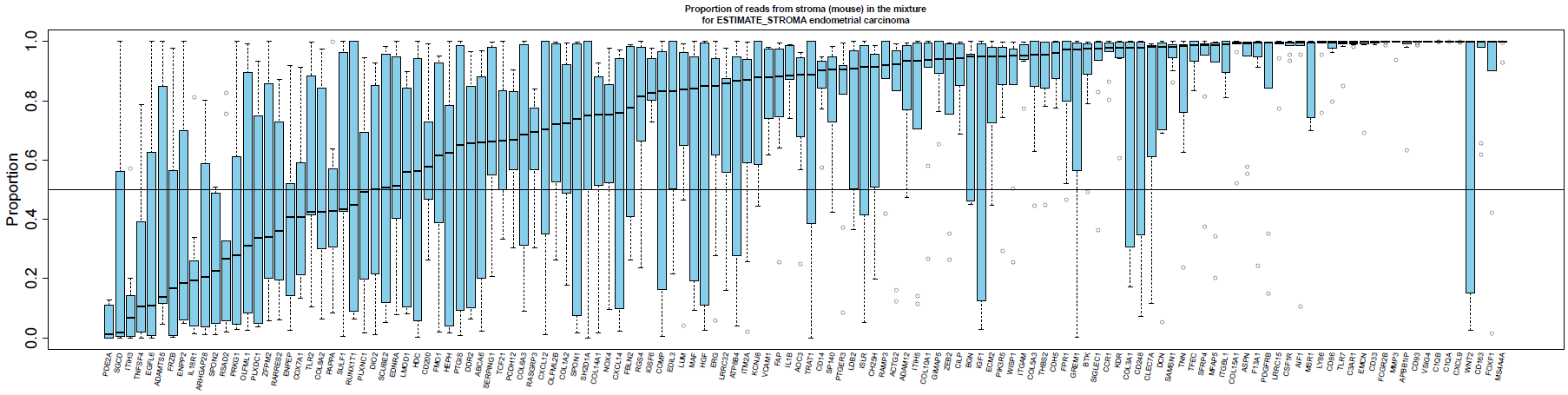

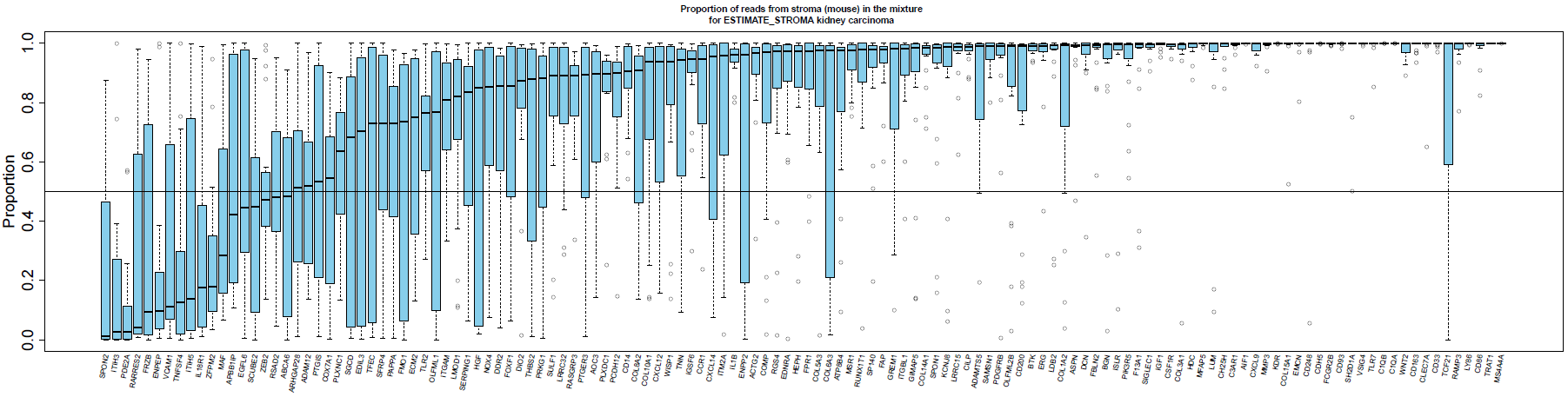

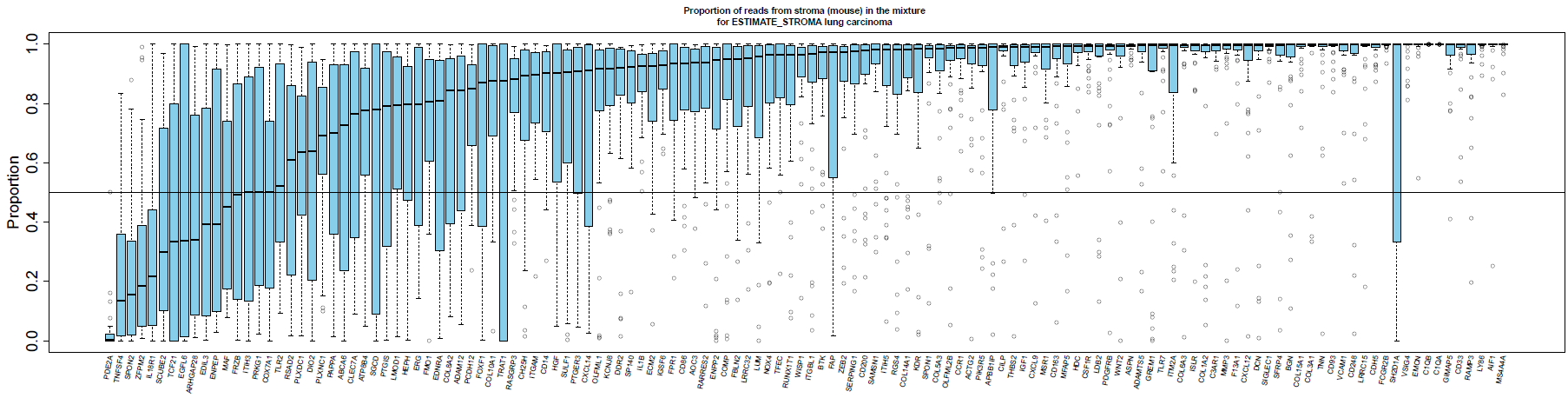

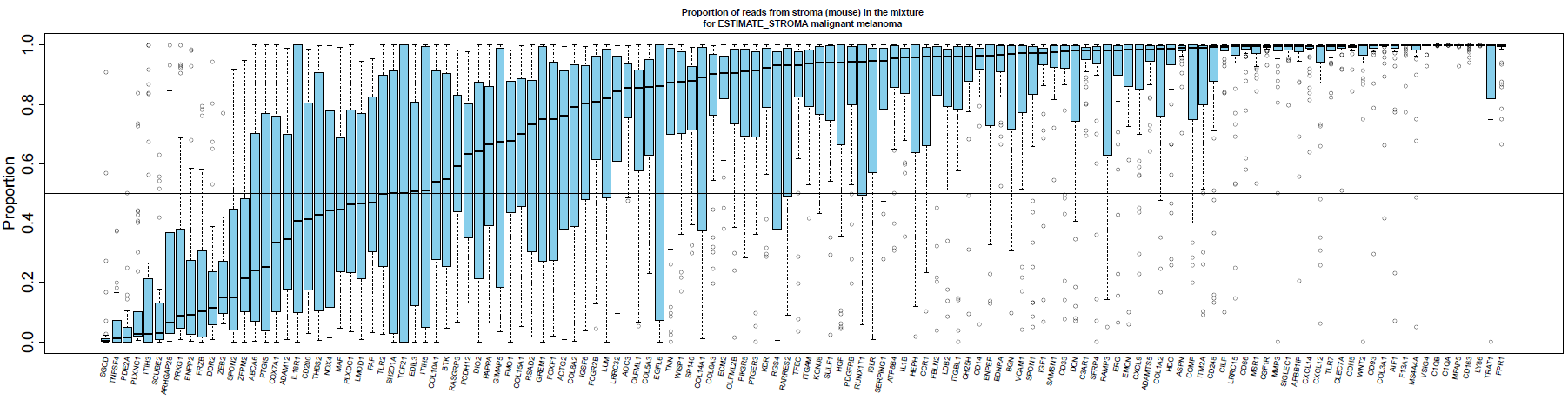

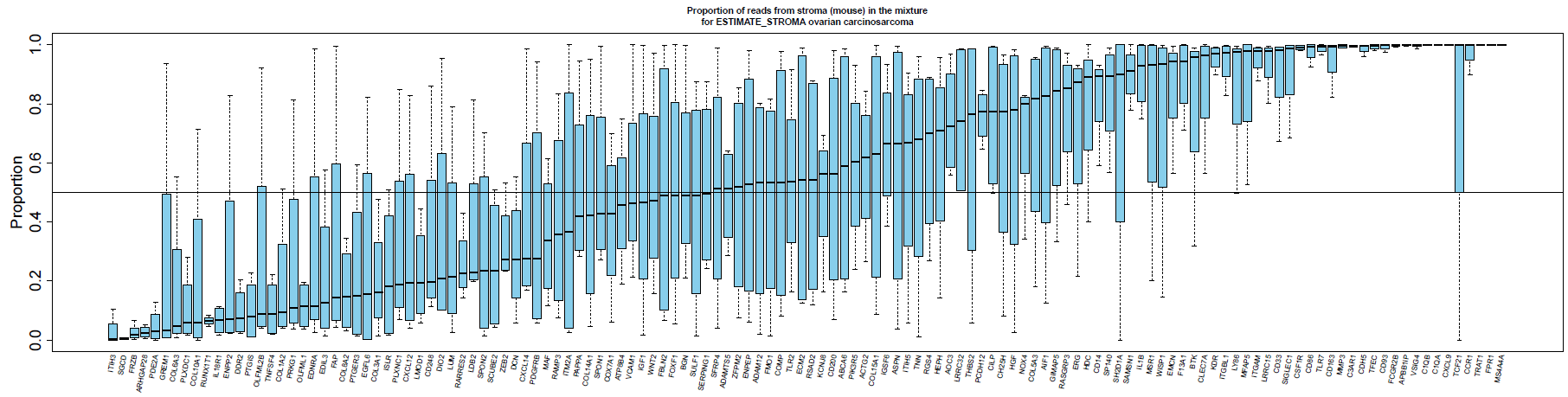

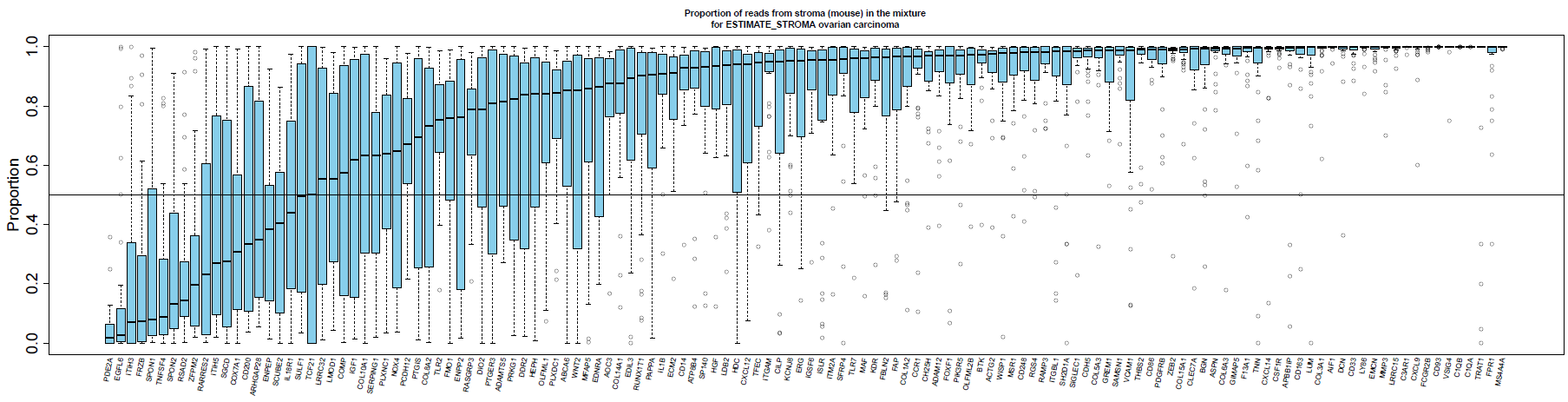

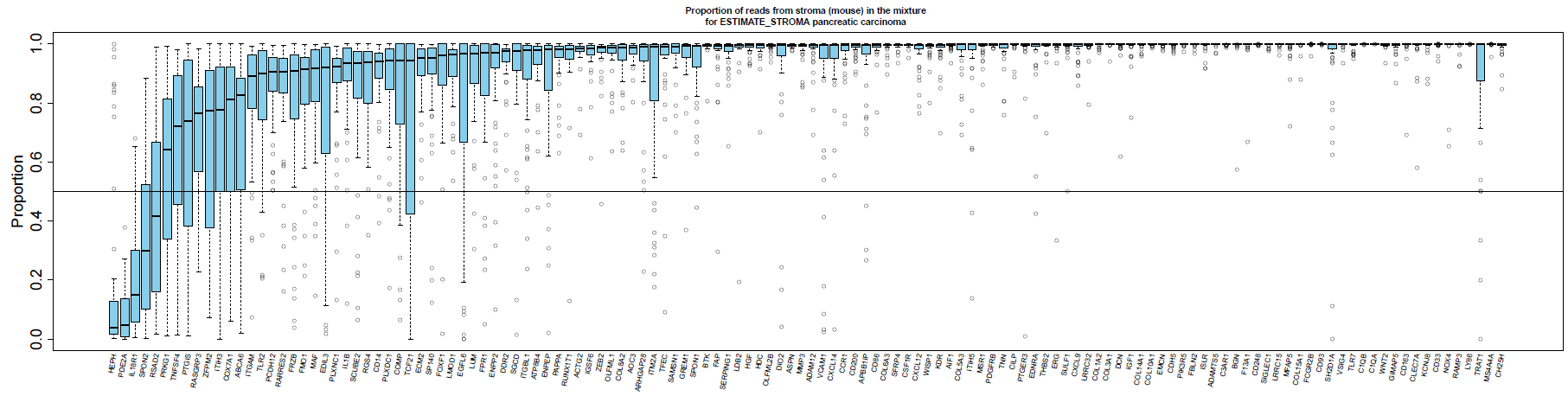

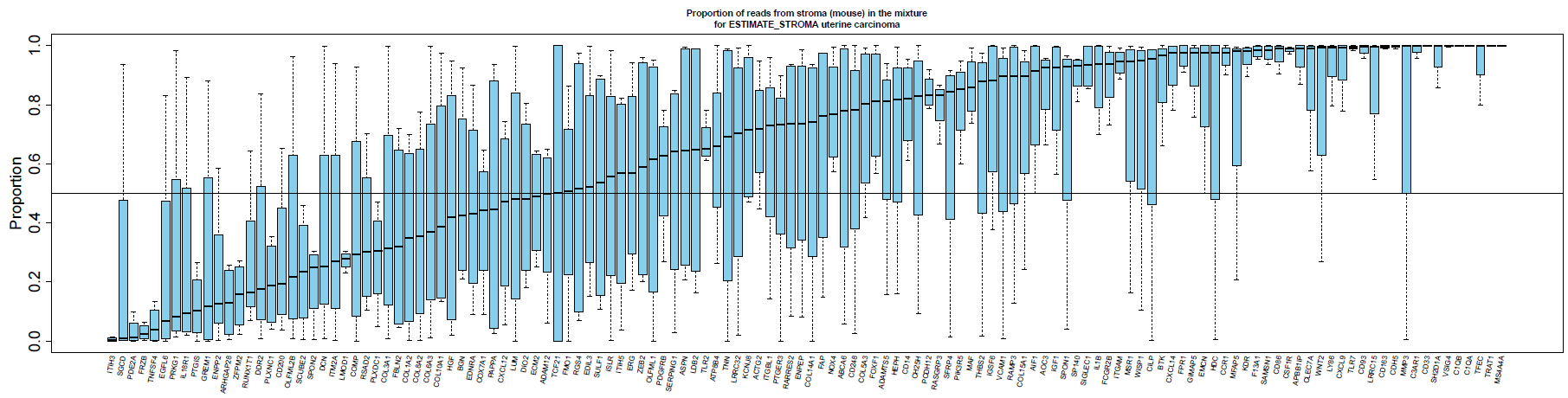


f

**
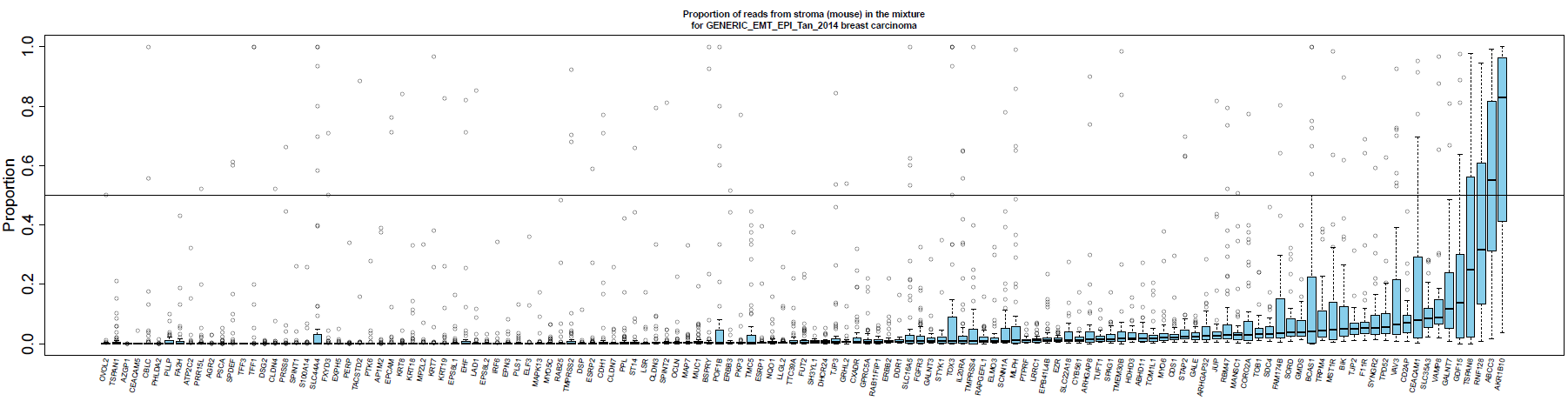

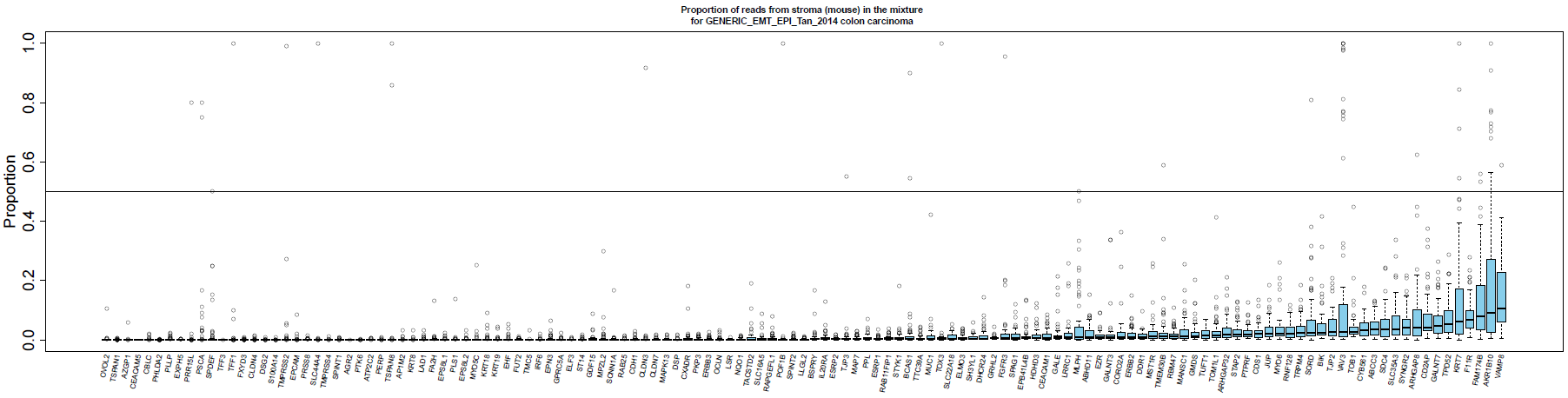

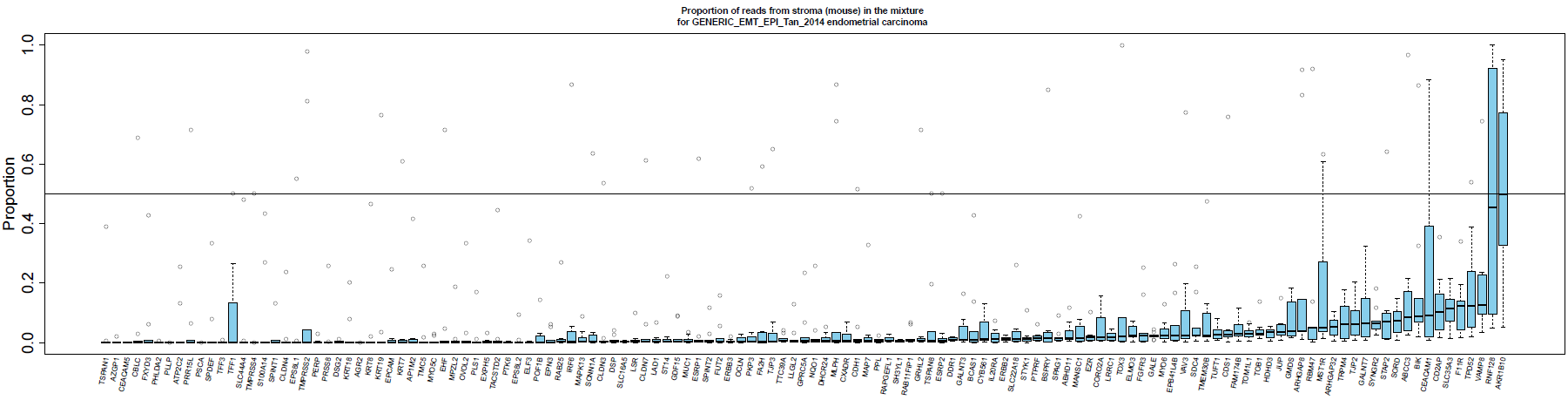

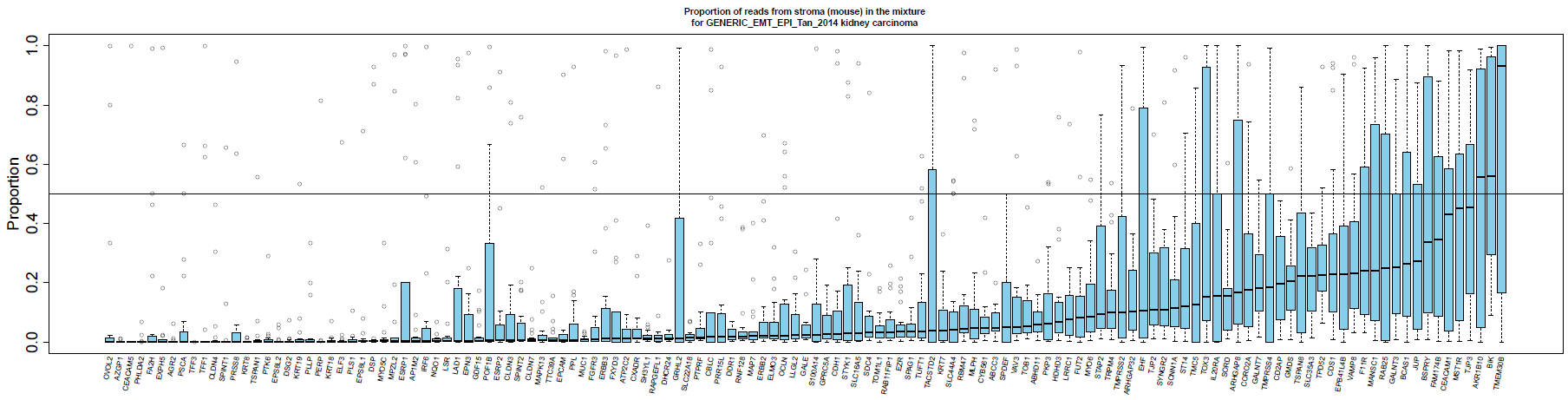

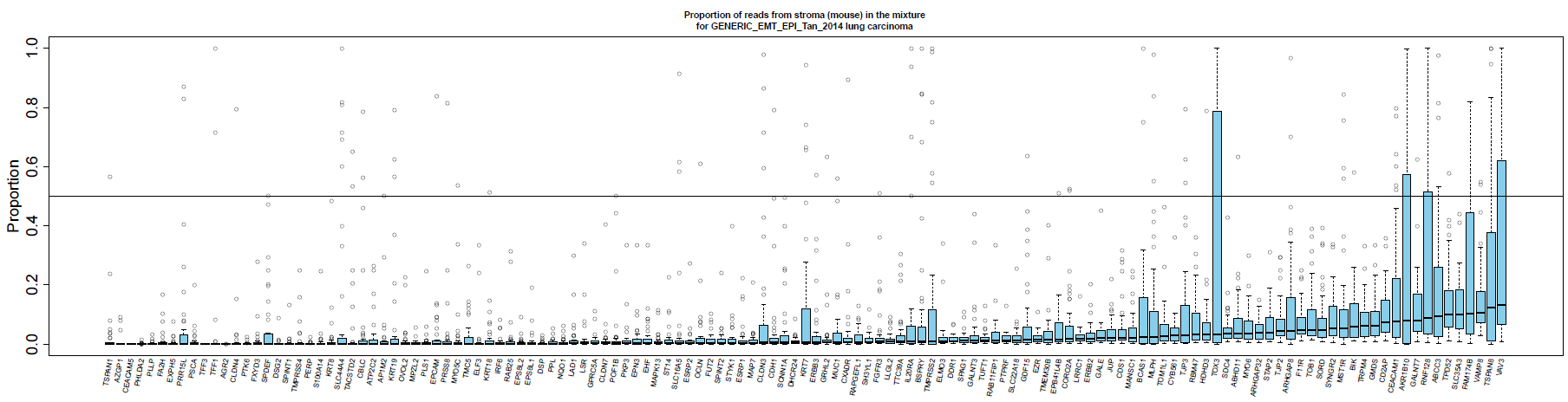

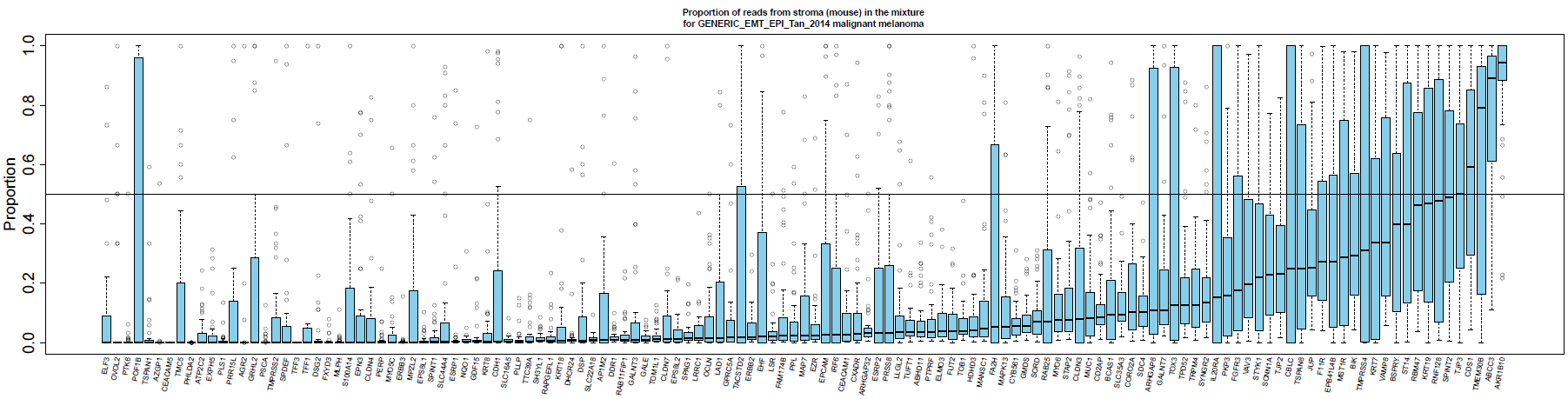

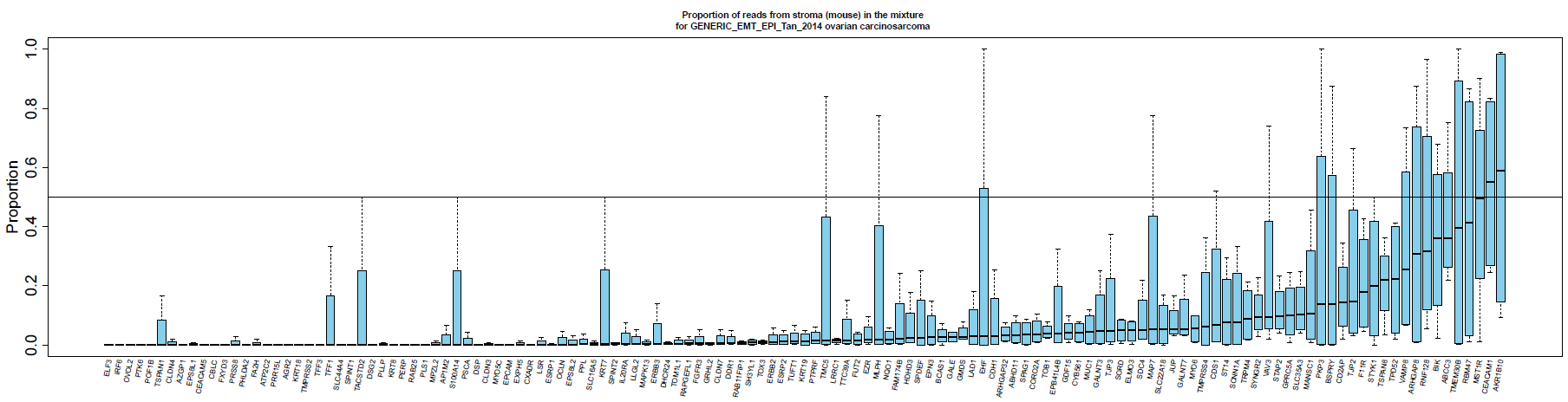

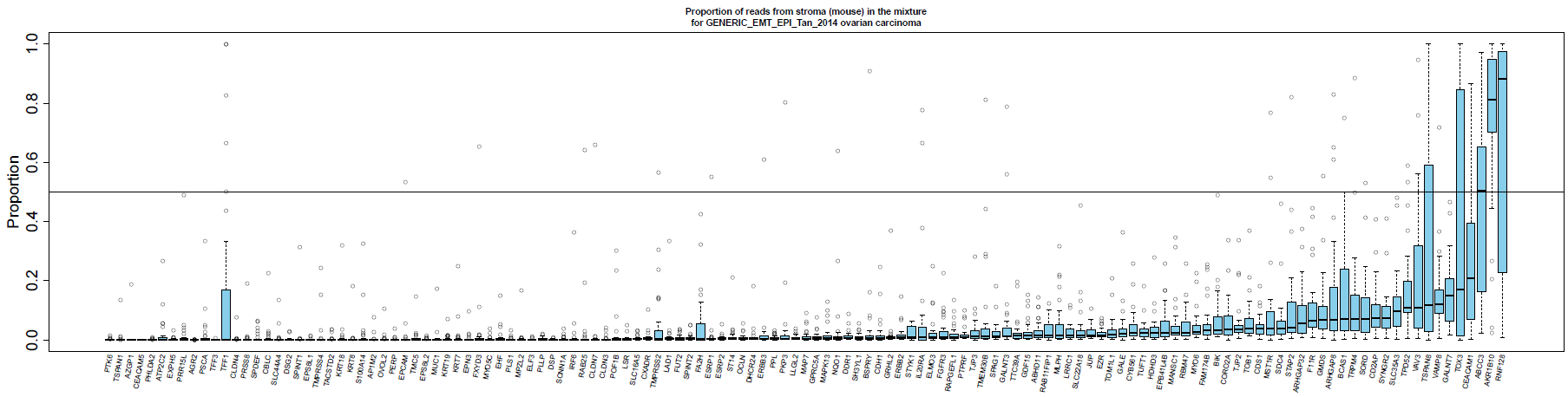

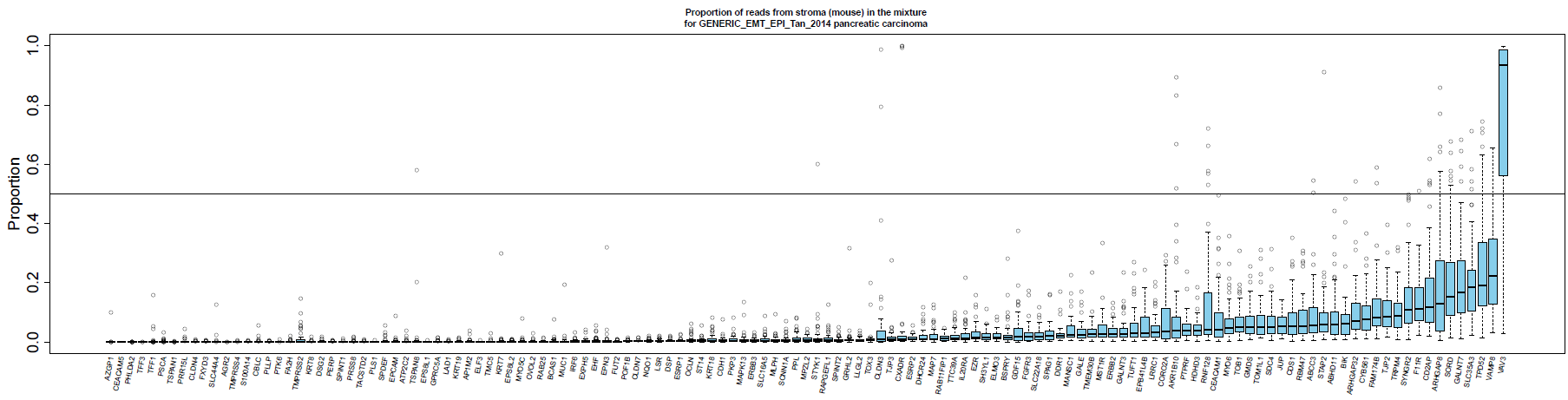

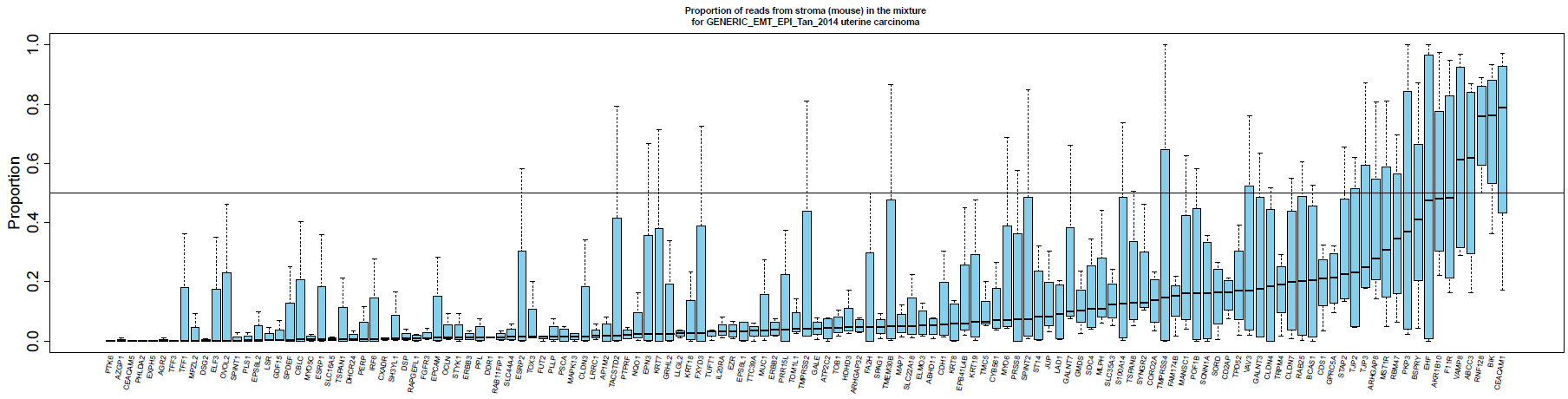
**

g


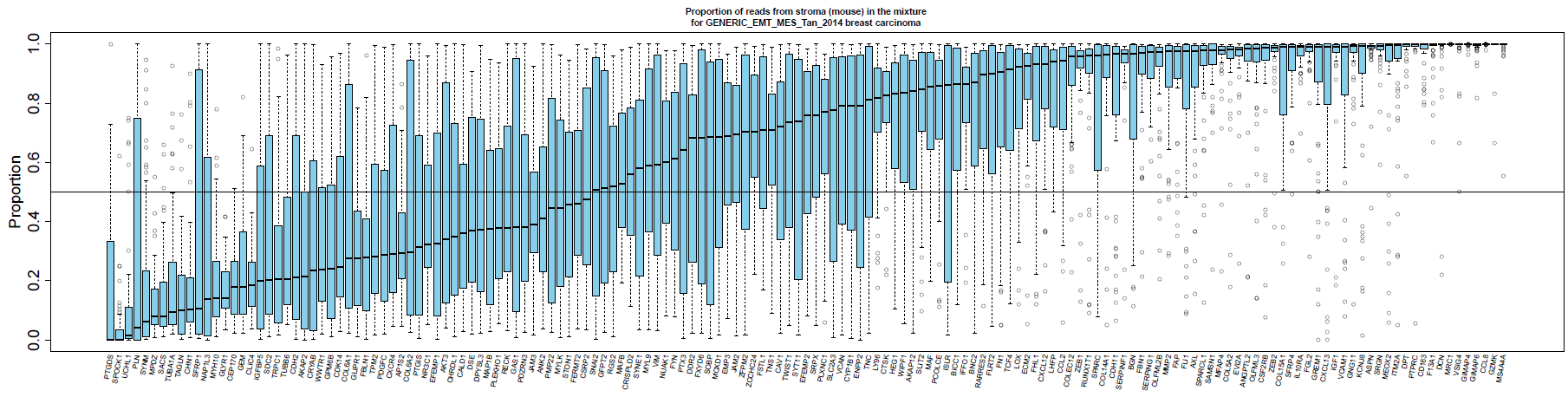

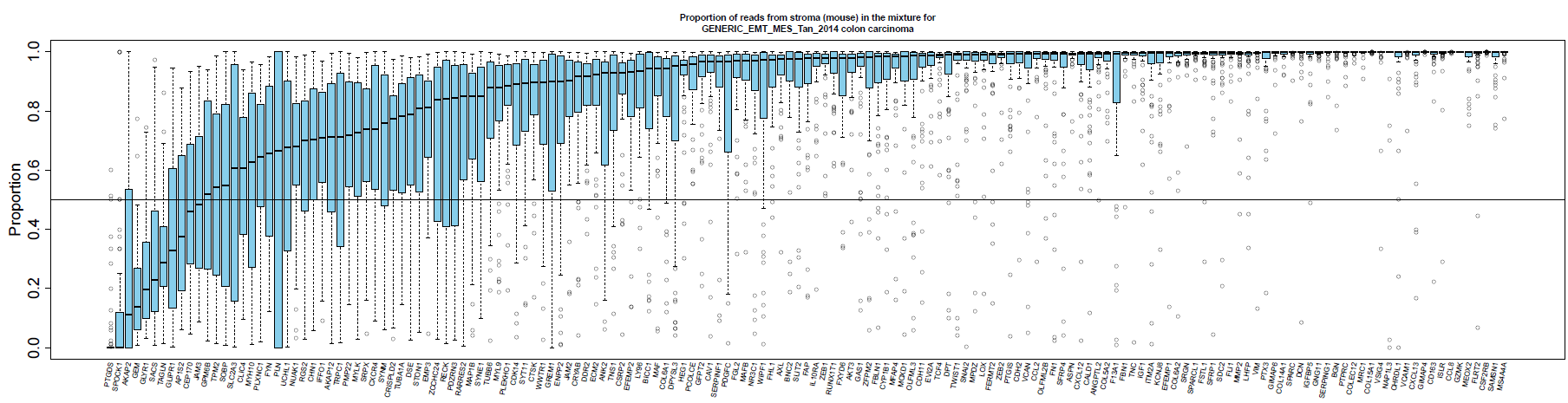

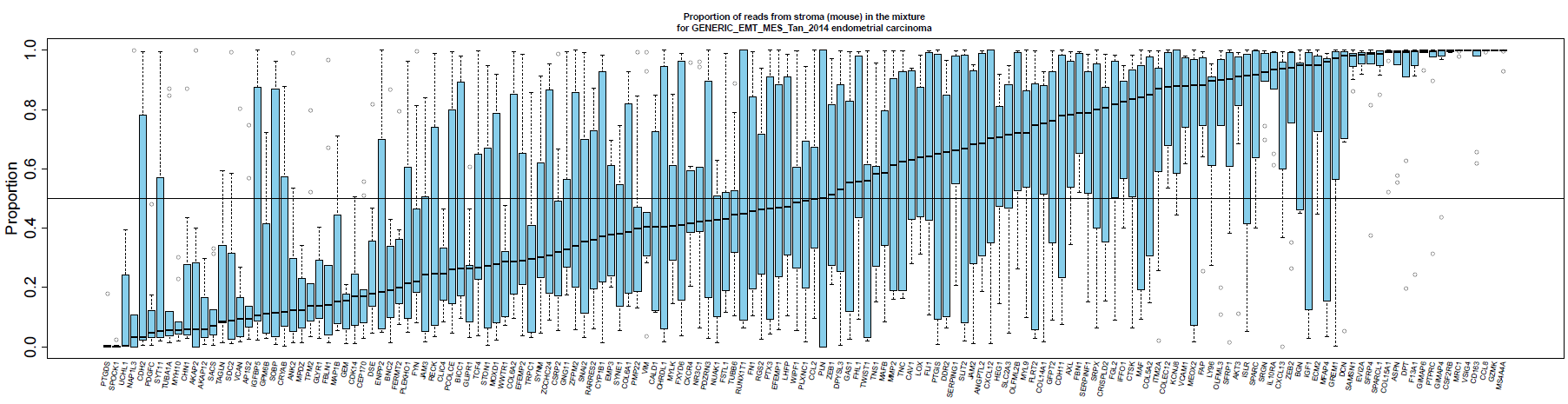

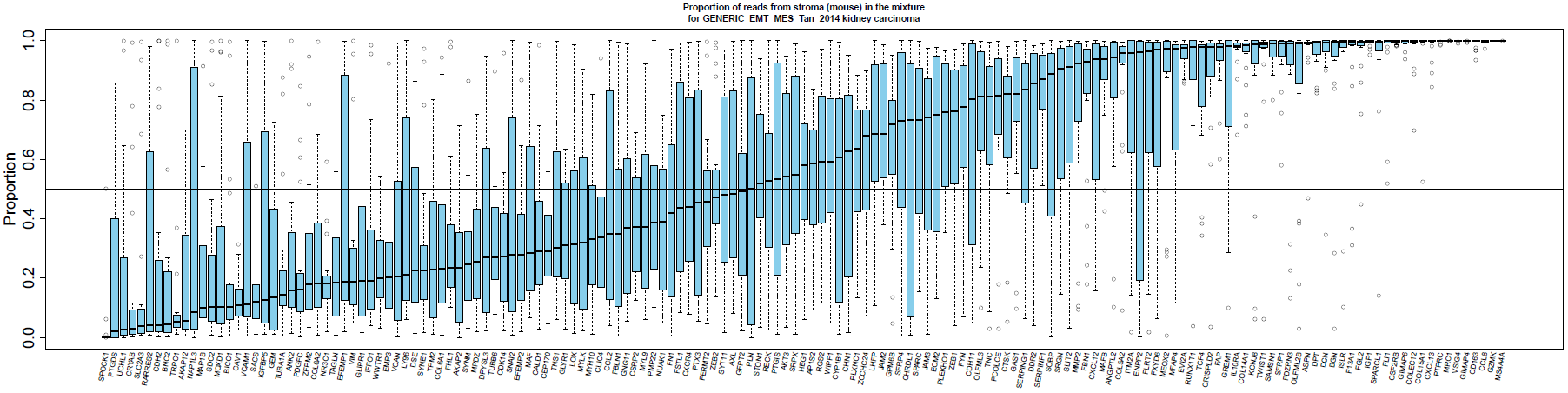

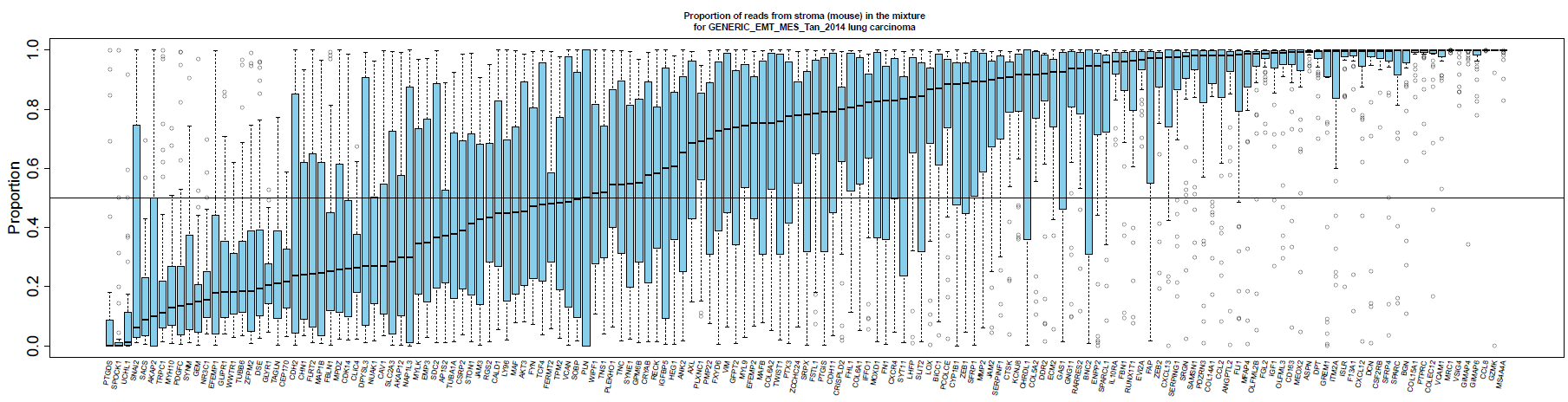

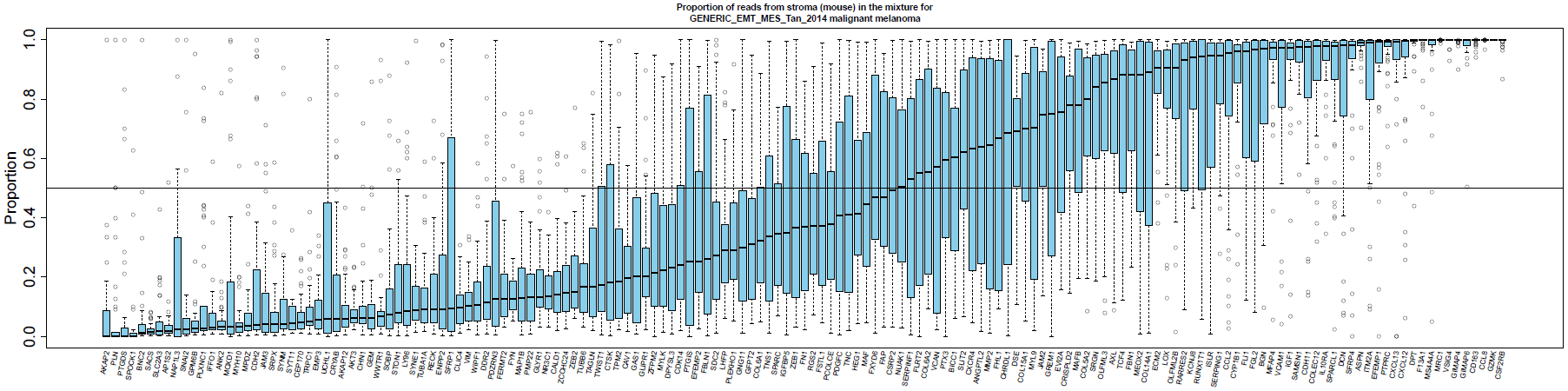

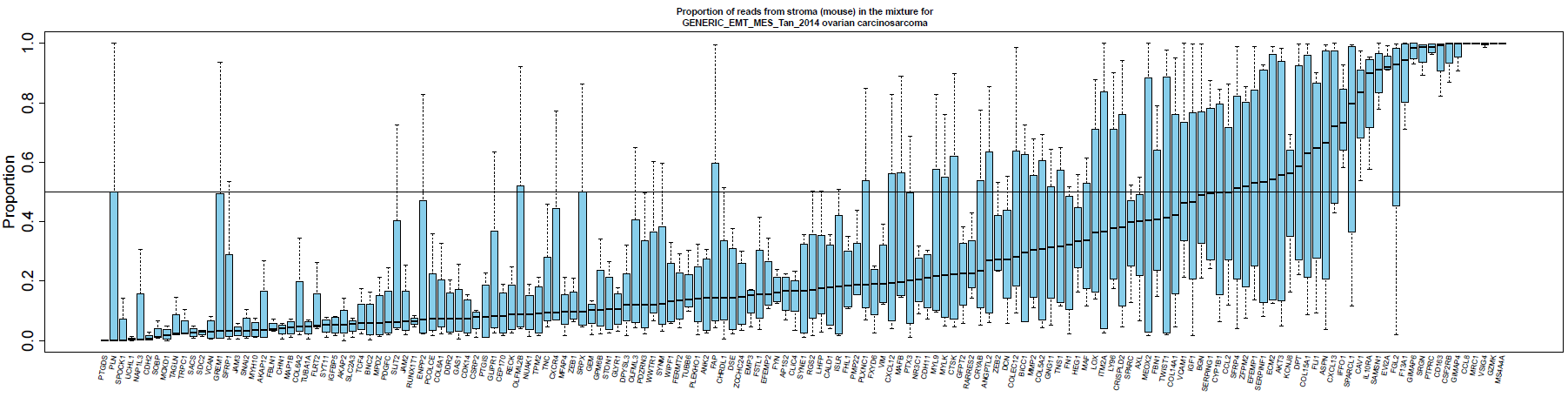

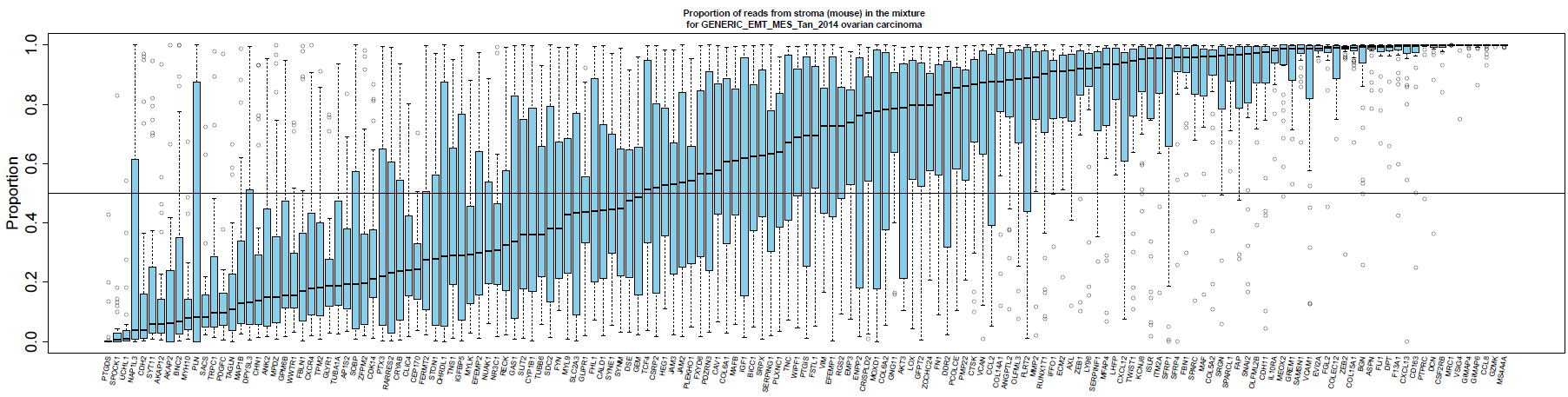

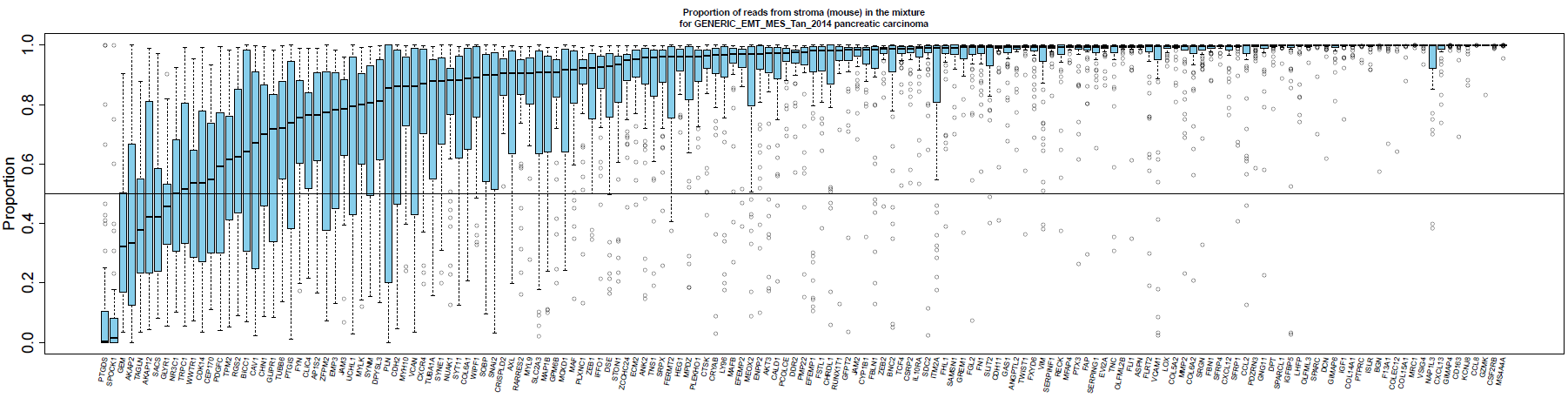

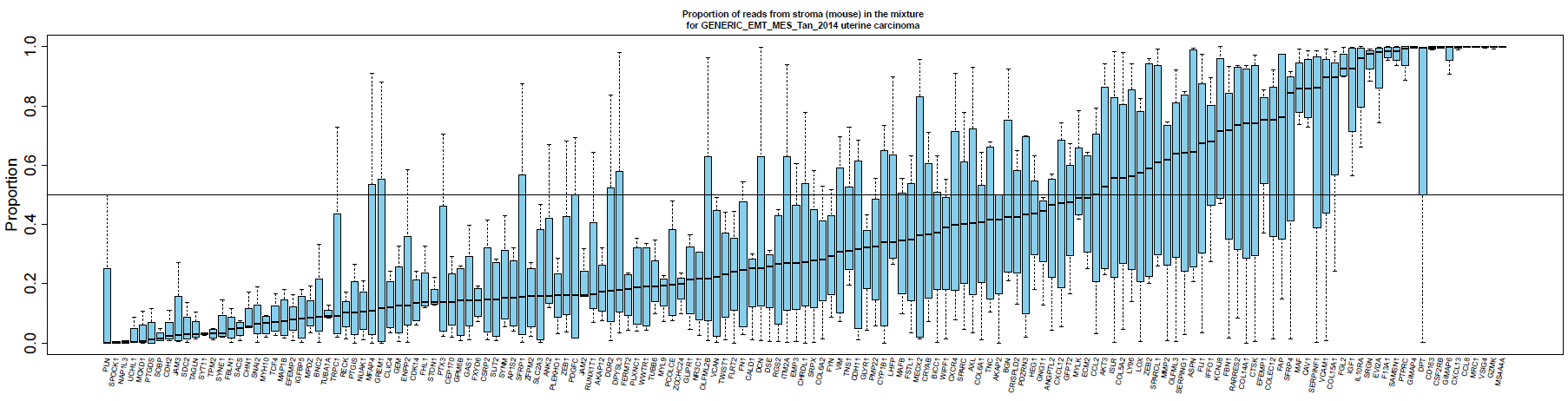


h


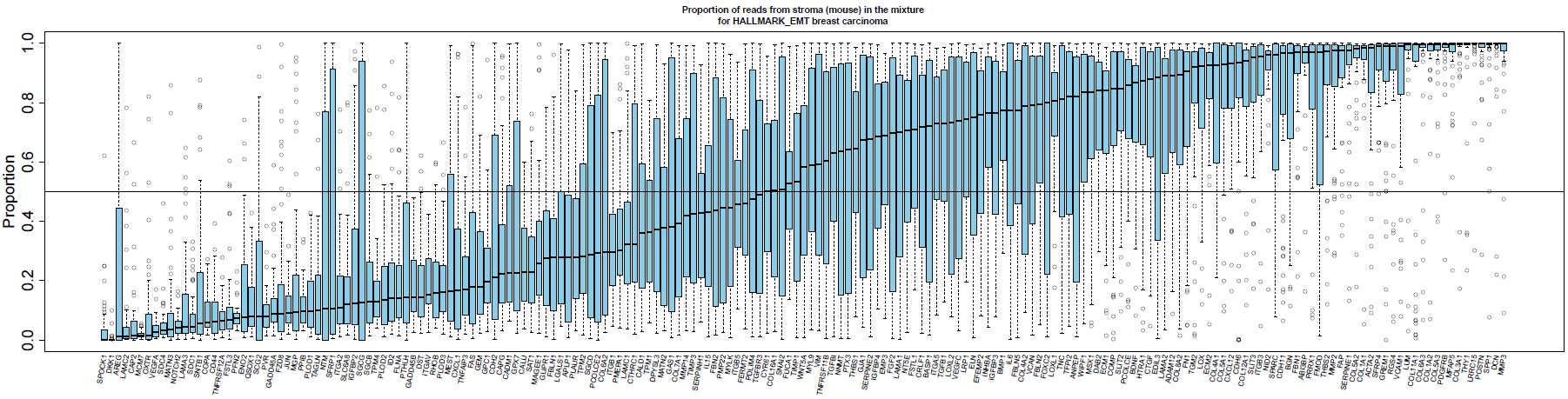

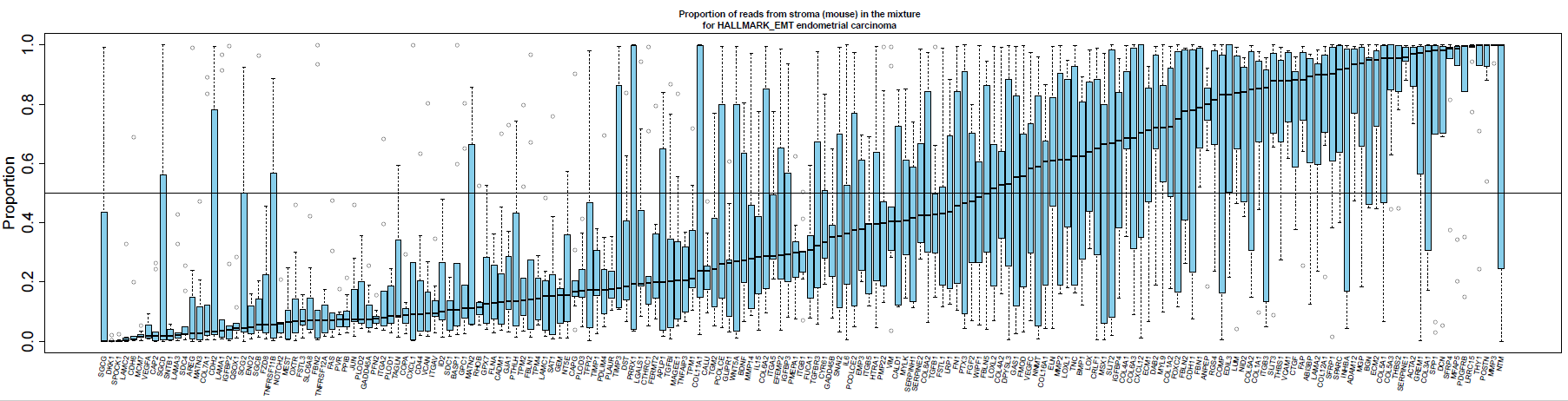

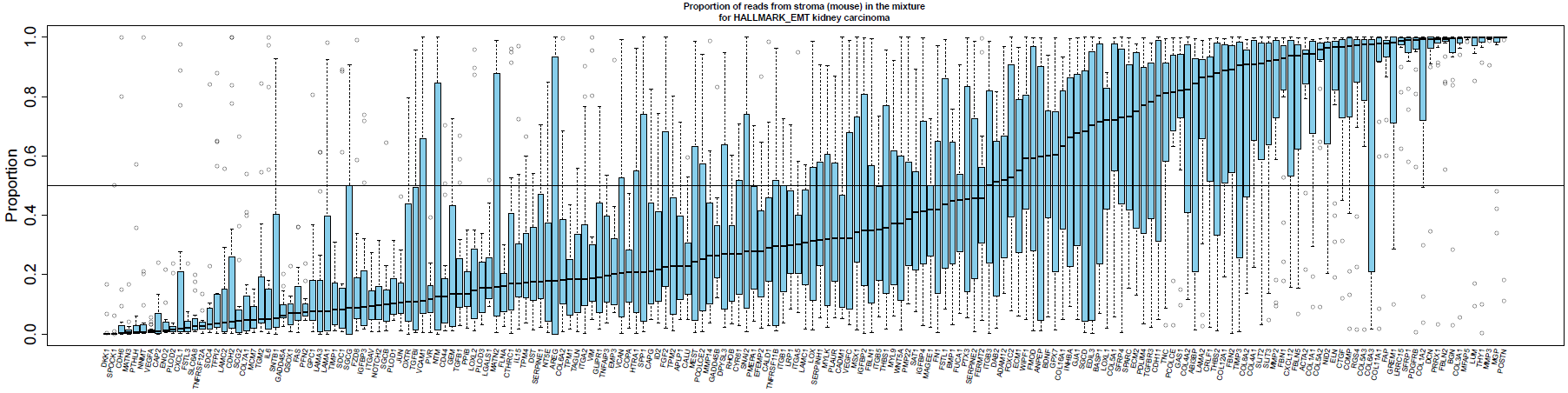

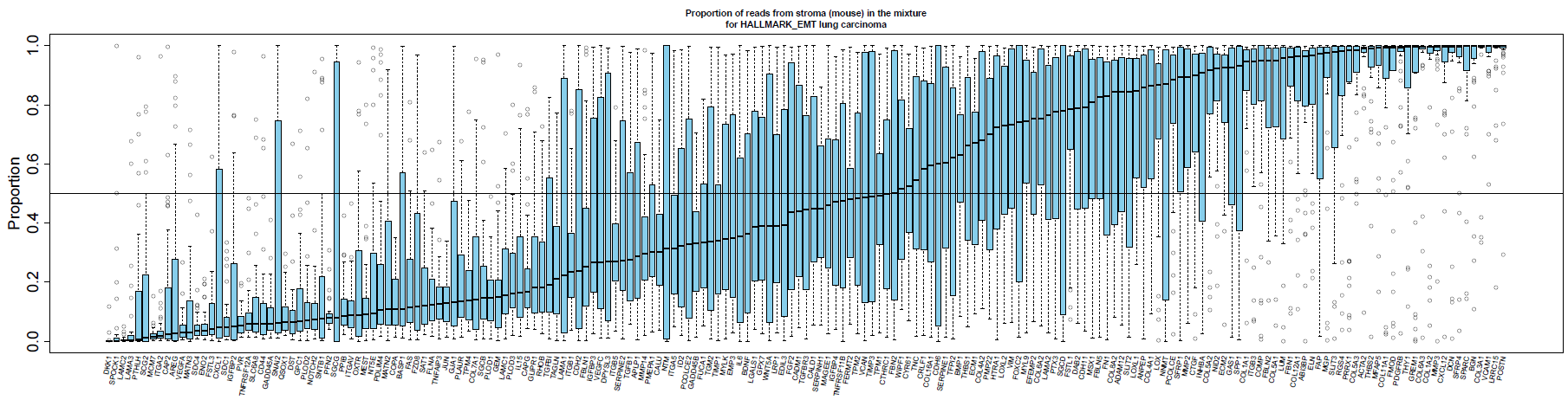

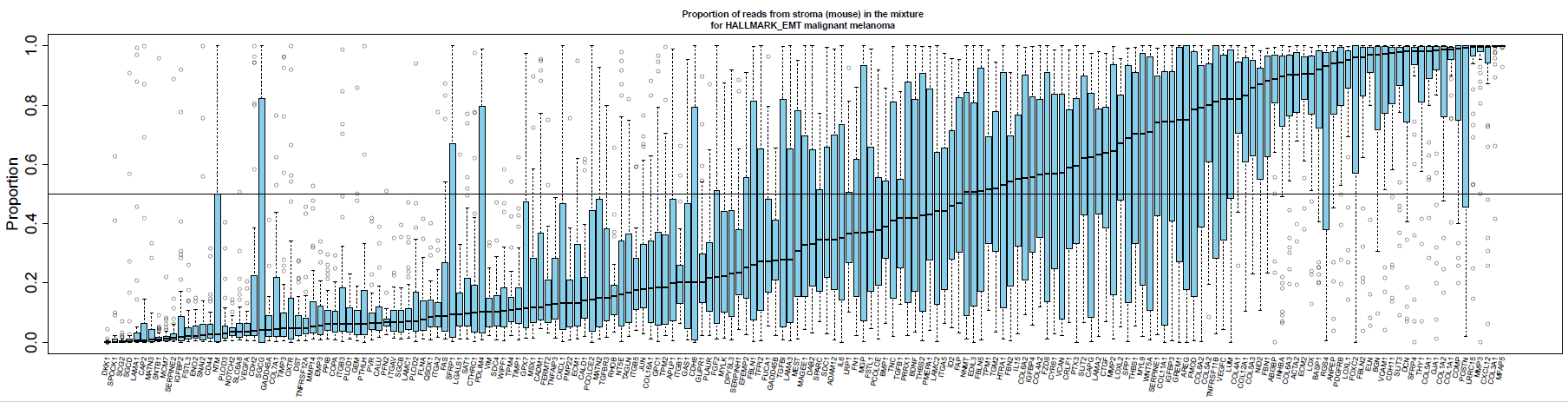

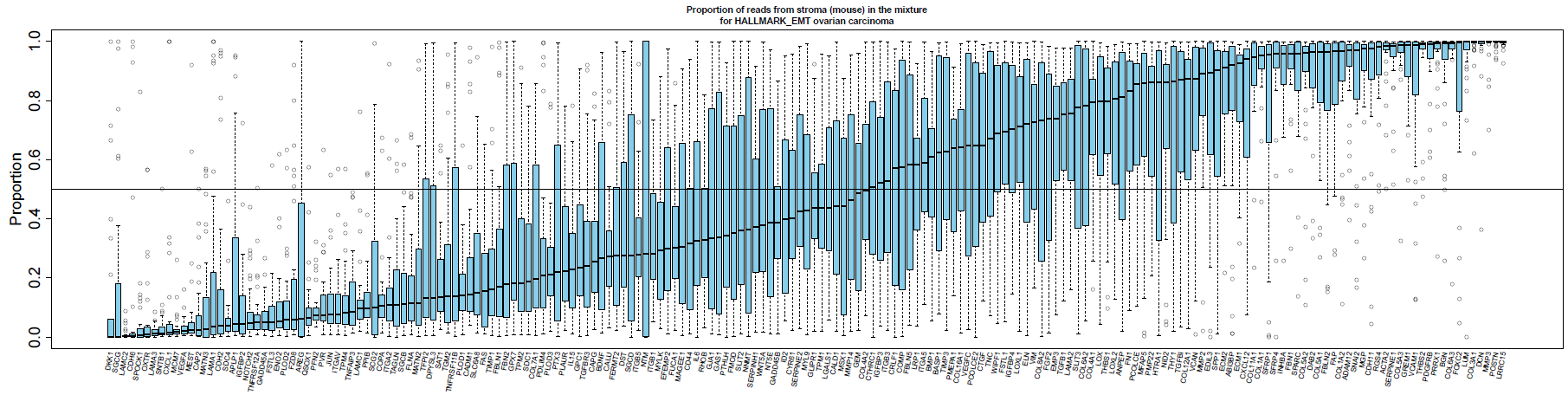

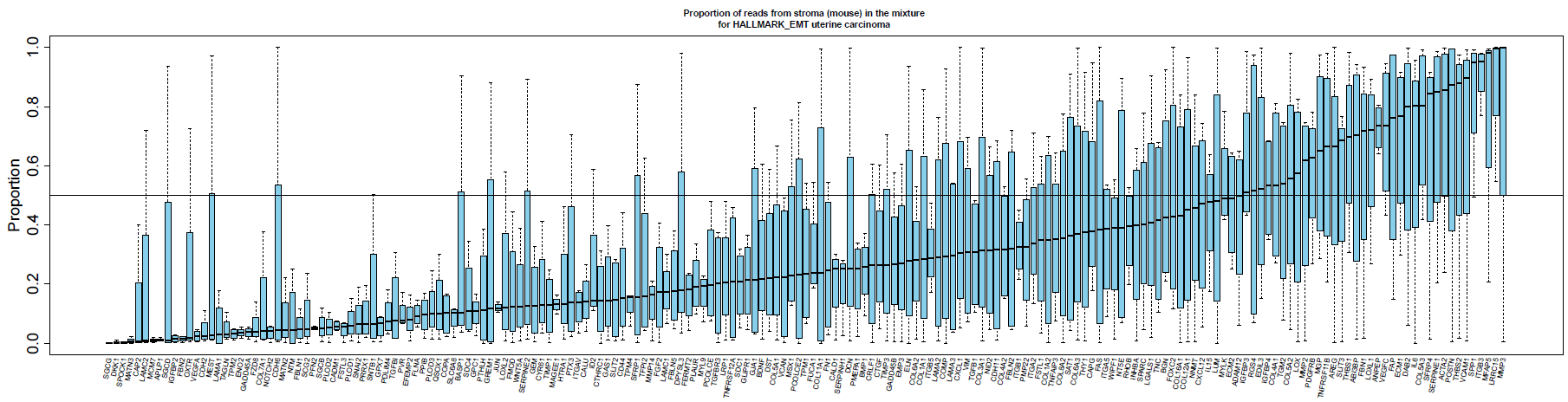


i


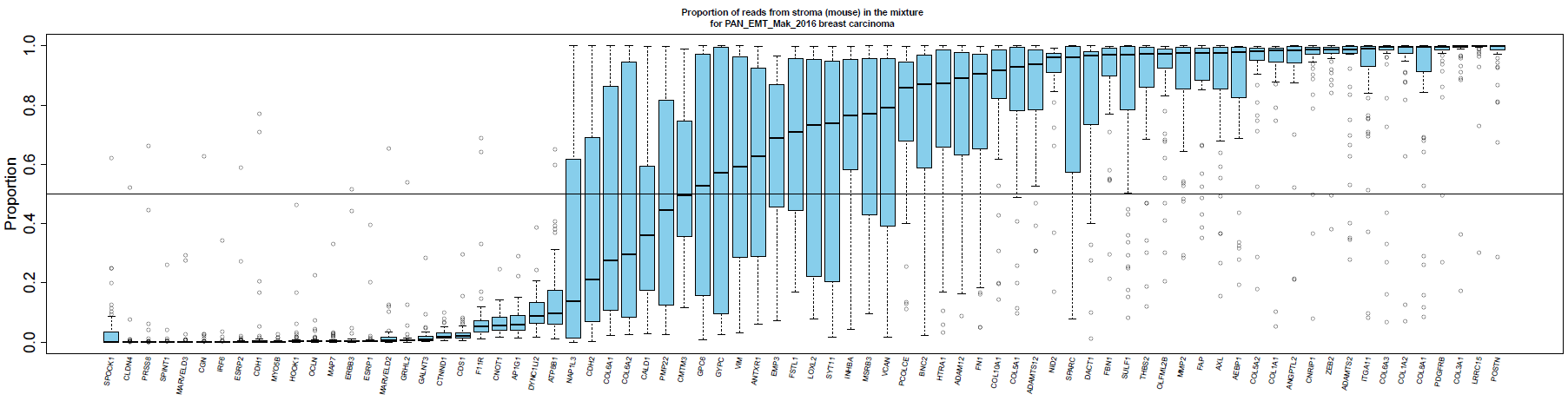

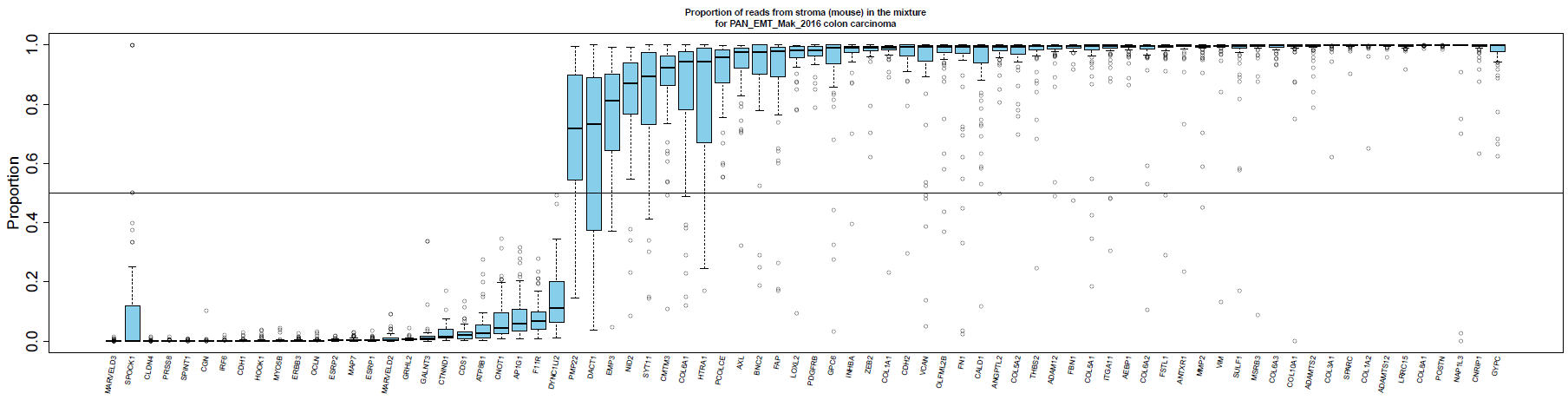

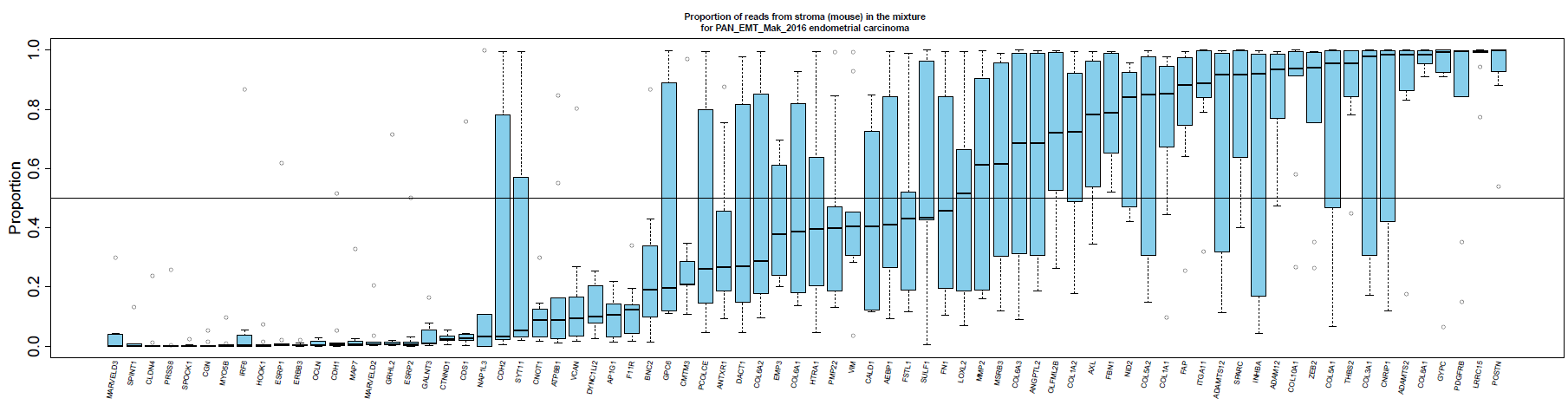

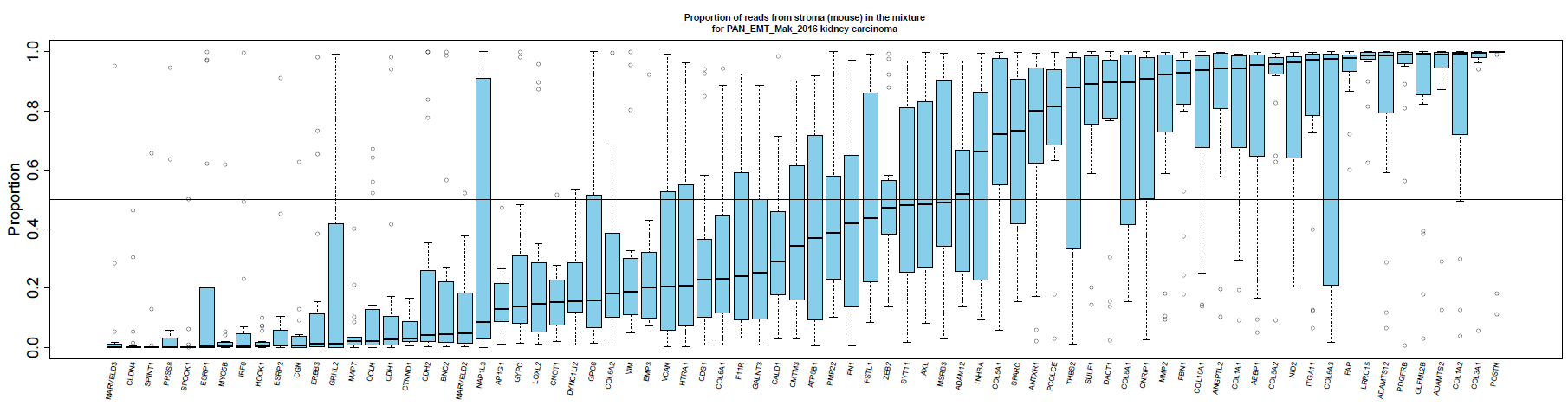

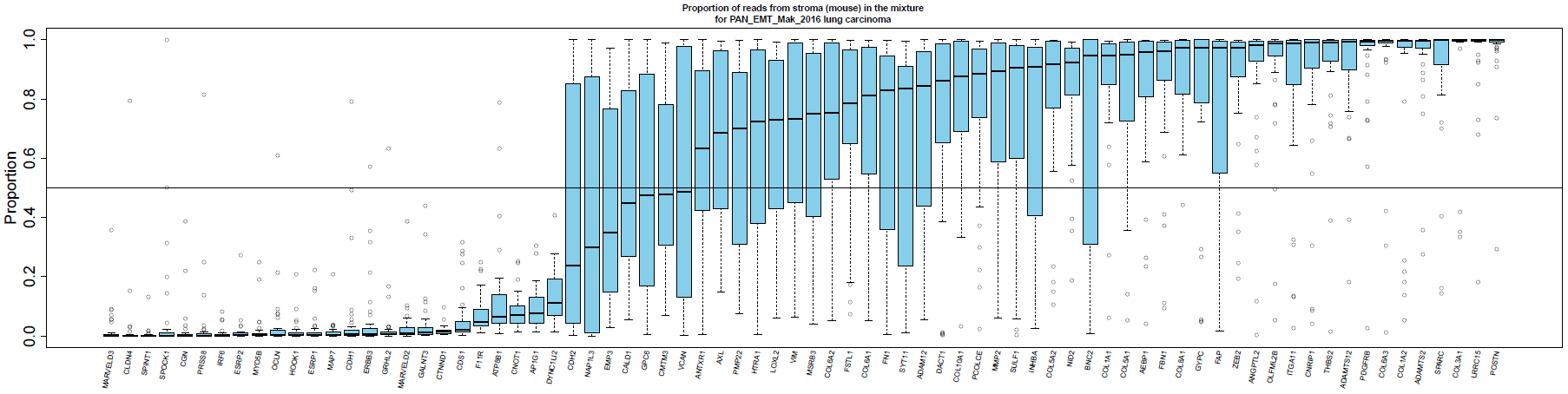

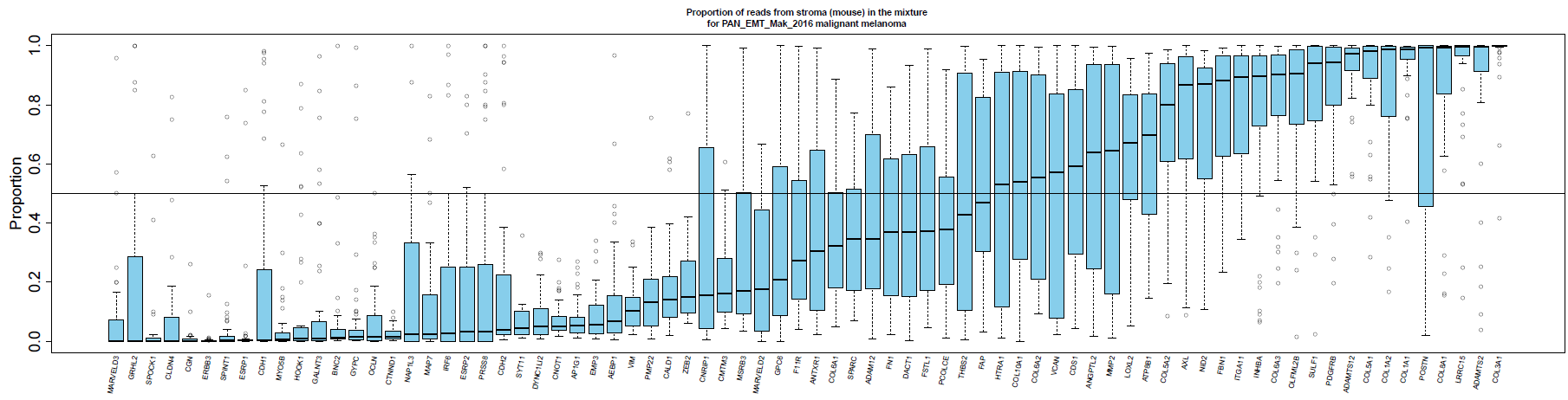


### **Supplementary Fig. 3:** EMT signature scores in fibroblasts and tumor parenchymal (epithelial) cells identified from scRNA-seq of samples from patients with colorectal or renal carcinoma. Each point represents the median score for the given gene set. High expression is observed for the canonical epithelial (*KRT7*, *KRT8*, *KRT18*, *KRT19*, *EPCAM*) and fibroblast (*COL1A1*, *COL2A1*, *DCN*, *COL4A1*) signatures in epithelial (x axis) and fibroblasts (y axis) cells, respectively. Additional canonical cell type signatures were included as a control and show low expression in fibroblast and epithelial cells, indicating high confidence in our gene signature scoring method.

### **Supplementary Fig. 4:** Correlation between EMT-related and CAF signature (BMS_FIBROBLAST) scores by tumor type in cancer datasets from TCGA. Pink plotting symbols represent tumor tissue. Aqua plotting symbols represent samples collected from non-involved healthy tissue of patients with cancer in TCGA.

a

b

c

d

e

f

g

h

i

j

k

l

m

n

o

p

q

### **Supplementary Fig. 5:** **Examples of CD8 (left) and trichrome (right-hand images) staining in tumor samples from four patients with SCCHN. a** Two examples of tumors with high parenchymal and stromal CD8+ T cell counts and low stromal collagen. **b** A tumor with low parenchymal and stromal CD8+ T cell counts and high stromal collagen. **c** Tumor with low parenchymal CD8+ T cell counts, higher stromal CD8+ T cell counts, and high stromal collagen. Brown staining indicates CD8+ T cells and blue staining indicates collagen.

a

b

c

### **Supplementary Fig. 6:** Distribution over tumor types in TCGA of estimated correlation (Pearson coefficients) between scores for TGFβ gene signatures and tumor purity, an immune gene expression signature, and a stroma signature. Boxes extend from the first to third quartiles, the middle line shows the median, and the whiskers extend to the most extreme data point that is no more than 1.5 times the IQR from the box. Consensus purity estimates were not available for esophageal carcinoma, pancreatic and stomach adenocarcinoma, and sarcoma.

### **Supplementary Fig. 7:** Cellular source of TGFβ expression in colorectal or renal cell carcinoma samples. **a** Location and expression level of GO_TRANSFORMING_GROWTH_FACTOR_BETA_BINDING signature genes in scRNA-seq datasets displayed as a 2-dimensional projection. **b** GO_TRANSFORMING_GROWTH_FACTOR_BETA_BINDING-related gene expression by cell type. Panels **a** and **b** show the gene signature scores (0–100), which represent the gene expression level for the TGFβ signatures in the scRNA-seq data.

a

b

## **Tables**

### **Supplementary Table 1:** Correlation of each EMT gene set with tumor purity, immune, and stromal signatures by tumor type

### **Supplementary Table 2:** P-values from hypothesis tests for enrichment of stroma-derived (mouse) transcripts in PDX samples for genes in each EMT gene set

### **Supplementary Table 3:** Correlation of each EMT gene set with CAF signature by tumor type

### **Supplementary Table 4:** Correlation of each EMT gene set with overall survival by tumor type

### **Supplementary Table 5:** Quantification of CD8+ cells and trichrome staining in squamous cell carcinoma of the head and neck samples
